# LGR5 marks targetable tumor-initiating cells in mouse liver cancer

**DOI:** 10.1038/s41467-020-15846-0

**Published:** 2020-04-23

**Authors:** Wanlu Cao, Meng Li, Jiaye Liu, Shaoshi Zhang, Lisanne Noordam, Monique M. A. Verstegen, Ling Wang, Buyun Ma, Shan Li, Wenshi Wang, Michiel Bolkestein, Michael Doukas, Kan Chen, Zhongren Ma, Marco Bruno, Dave Sprengers, Jaap Kwekkeboom, Luc J. W. van der Laan, Ron Smits, Maikel P. Peppelenbosch, Qiuwei Pan

**Affiliations:** 1 Department of Gastroenterology and Hepatology, Erasmus MC-University Medical Center, Rotterdam, The Netherlands; 2 Department of Surgery, Erasmus MC-University Medical Center, Rotterdam, The Netherlands; 3 Department of Cell Biology, Erasmus MC-University Medical Center, Rotterdam, The Netherlands; 4 Department of Pathology, Erasmus MC-University Medical Center, Rotterdam, The Netherlands; 5 Biomedical Research Center, Northwest Minzu University, Lanzhou, China; 6 College of Life Sciences, Zhejiang Sci-Tech University, Hangzhou, China

**Keywords:** Cancer stem cells, Targeted therapies, Liver cancer

## Abstract

Cancer stem cells (CSCs) or tumor-initiating cells (TICs) are thought to be the main drivers for disease progression and treatment resistance across various cancer types. Identifying and targeting these rare cancer cells, however, remains challenging with respect to therapeutic benefit. Here, we report the enrichment of LGR5 expressing cells, a well-recognized stem cell marker, in mouse liver tumors, and the upregulation of *LGR5* expression in human hepatocellular carcinoma. Isolated LGR5 expressing cells from mouse liver tumors are superior in initiating organoids and forming tumors upon engraftment, featuring candidate TICs. These cells are resistant to conventional treatment including sorafenib and 5-FU. Importantly, LGR5 lineage ablation significantly inhibits organoid initiation and tumor growth. The combination of LGR5 ablation with 5-FU, but not sorafenib, further augments the therapeutic efficacy in vivo. Thus, we have identified the LGR5^+^ compartment as an important TIC population, representing a viable therapeutic target for combating liver cancer.

## Abstract (web summary)

Cancer stem cells (CSCs) are thought to be the main drivers for disease progression and treatment resistance in liver cancer. This study identifies the LGR5+ compartment as an important CSC population, representing a viable therapeutic target for combating liver cancer.

## Introduction

The key concept underlying the cancer stem cell (CSC) or tumor-initiating cell (TIC) theory is that tumors are maintained through a hierarchical structure, in which different cell populations have different functionalities in pathophysiology^1^. The bulk of a tumor is thought to consist of CSCs/TICs as well as rapidly proliferating cells. CSCs/TICs are responsible for tumor initiation, resistance to conventional treatment, and distant metastasis. Rapidly proliferating cancer cells, thought to be derived from the tumor stem cell pool, are responsible for volume increment of the tumor^2^. A prediction based on this model is that ablation of the relatively small CSC compartment would ultimately result in cessation of tumor growth and metastasis, and provoke sensitization of the tumor to conventional treatment as well.

Within the framework of this theory, CSCs/TICs would be characterized by a large capacity for self-renewal, a potential for differentiation into different cell types that constitute the tumor, and a resistance to conventional treatment^1^. These key features largely overlap with those of normal stem cells, making it extremely difficult to specifically identify CSCs/TICs, but on the other hand would allow techniques traditionally used for identifying normal stem cells also to be applied for CSCs/TICs^3^. LGR5 (leucine-rich repeat-containing G protein-coupled receptor 5) evokes special interest as a potential marker for the CSC/TIC compartment in this respect. LGR5 is a well-characterized stem cell marker in several tissues/organs, including the small intestine, colon, and liver^4–6^. In the colon and intestine, the LGR5 stem cell pool constantly proliferates and differentiates into mature cell types to compensate for the loss of functional epithelial cells. Interestingly, these LGR5 stem cells also participate in the process of oncogenesis, acting as the cells-of-origin of intestinal cancer^7^. Importantly, LGR5 marks CSCs in colon cancer^8–10^, intestinal cancer^11^, and basal cell carcinoma^12^. In intestinal adenoma as well as malignant carcinoma, LGR5 cells account consistently for a ratio of 5–10% of tumor cells and fuel tumor growth^8,13^. Proof-of-concept showing that specific elimination of LGR5 cells delays tumor growth in colon cancer has been provided^9^. Given the essential role of CSCs/TICs, these cells are attractive targets for anticancer treatment, whereas their resistance to conventional therapies impedes the therapeutic development.

In contrast to the colon and intestine, LGR5 stem cells are absent in the homeostatic liver, but emerge upon tissue injury^4,14^. These liver LGR5 cells are likely to be an intermediate stem/progenitor cell population that responds to injury, but they may have a limited contribution to tissue repair^14^. Whether an LGR5^+^ compartment exists in liver cancer remains obscure, and the possible importance of such a compartment in this disease is unexplored. Nevertheless, research into this possibility is urgently needed as liver cancer is one of the most common forms of malignancy worldwide, with nearly 800,000 cases reported yearly, and it is characterized by a depressing lack of treatment options^15^. Hepatocellular carcinoma (HCC) and cholangiocarcinoma (CC) are the two main types of primary liver cancer. Currently, surgery remains the only potentially curative therapeutic strategy available, but is well-known for its high recurrence rate following tumor resection. Chemotherapy and targeted treatment are generally ineffective, with sorafenib providing some extension of life expectancy to HCC patients. The unusual treatment resistance of liver cancer is thought to be associated with the presence of CSCs/TICs, but this notion remains largely unproven^16^. Thus, we aimed to investigate whether LGR5 marks CSCs/TICs in liver cancer, and to explore the potential for therapeutic targeting of these cells. Our results show that in liver cancer, an LGR5^+^ compartment exists that is superior in tumor initiation and mediates therapy resistance. Targeting this compartment constitutes a rational avenue for combating this disease.

## Results

### Enrichment of LGR5-expressing cells in primary liver tumors

**Fig. 1:**
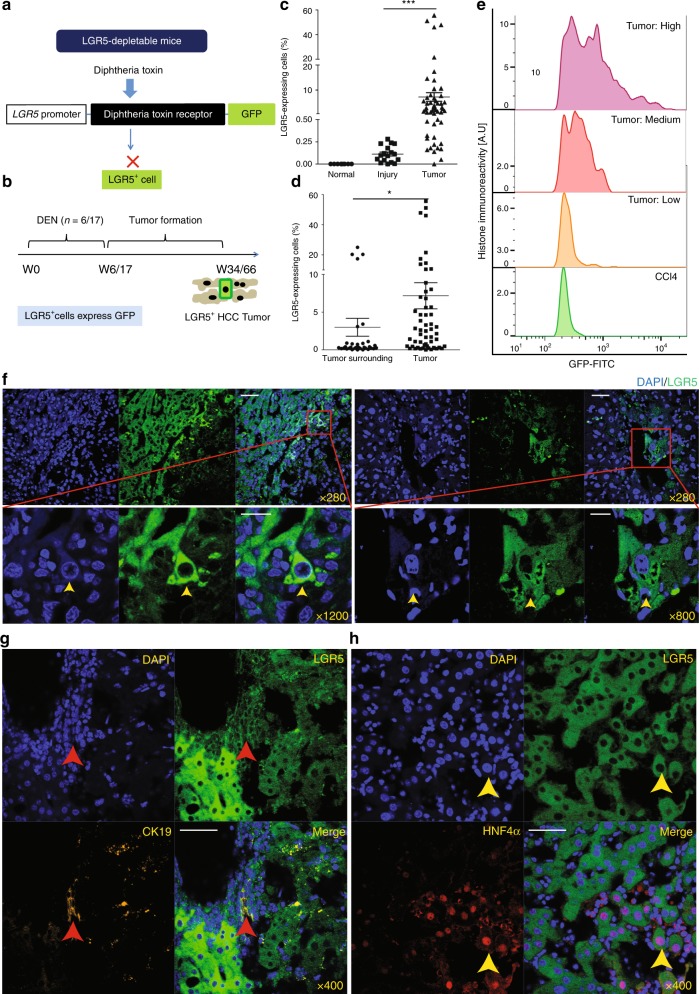
Primary murine liver tumors are enriched with LGR5-expressing cells. **a** Principle of *Lgr5–DTR–GFP* transgenic mouse strategy used in this study. **b** Principle of the experimental strategy used to induce primary murine tumors in the context of this study. **c** The percentage of LGR5^+^ cells, as determined by flow cytometry, is significantly higher in liver tumors from DEN-treated (7.29 ± 1.76%, *n* = 55) as compared with livers from untreated animals (0 ± 0%, *n* = 8) or injured livers from CCl_4_-treated animals (0.11 ± 0.022%, *n* = 17) (Welch test, *P* = 0.0001). **d** The percentage of LGR5–GFP^+^ cells is significantly increased in liver tumors (7.29 ± 1.76%, *n* = 55) as compared with the tumor-surrounding tissues (2.93 ± 1.15%, *n* = 34) of the same mice (Welch test, *P* = 0.0407). **e** Liver tumor-derived LGR5–GFP^+^ cells showed increased fluorescence intensity when compared with LGR5–GFP^+^ cells derived from CCl_4_-injured livers. **f** Representative images showing LGR5–GFP^+^ cells as present in liver tumors. Yellow arrow: LGR5–GFP^+^ cell. DAPI: blue. Upper panels: scale bar = 50 µm; lower panels: scale bar = 20 µm. **g**, **h** Representative confocal images showing the expression of the cholangiocyte marker (**g**, CK19, yellow) and the hepatocyte-specific marker (**h**, HNF4α, red) in LGR5–GFP-expressing cells, Scale bar = 50 µm. Mean ± SEM. Source data are provided as a Source Data file.

Homeostatic livers are reported to be devoid of LGR5^+^ cells, but injury does induce such cells^14^. Whether LGR5^+^ cells are present in liver cancer is largely unknown. By adopting *Lgr5–DTR–GFP* knock-in mice (Fig. 1a), we first investigated the presence of LGR5^+^ cells (GFP-co-expressing cells) in the healthy and injured liver, and during carcinogenesis. Carbon tetrachloride (CCl_4_) was used to trigger liver injury. Diethylnitrosamine (DEN) was used to induce primary liver tumor formation (Fig. 1b; Supplementary Fig. 1). Although LGR5 cells are absent in the homeostatic liver (Fig. 1c), either a single course or repeated administration of DEN can rapidly trigger the emergence of LGR5–GFP^+^ cells (post DEN induction day 7; relative size of the LGR5–GFP^+^ compartment following 1 × DEN: 0.025 ± 0.05%, *n* = 3 [mean ± SEM]; Supplementary Fig. 2a, b). Animals were monitored for liver tumor formation from 4 to 14 months post DEN induction (Supplementary Data 1). Analysis of the resulting hepatic neoplasms revealed the stable presence of an LGR5^+^ compartment in these liver tumors (Fig. 1c). The relative abundance of LGR5 cells in the tumors (Supplementary Data 1, Supplementary Fig. 2c, d) is significantly higher as compared with those in the tumor-surrounding tissues (Fig. 1d) or as detected in CCl_4_-injured livers (Fig. 1c). The LGR5 expression levels in the tumor cells show substantial variation, but are substantially and significantly higher compared with that in injured liver (Fig. 1e). Immunohistochemistry (IHC) and immunofluorescence (IF) staining of GFP expression further confirms the presence of an LGR5^+^ compartment and enables detailed analysis of spatial distribution of LGR5–GFP^+^ cells in the liver (IF: Fig. 1f; IHC: Supplementary Fig. 2e, f). Co-staining with hepatocyte marker (HNF4α) or cholangiocyte marker (CK19) revealed that a proportion of LGR5 cells in the tumor express HNF4α or CK19 (Fig. 1g, h), suggesting that LGR5^+^ cells may give rise to both a HCC-like and a CC-like phenotype, the two main types of primary liver cancer. Thus, these data have demonstrated the presence of an LGR5^+^ compartment in primary murine liver cancer.

**Fig. 2:**
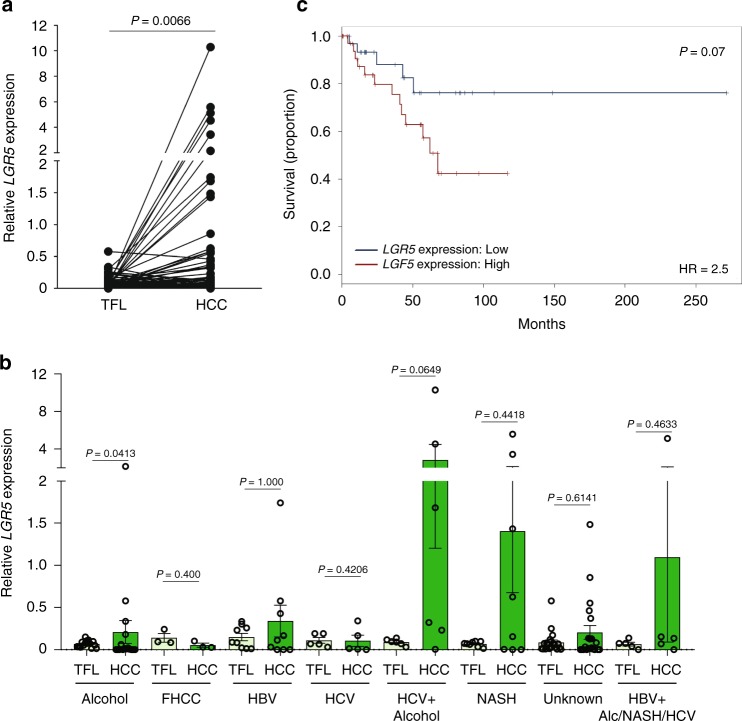
The expression of *LGR5* is upregulated in human HCC tissues. **a** Upregulation of *LGR5* expression in HCC tissues (*n* = 74) compared with tumor-free liver tissues (TFL, *n* = 75) from the Erasmus MC cohort (paired *T* test, *P* = 0.0066). *GUSB* (beta-glucuronidases), *HPRT1* (hypoxanthine phosphoribosyltransferase 1), and *PMM1* (phosphomannomutase 1) were used as reference genes for normalization. **b** The expression of *LGR5* in HCC tissues compared with TFL stratified based on the etiologies of HCC (paired *T* test). FHCC fibrolamellar carcinoma, HBV hepatitis B virus, HCV hepatitis C virus, NASH nonalcoholic steatohepatitis, Alc alcohol. Patient number: alcohol (*n* = 16); FHCC (*n* = 3); HBV (*n* = 9); HCV (*n* = 5); HCV + alcohol (*n* = 6); NASH (*n* = 8); unknown (*n* = 21); HBV + Alc/NASH/HCV (*n* = 5). **c** Kaplan–Meier curve of HCC patient survival with high (*n* = 37) and low (*n* = 37) *LGR5* expression (cutoff value based on median value—0.047). Mean ± SEM. Source data are provided as a Source Data file.

To examine the clinical relevance, we investigated *LGR5* expression in human HCC tumors from our patient cohort (Erasmus MC cohort). We found that *LGR5* expression is significantly elevated in tumor tissues compared with the paired tumor-free liver tissues (Fig. 2a), and also in some subpopulations of patients with specific etiologies of HCC (Fig. 2b). Survival analysis by predicting Kaplan–Meier curves revealed a tendency toward worse clinical outcome in patients with higher *LGR5* expression (Fig. 2c). Further analysis of online publically available datasets confirmed the upregulation of *LGR5* expression in HCC (Supplementary Fig. 3a), and possible association with clinical outcome, especially in subpopulations of specific patients (Supplementary Fig. 3b). Interestingly, with data from the TCGA database and International Cancer Genome Consortium-France (LICA-FR) and International Cancer Genome Consortium-Japan (LIRI-JP), we found that the upregulation of *LGR5* expression is more pronounced in HCC tumors with *β-catenin* mutation (Supplementary Fig. 4). This is in line with LGR5 being a *β-catenin* target gene both in the intestine and liver^5,17^. Taken together, *LGR5* cells are enriched in both mouse and human liver tumors, and bear substantial clinical relevance.

### Preservation of LGR5 cells in organoid and allograft tumors

**Fig. 3:**
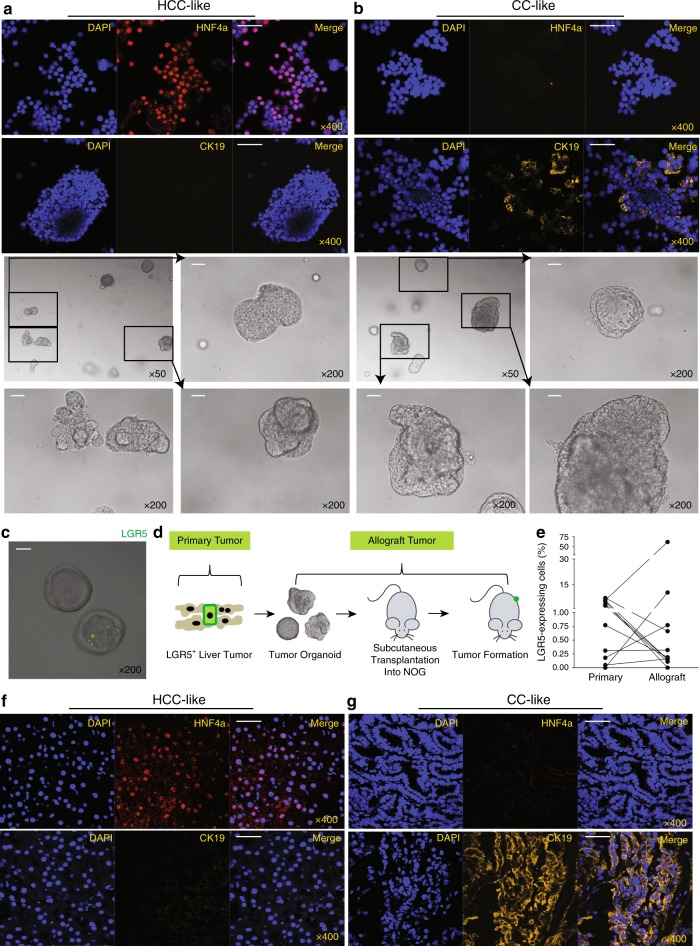
Maintenance of LGR5-expressing cells in liver tumor organoids and allograft tumors. **a**, **b** Representative pictures showing organoid lines that predominately express the hepatocyte marker HNF4ɑ (**a**) or the cholangiocyte marker CK19 (**b**) (upper panels: IF staining; lower panels: bright-field microscopic pictures, scale bar = 50 µm). **c** Representative pictures showing the presence of LGR5- expressing cells in organoids. LGR5-driven GFP: green. Scale bar = 50 µm. **d** An outline of the experimental strategy used to transplant tumor organoid lines into immunodeficient mice. **e** The percentages of LGR5-expressing cells in allograft tumors and the corresponding primary tumors (primary vs. allograft: 2.8 ± 0.8% vs. 6.8 ± 5.6%, *n* = 11, *P* = 0.3577). **f**, **g** Representative pictures of allograft tumors that mainly express either the hepatocyte marker HNF4ɑ (**f**) or the cholangiocyte marker CK19 (**g**). Scale bar = 50 µm. Mean ± SEM. Source data are provided as a Source Data file.

3D organoid cultures are robust model systems for studying the properties of (cancer) stem cells^18–20^. We have successfully established routine procedures^21^ for creating organoid cultures from primary liver tumors of DEN-induced mice (Supplementary Fig. 1). In total, 89 tissues were obtained from 41 individual murine livers (Supplementary Data 1). In all, 63 out of 89 (70.8%) tumor/tumor-surrounding tissues successfully initiated organoids (8 out of 34 tumor-surrounding tissues did not initiate organoids, 23.5%; 18 out of 55 tumor tissues did not initiate organoids, 32.7%). These organoids can be maintained and propagated in 3D culture for at least 5 months. Staining for CK19 and HNF4α demonstrates that these organoids display either a CC or HCC-like phenotype (Fig. 3a, b). Importantly, these cultured organoids maintain a population of LGR5-positive cells (Fig. 3c).

To investigate whether these murine organoid lines are malignant, we transplanted all the 63 strains into immunodeficient NOG mice (Fig. 3d). One to 6 months after allografting, 11 out of 63 organoid strains formed palpable tumors in the immune-deficient mice (17.5%). All contained an LGR5–GFP^+^ compartment as determined by FACS analysis of the tumors (Fig. 3e).

Following re-culture of cells obtained from these allograft tumors as organoids, we observed substantial diversity of the morphology (Supplementary Fig. 5a–c) and CK19/HNF4α expression in the corresponding allograft tumors (Fig. 3f; Supplementary Data 2). A population of LGR5-expressing cells were again observed in these organoid cultures (Supplementary Fig. 5d), in line with the existence of such a compartment in the allograft tumors from which these organoid cultures originated (IF: Supplementary Fig. 5e; IHC: Supplementary Fig. 5f and Supplementary Data 2). In addition, genome-wide transcriptomic analysis revealed a distinct gene expression signature between LGR5^+^ and LGR5^−^ cells, including TATA-box binding protein-associated factor 7 like (*Taf7l*), sialophorin (*Spn*), SRY-box 2 (*Sox2*), nidogen-1 (*Nid1*), paralemmin 3 (*Palm3*), alpha-1-microglobulin/bikunin precursor (*Ambp*), membrane-bound O-acyltransferase domain containing 4 (*Mboat4*), and chymase 1 (*Cma1*), which had higher expression levels in LGR5^+^ compared with LGR5^−^ population. Kaplan–Meier curve analysis revealed that all these genes are associated with the survival of liver cancer patients (Supplementary Fig. 6, Supplementary Data 3–4). Especially, *Sox2* as a transcription factor is essential for maintaining self-renewal or pluripotency of undifferentiated embryonic stem cells, and has been reported as a marker for cancer stem cells in breast cancer and squamous-cell carcinoma^22^. Gene enrichment analysis of the 196 differentially expressed genes further revealed the involvement of metabolism-related pathways, including “Oxidation by Cytochrome P450”, “Calcium Regulation”, “Metapathway biotransformation”, and “Purine metabolism”. There are also differentially expressed genes involved in immune regulation, including “Macrophage markers pathway”, “Kit Receptor Signaling Pathway”, and “IL-6 signaling Pathway”. Furthermore, LGR5^+^ cells had significantly differentially expressed genes involved in cell proliferation/migration, including “Chemokine signaling pathway”, “Matrix Metalloproteinases”, and “PPAR signaling pathway”. Interestingly, there are differentially expressed genes enriched in “Wnt Signaling Pathway” and “G Protein Signaling Pathways”. Subsequent experiments were initiated to assess the exact functionality of LGR5- expressing cells.

### LGR5^+^ cells are superior in organoid and tumor initiation

**Fig. 4:**
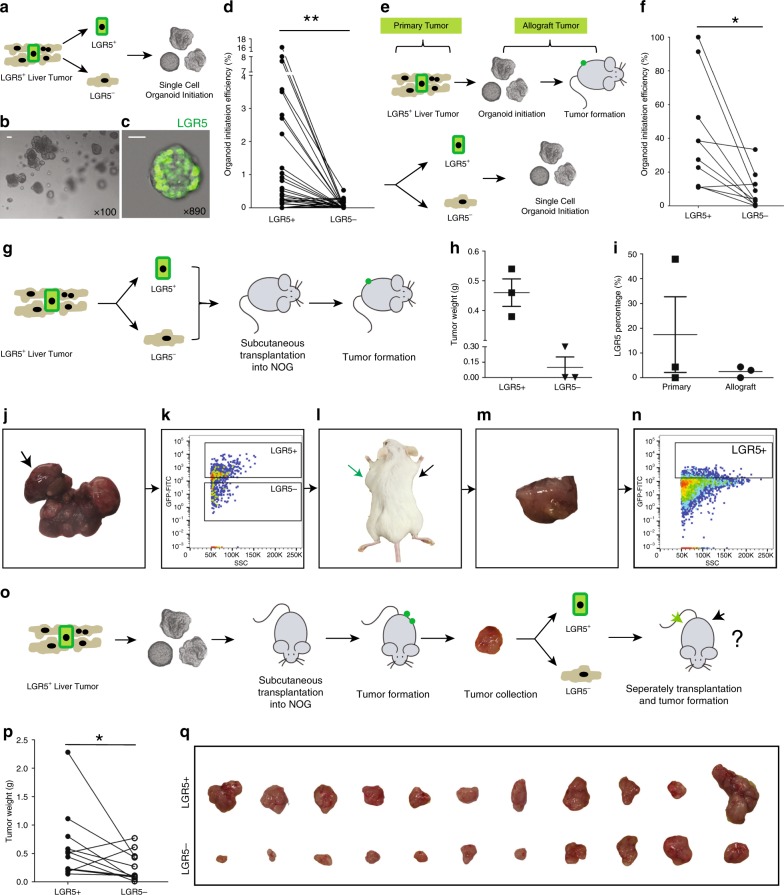
Single LGR5^+^ cells from liver tumors are superior in organoid and tumor initiation. **a** An outline of the experimental strategy for studying ex vivo organoid initiation of cells derived from primary murine liver tumors. **b** A representative picture of organoids derived from single LGR5^+^ cells. Scale bar = 50 µm. **c** Representative confocal micrograph of a single LGR5^+^ cell-initiated organoid dominated by LGR5-expressing cells. LGR5-driven GFP: green. Scale bar = 20 µm.  **d** Organoid initiation efficiency of LGR5–GFP^+^ and LGR5–GFP^−^ cells, isolated from primary tumors (LGR5^+^ cells: 25 out of 71 tissues, 35.2%; LGR5^−^ cells: 11 out of 71 tissues, 15.5%) (paired *T* test, 2.13 ± 0.67% vs. 0.065 ± 0.023%, *n* = 30, *P* = 0.0048). **e** An outline of the strategy used to study ex vivo organoid initiation of allograft tumor-derived cells. **f** Efficiency of organoid initiation by allograft liver tumor-derived LGR5–GFP^+^ and LGR5–GFP^−^ cells (paired *T* test, 40.46 ± 10.19% vs. 9.84 ± 3.93%, *n* = 10, *P* = 0.0187). **g** Outline of the experimental strategy used to assess in vivo tumor initiation of cells isolated from primary murine liver tumors. **h** Weight of tumors initiated by LGR5^+^ and LGR5^−^ cells (LGR5^+^ vs. LGR5^−^: 0.46 ± 0.046 g vs. 0.10 ± 0.10 g, *n* = 3) (formed tumor number: LGR5^+^ cells—3 out of 9; LGR5^−^ cells—1 out of 9). **i** LGR5 expression in single LGR5^+^ cell-derived allograft tumors and the corresponding primary tumors (17.42 ± 15.29% vs. 2.47 ± 1.27%, *n* = 3). **j**–**n** Representative pictures showing that LGR5–GFP^+^ and LGR5–GFP^−^ cells (**k**) were isolated from DEN-induced primary liver tumors (**j**). Then, LGR5–GFP^+^ cells (green arrow) initiated allograft tumors in immunodeficient mouse (**l**–**n**). The initiated allograft tumors sustained LGR5 expression (**n**). **o** An outline of the experimental strategy for in vivo tumor initiation assay of cells isolated from allograft murine liver tumors. **p**, **q** Tumor weight (**p**) and macroscopic aspect (**q**) of allografts initiated by LGR5–GFP^+^ cells and LGR5–GFP^−^ cells (isolated from allograft tumors) (0.64 ± 0.19 g vs. 0.27 ± 0.08 g, *n* = 11, *P* = 0.0418). Mean ± SEM. Source data are provided as a Source Data file.

For functional comparison of LGR5^+^ and LGR5^−^ liver cancer cells, we first assessed their relative clonogenic ability using an organoid initiation assay. Employing FACS (the sorting strategy: Supplementary Fig. 7a), LGR5–GFP^+^ and LGR5–GFP^−^ cells were collected from 71 individual primary murine liver tissues, and cultured in 3D matrigel (Fig. 4a; Supplementary Data 5). After 2–3 weeks, we observed organoid formation from single cells (Fig. 4b–d). Importantly, LGR5–GFP^+^ cells have stronger organoid formation ability compared with LGR5–GFP^−^ cells (2.13 ± 0.67% vs. 0.07 ± 0.02%, *n* = 30) (Supplementary Data 5: detailed organoid initiation efficiency). In addition, we also observed that the initiation ability of LGR5^+^ cells showed close correlation to collected cell number. The average numbers of LGR5^+^ cells collected from tissues that did initiate organoid (1906 ± 442, *n* = 25) were significantly higher compared with the number that did not (171 ± 47, *n* = 46). This was not the case for LGR5^−^ cells (28350 ± 8914, *n* = 60 vs. 13860 ± 3654, *n* = 11) (Supplementary Fig. 7b–d). This indicates that a sufficient cell number (>1000) is essential for successful organoid initiation of LGR5-expressing cells from liver tumors.

We next performed organoid initiation for cells derived from the allograft tumors (Fig. 4e). Similar as observed with primary tumors, LGR5^+^ cells of allograft tumors initiate more organoids as compared with LGR5^−^ cells (40.5 ± 10.2% vs. 9.8 ± 3.9%, *n* = 10) (Fig. 4f). Compared with cells isolated from primary tissues, allograft tumor cells are more potent with respect to their potential for organoid initiation (Supplementary Fig. 7e–g). Interestingly, organoids formed from a single LGR5–GFP^+^ or LGR5–GFP^−^ cell produce both LGR5-positive and -negative offspring, suggesting possible self-formation of a hierarchical organization sustaining organoid growth and differentiation (Supplementary Fig. 7h).

The ultimate measure of potential functionality of LGR5^+^ cells in pathophysiology is their capacity to form allograft tumors in vivo (Fig. 4g). Hence, identical numbers of FACS-sorted LGR5–GFP^+^ and LGR5–GFP^−^ cells derived from primary liver tumors were subcutaneously engrafted into NOG mice, and tumor formation was monitored (Supplementary Data 6). As expected, LGR5^+^ cells display a stronger capacity for tumor initiation as compared with LGR5^−^ cells (LGR5^+^ vs. LGR5^−^: 33.3% vs. 11.1%) (Fig. 4h; Supplementary Data 6). Moreover, tumors initiated from LGR5^+^ cells contain both LGR5-positive and -negative populations (Fig. 4i–n). In addition, we have performed a tumor formation assay for the cells that were derived from the allograft tumors (Fig. 4o). Again, the LGR5^+^ compartment proved markedly more potent in this respect relative to the LGR5–GFP^−^ compartment (Fig. 4p, q; Supplementary Table 1). Collectively, our results are best interpreted that liver tumor-derived LGR5^+^ cells constitute a bona fide TIC compartment.

### Anticancer treatment enriches LGR5-expressing cells

**Fig. 5:**
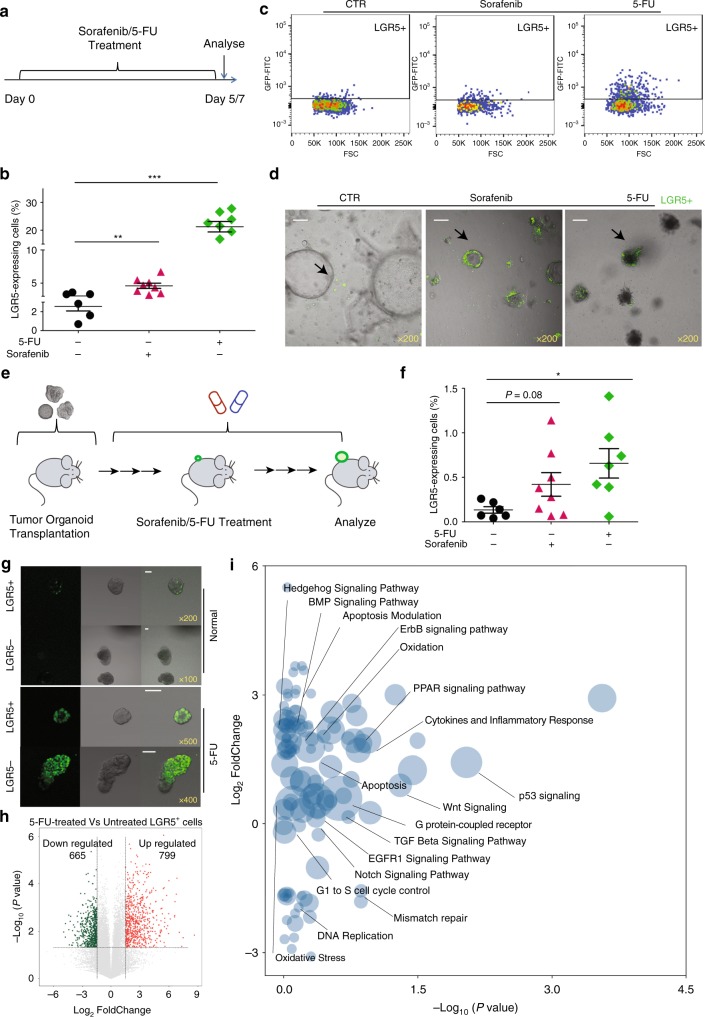
Anticancer treatment selects for LGR5^+^ cells. **a** Outline of the ex vivo experimental strategy used to assess the effects of drug treatment on the size of the LGR5^+^ compartment. **b** The fraction of LGR5–GFP^+^ cells is significantly increased upon treatment with sorafenib or 5-FU (vehicle control vs. 10 µM sorafenib vs. 10 µM 5-FU: 2.6 ± 0.5% vs. 4.6 ± 0.4% vs. 21.3 ± 1.9%). **c**, **d** Representative FACS plots (**c**) and confocal pictures (**d**) demonstrating that the fraction of LGR5–GFP^+^ cells is increased upon treatment with sorafenib or 5-FU, scale bar = 50 µm. **e** An outline of the experimental strategy used for testing the effects of drug administration in vivo. **f** The percentages of LGR5–GFP^+^ cells is increased upon administration of sorafenib or 5-FU to allografted animals (vehicle control vs. sorafenib vs. 5-FU: 0.13 ± 0.04%, *n* = 6 vs. 0.42 ± 0.13 %, *n* = 8 vs. 0.66 ± 0.17%, *n* = 7). **g** Representative confocal pictures showing that both single LGR5–GFP^+^ and LGR5–GFP^−^ cell-initiated organoids contain LGR5-expressing cells, and the relative fraction of LGR5-expressing cells is increased in treatment-resistant organoids. LGR5-driven GFP: green, scale bar = 50 µm. **h** A volcano plot showing the most significantly differentially expressed genes between 5-FU treated/untreated LGR5^+^ cells. **i** Gene enrichment analysis (with the library of Wiki2019) within the differentially expressed genes. Mean ± SEM. Source data are provided as a Source Data file.

CSCs or TICs are presumed to be relatively resistant to conventional anticancer treatment. A prediction would thus be that in liver cancer, the LGR5^+^ cells would be more resistant to anticancer treatment as compared with the LGR5^−^ cells. Hence, we challenged tumor organoids with sorafenib, the FDA-approved drug for treating advanced HCC, and compared the relative potential of LGR5–GFP^+^ and LGR5–GFP^−^ cells to withstand such treatment (Fig. 5a). Treatment of tumor organoids with sorafenib significantly increased the percentage of LGR5-positive cells in the population (Fig. 5b–d). This effect became even more profound when the organoids were treated with the chemotherapeutic agent, 5-fluorouracil (5-FU) (Fig. 5a–d).

Subsequently, the relative size of the LGR5^+^ compartment following in vivo treatment with these therapeutic agents was assessed (Fig. 5e). Treatment with either sorafenib or 5-FU to mice-bearing allograft tumors, formed by engrafting tumor organoids, substantially increased the fraction of the LGR5–GFP^+^ cells in the tumors (Fig. 5f). Also when LGR5–GFP^+^ and LGR5–GFP^−^ cells were isolated from tumor organoids and used for organoid re-initiation, while subsequently being treated with 5-FU, the resulting cultures were dominated by LGR5–GFP^+^-expressing cells, independent from whether LGR5–GFP^+^ or LGR5–GFP^−^ were used as starting material (Fig. 5g). Of note, LGR5^+^ cells isolated from 5-FU-treated tumors retained the ability of organoid and tumor initiation (Supplementary Fig. 8). Interestingly, 5-FU treatment effectively rewired the transcriptome of LGR5^+^ cells (Fig. 5h; Supplementary Figs. 6 and 9). Gene enrichment analysis of the 1464 differentially expressed genes between 5-FU treated compared with untreated LGR5^+^ cells revealed the involvement of stem cell-related pathways, including “Wnt Signaling”, “Notch Signaling Pathway”, “ErbB signaling pathway”, “Hedgehog Signaling Pathway”, and “BMP Signaling Pathway” (Fig. 5i; Supplementary Data 3). These pathways are commonly activated in many types of solid tumors, associated with cancer initiation, progression, and metastasis^23^. Interestingly, there are several pathways, including “TGF Beta Signaling Pathway”, “EGFR1 Signaling Pathway”, “PPAR signaling pathway”, “G1 to S cell cycle control”, “Mismatch repair”, “p53 signaling”, and “Apoptosis Modulation/Apoptosis pathway”, which are known to be implicated in anticancer treatment response and DNA damage response^24^. These results may partially explain the enrichment of LGR5-expressing cells upon 5-FU treatment. In conclusion, besides resistance, conventional anticancer treatment also triggers the generation and propagation of LGR5-expressing cells.

### LGR5 lineage ablation inhibits organoid and tumor growth

**Fig. 6:**
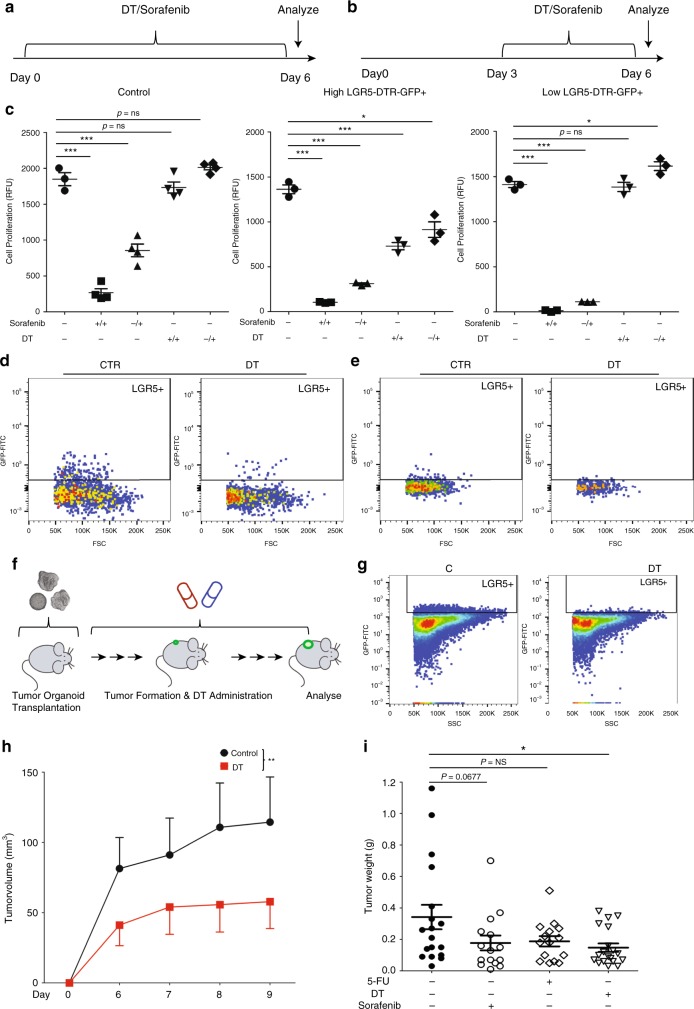
LGR5 lineage ablation inhibits organoid and tumor growth. **a**, **b** The outlines of the ex vivo experimental strategy to assess the effects of anticancer drug treatment on organoid initiation, and delineate its temporal aspect (**a**) or during organoid expansion (**b**). **c** The response of wild-type tumor organoids (left) and *Lgr5–DTR–GFP* mice-derived tumor organoids, with relatively high LGR5 expression (the percentage of LGR5 expression is greater than 1%) (middle) or low LGR5 expression (the percentage of LGR5 expression is lesser than 1%) (right) during regular expansion to DT/sorafenib treatment. −/+: drug treatment during the expansion period; +/+: drug treatment since the initial culture day (unpaired *T* test). **d**, **e** Representative FACS plots showing that LGR5–GFP^+^ cells are depleted by DT treatment, for high LGR5 expression organoid strains (**d**) and low LGR5 expression organoid strains (**e**). **f** Outlines of the experimental strategy used to assess the efficacy of DT/sorafenib/5-FU administration on allograft tumors in mice. **g** Representative FACS plots from experiments validating the strategy to deplete LGR5^+^ cells. **h** A representative growth curve showing the volumes of tumors derived from the vehicle control group and the DT-administered group (*n* = 8, two-way ANOVA). **i** The weight of tumors from vehicle control, DT, 5-FU, or sorafenib-treated groups, on the day of mice sacrifice (control vs. sorafenib vs. 5-FU vs. DT: 0.34 ± 0.078 g, *n* = 18 vs. 0.18 ± 0.047 g, *n* = 15 vs. 0.19 ± 0.033 g, *n* = 15 vs. 0.15 ± 0.027 g, *n* = 19). Mean ± SEM. Source data are provided as a Source Data file.

From the results described above, we inferred that ablation of the LGR5^+^ compartment should impair liver cancer growth. To experimentally test this notion, we exploited the co-expression of the diphtheria toxin receptor (DTR) in the *Lgr5–DTR–GFP* mice. This would allow us to specifically deplete the Lgr5–DTR–GFP^+^ compartment through diphtheria toxin (DT) administration (Fig. 1a). We have optimized the concentrations of DT treatment (1–10 ng/ml) for LGR5 depletion, with organoids derived from healthy *Lgr5–DTR–GFP* mice^14^. Accordingly, we evaluated the effects on organoid initiation and proliferation (Fig. 6a, b), and sorafenib treatment served as a positive control. DT treatment inhibited the growth of tumor organoids in an effect that showed close correlation as to the effects on the numbers of LGR5–GFP^+^ cells (Fig. 6c–e). DT treatment did not influence the growth of tumor organoids of genetically wild-type (Fig. 6c: left panel).

We further assessed therapeutic targeting of LGR5 liver cancer cells in vivo. We first evaluated the effect of DT treatment after formation of visible tumors, following transplantation of tumor organoids into immunodeficient mice (Supplementary Fig. 10a). 5-FU and sorafenib served as the positive controls. The effects of LGR5 cell depletion on the growth of formed tumors was minor (Supplementary Fig. 10c: right panel and 10d: right panel). In contrast, administration of DT immediately after transplantation of tumor organoids (Fig. 6f) efficiently delayed tumor initiation and inhibited their growth (Fig. 6h; Supplementary Fig. 10c: left panel and 10d: left panel). Further analysis of the tumors confirmed the depletion of the LGR5–GFP^+^ compartment in the DT-treated animals (Fig. 6g). Using absolute tumor size as a measure, DT-mediated depletion of the LGR5^+^ compartment impaired tumor growth (Fig. 6i). The enrichment of stem cell markers also differed in control and DT-treated LGR5^+/−^ cells (Supplementary Fig. 11). Interestingly, there was an inverse correlation between tumor and the relative size of the LGR5–GFP^+^ compartment at the end of the experiment (Supplementary Fig. 10e, f), indicating that LGR5-expressing cells are probably more active in the tumor initiation period. As control, DT treatment did not influence initiation and growth of tumors formed from the wild-type tumor organoids (Supplementary Fig. 12a, b). Thus, LGR5 lineage ablation impedes organoid and tumor initiation and further growth.

### Combination therapy enhances the anticancer efficacy

As LGR5^+^ cells appear to mediate resistance against conventional anticancer treatment, it is rational to evaluate the combination of LGR5^+^ lineage ablation with conventional anticancer treatment.

**Fig. 7:**
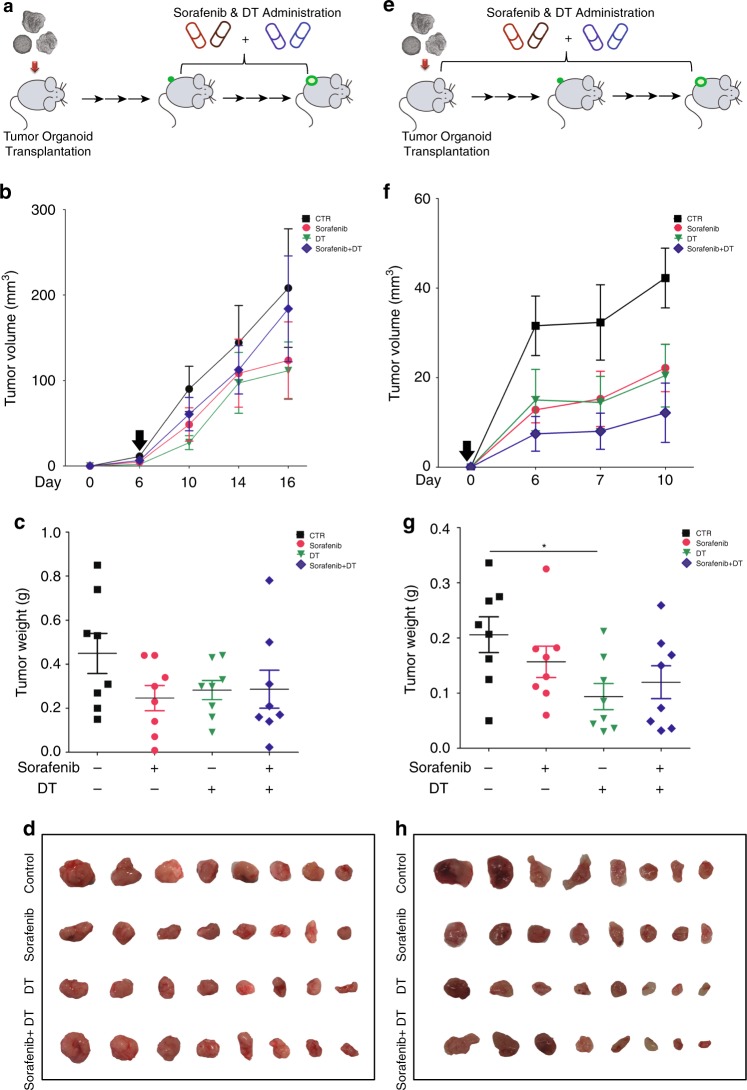
Combination of LGR5 lineage ablation with sorafenib does not enhance the efficacy. **a** Outline of the experimental strategy to assess the combinatory effect of LGR5 lineage ablation with sorafenib. Sorafenib and DT were administered every other day for in total 10 days since visualization of tumor formation after organoid engraftment. **b** Representative growth curves showing tumor volumes in the vehicle control (CTR), sorafenib, DT, and sorafenib + DT-treated groups. Black arrow: onset of administration. **c** Tumor masses from these four groups (CTR vs. sorafenib vs. DT. vs. sorafenib + DT: 0.45 ± 0.09 g, *n* = 8 vs. 0.25 ± 0.06 g, *n* = 8 vs. 0.28 ± 0.043 g, *n* = 8 vs. 0.29 ± 0.09, *n* = 8). **d** Images showing tumors from these four groups. **e** Outlines of the experimental strategy for assessing the effects of combining LGR5 lineage ablation and sorafenib treatment. Sorafenib, DT, or the combination were administered immediately since transplantation of the organoids every other day, for in total 10 days. **f** Representative growth curves showing tumor volumes of the four groups. Black arrow: onset of administration. **g** The tumor masses of these four groups (CTR vs. sorafenib vs. DT vs. sorafenib + DT: 0.21 ± 0.03 g, *n* = 8 vs. 0.16 ± 0.03 g, *n* = 8 vs. 0.09 ± 0.02 g, *n* = 8 vs. 0.12 ± 0.03 g, *n* = 8). **h** Images showing the tumors from the different groups. Mean ± SEM. Source data are provided as a Source Data file.

**Fig. 8:**
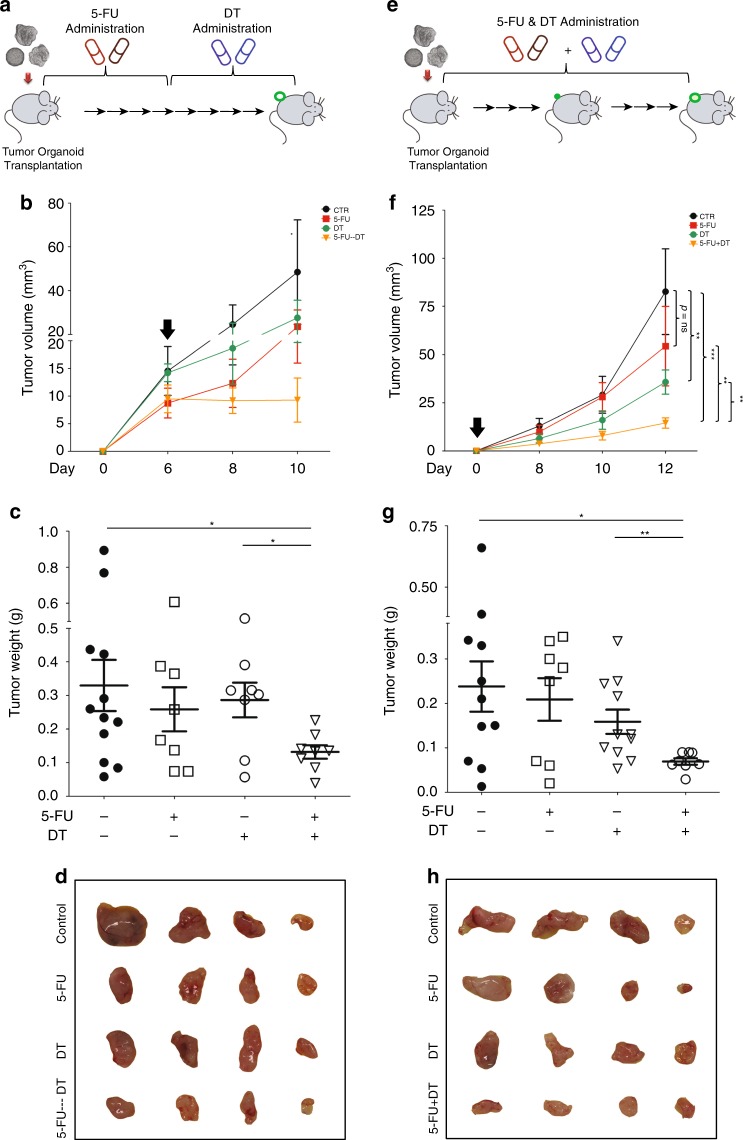
Combination of LGR5 lineage ablation with 5-FU results in enhanced anticancer efficacy. **a** Outline of the experimental strategy to assess the combinatory effect of LGR5 lineage ablation with 5-FU. Following tumor organoid allografting, 5-FU was administered for the first half of the experiment (every other day, for in total 6 days). DT was administered for the second half of the experiment (every other day, for in total 6 days). **b** Representative growth curves showing tumor volumes in the vehicle control group (CTR), the 5-FU monotherapy group, the DT administration-only group, and the hybrid 5-FU/DT group. Black arrow: onset of DT administration. **c** Tumor masses from these four groups (control vs. 5-FU vs. DT vs. 5-FU-DT: 0.33 ± 0.076 g, *n* = 12 vs. 0.25 ± 0.066 g, *n* = 8 vs. 0.29 ± 0.052 g, *n* = 8 vs. 0.13 ± 0.020 g, *n* = 8). **d** Representative images showing tumors from these four groups. **e** Outlines of the experimental strategy for assessing the effects of combined LGR5 lineage ablation and 5-FU treatment. 5-FU, DT, or the combination were administered since organoid engraftment every other day, for in total 12 days. **f** Representative growth curves showing tumor volumes of the four groups. Black arrow: onset of administration (two-way ANOVA). **g** The tumor masses of these four groups (control vs. 5-FU vs. DT vs. 5-FU + DT: 0.24 ± 0.056, *n* = 11 vs. 0.21 ± 0.048 g, *n* = 8 vs. 0.16 ± 0.027 g, *n* = 11 vs. 0.069 ± 0.007 g, *n* = 8). **h** Representative images showing the tumors from the different groups. Mean ± SEM. Source data are provided as a Source Data file.

To experimentally test this notion, we first combined DT with sorafenib treatment. However, with different strategies of combination therapy, no enhanced antitumor activity was observed on allografted tumors (Fig. 7). We next tested the combination of 5-FU and DT. Allograft tumor-bearing mice were first subjected to 5-FU (which increases the relative size of the LGR5^+^ compartment) followed by cessation of 5-FU therapy and start of DT treatment as to kill the LGR5^+^ cells (Fig. 8a; Supplementary Fig. 13a–c). Indeed, this approach is effective in combating allograft tumor formation (Fig. 8b) and is substantially superior to monotherapy with 5-FU, stand-alone DT treatment (Fig. 8c, d; Supplementary Fig. 14a) or initial treatment with DT followed by 5-FU therapy (Supplementary Fig. 13d–h). Simultaneous administration of 5-FU and DT (Fig. 8e) also resulted in anticancer effects (Fig. 8f–h; Supplementary Fig. 14b). Thus, targeting the LGR5^+^ compartment markedly enhances the efficacy of conventional treatment aimed at combating liver cancer.

## Discussion

This study has demonstrated that liver cancer contains an LGR5^+^ compartment that has various hallmarks of TICs/CSCs, including an increased capacity for tumor organoid formation in culture and allograft formation in mice, as well as resistance against conventional anticancer therapy. Functionally, these cells seem more important in tumor initiation, whereas the LGR5^−^ compartment appears to bear the proliferative burden. Simultaneously targeting both compartments as demonstrated in this study by 5-FU treatment in combination with DT-mediated ablation of the LGR5^+^ compartment was effective in combating liver cancer in experimental models. Although our study primarily focused on mouse models, the relevance of the LGR5^+^ compartment deserves to be further investigated in human liver cancer. Conceivably, the role of these cells could be more prominent for a subset of patients with high levels of LGR5 expression, such as *CTNNB1*-mutated or alcohol-related HCC patients^25,26^. Although LGR5^+^-targeting therapies are still largely in their infancy, the analogy with neuroendocrine tumors, which are successfully combated by radioactive somatostatin analogs (e.g., ^177^Lu-Dotatate) that target receptors with homology to LGR5^27^, suggests that radioactive drugs (e.g., R-spondin) may be explored for developing CSC-targeted therapeutics against liver cancer^28^. Our results, however, indicate that such therapy may require combination with particular conventional anticancer therapies to enhance the efficacy.

Overexpression of LGR5 has been previously reported in patient HCC^17^, and we confirmed that this is more pronounced in *β-catenin*-mutated liver tumors. Although the elevation of LGR5 expression and potential association with clinical outcome have been observed in HCC patients, whether it can serve as an independent prognostic biomarker remains to be further investigated in specifically designed tumor marker prognostic studies in patients^29^.

Of note, these early observations are based on mRNA expression, due to the lack of a reliable anti-LGR5 antibody. We now used transgenic mice in which LGR5-expressing cells co-express GFP, and we can conditionally ablate these cells with the DT-DTR system^30^. This model allows for the identification and direct visualization of LGR5-expressing cells based on GFP expression, as well as isolation of LGR5–GFP^+^ cells for further functional analyses and detailed characterization.

In intestinal adenomas, LGR5 marks 5–10% of the cells, which keep fueling the growth of established mouse adenomas^13^. We found that the percentages of LGR5–GFP^+^ cells in murine liver tumors vary from 0.1% up to 55% (7.3 ± 1.8%, *n* = 55). Over 32% of the primary liver tumors harbor relatively high percentages (over 5%) (Supplementary Fig. 2c). In colon cancer, the percentage of LGR5-expressing cells has been reported to be associated with different background of the tumors, especially the accumulation of certain oncogenic mutations^8^. Thus, we speculate that the large variation of the abundance of LGR5 cells in murine liver tumors may also be related to different types of oncogenic mutations, although this hypothesis requires further investigation. Of note, DEN was used to induce primary liver cancer in this study, and this compound is associated with the accumulation of liver *β-catenin* mutations^31^. Therefore, it is possible that our results are mainly relevant to liver cancers with deregulated Wnt/β-catenin signaling, and their importance requires further investigation in other mutational backgrounds (e.g., deregulated TSC/mTOR signaling)^32^ as well.

Through a series of functional assays, in particular in vitro organoid initiation and in vivo tumor formation, we have demonstrated the importance of these LGR5 TICs in liver cancer. To define the potential for therapeutic targeting, we have performed LGR5 lineage ablation in organoids in vitro and in the tumor-bearing mouse model. Of note, the presence of LGR5 cells in tumors is likely dynamic. We observed large variations of their percentages among different primary murine liver tumors, and allograft tumors generally contain lower numbers of LGR5 cells (less than 1%). In colorectal cancer, advanced stages compared with the early stages contain less LGR5 cells^33^. A speculative explanation could be that the tumors are derived from LGR5-positive stem cells, yet these cells are suppressed thereafter during tumor progression^33^. The dynamics could be essential for determining at which stage to target LGR5 TICs. When we performed LGR5 cell ablation in established tumors, we only observed a minor effect, probably due to a low percentage of LGR5 cells as well as their dispensable function at that stage, while depletion at the early stage yielded optimal antitumor effects. This result is in line with previous findings showing that LGR5 cells play distinct roles in primary and metastatic colon cancer^8^. In addition, the percentage of LGR5-expressing cells in primary murine tumors seems not strictly correlated with the ability of initiating allograft (Fig. 3e). Because primary tumors were first cultured into tumor organoids, these organoids were engrafted into mice to initiate the allograft. Thus, this indirect assay may not fully recapitulate the initial status of the primary tumor.

Although we have demonstrated the feasibility and value of targeting LGR5 TICs in murine liver cancer, therapeutic ablation of these cells remains challenging. Resistance to conventional therapy is a common feature of CSCs^2^. We found that treatment with sorafenib or 5-FU enriches LGR5 cells, consistent with the findings in gastric^34^ and colorectal cancer^35^. Different mechanisms may contribute to treatment resistance. Although LGR5 stem cells are generally fast-cycling in the intestine^36^, the existence of quiescent LGR5 cells has been reported in basal cell carcinoma, which mediates relapse after treatment^12^. Cell plasticity could be one of the potential mechanisms of treatment resistance. The loss of LGR5 stem cells in the intestine can be compensated by transdifferentiation from other stem cell pools^30^, or through plasticity of their enterocyte-lineage daughters^37^. Cancer cell plasticity, shifting dynamically between a differentiated and a stemness state, has also been proposed as an important feature contributing to tumor progression, metastasis, and therapeutic response^38^. We have now observed the induction of LGR5^+^ from LGR5^−^ liver cancer cells. This may implicate cell plasticity of LGR5 CSCs, but there could also be other mechanisms regulating the origin and expansion of LGR5 cells, as for example, changes in the culture environment. Eventually, these LGR5 liver cancer cells may partially contribute to treatment resistance.

Currently, several innovative scenarios are being explored to therapeutically target CSCs, including antibody–drug conjugates^9^, targeting quiescent CSCs^39^, and inhibiting CSC-related pathways^2^. However, as discussed, different mechanisms could lead to treatment resistance^8^. Thus, combined therapies are likely necessary in this respect. With the intention to fully expand the stem cell pool, cetuximab has been used to first trigger the LGR5 population, followed by the ablation of these CSCs. This combined therapy has resulted in potent efficacy against colorectal cancer^10^. Similarly, we have observed that the combination of LGR5 lineage ablation with 5-FU chemotherapy can also lead to enhanced anti-liver cancer activity in mouse organoid. However, a combination of LGR5 lineage ablation with sorafenib did not yield enhanced antitumor activity. This is probably related to the mild effect of sorafenib in triggering the LGR5 CSC pool.

Last, a potential concern of such strategies is the possible harmful effects on normal LGR5 stem cells. In the intestine, colon, and skin, although LGR5 stem cells essentially contribute to tissue renewal at a daily basis^5,6^, their loss can be compensated by transdifferentiation from other reserve stem cell pools^30,40^ or through plasticity of their daughter cells^37^. Importantly, antibody-conjugated drug targeting LGR5 CSCs in colon cancer has no major impact on the function of normal LGR5 stem cells^9^. In the liver, LGR5 stem cells are absent during homeostasis, but only transiently activated upon injury likely without major contribution toward tissue repair^4,14^. Thus, we envision that our identification of targetable LGR5 TICs in murine liver cancer bears important implications for future therapeutic development.

## Methods

### Primary liver tumor model

*Lgr5–DTR–GFP* transgenic mice (kindly provided by Genentech) specifically co-express the diphtheria toxin (DT) receptor (DTR) and green florescent protein (GFP) under the control of the *Lgr5* promotor^30^. Thus, LGR5^+^ cells can be identified by GFP expression, and LGR5–GFP^+^ cells can be specifically depleted by DT administration. *Lgr5–DTR–GFP* transgenic mice (3–4 weeks) were administered with diethylnitrosamine (DEN) by intraperitoneal injection (Sigma-Aldrich, i.p., 100 mg/kg) weekly for 6–17 weeks^41^. DEN is used to induce liver tumor in *Lgr5–DTR–GFP* transgenic and wild-type mice, which could cause liver disease from basophilic foci, hyperplasic nodules, hepatocellular adenoma, and finally lead to HCC^31,42,43^. Mice were killed 3–16 months after the last DEN injection, and livers/tumors were collected for further experiments (Supplementary Data 1: in total, 41 mice were monitored; 80.5%, 33 out of 41, mice formed liver tumors; the expression of LGR5 in each tumor/tumor-surrounding tissues was also listed; Supplementary Fig. 2g, h). For each liver, biopsies were taken from the tumor and tumor- surrounding tissue. If livers contain more than one tumor, individual tumors were collected and analyzed separately. For CCl_4_-induced liver injury, *Lgr5–DTR–GFP* transgenic mice were weekly repeated administered with (6 or 17 weeks) intraperitoneal CCl_4_ injection (10 µl/20 g body weight of 10% CCl_4_ solution in corn oil or corn oil as control). All animal experiments were approved by the Committee on the Ethics of Animal Experiments of the Erasmus Medical Center.

### HCC specimens and patient information

HCC specimens (paired tumor tissue and adjacent tumor-free liver tissue) were collected from HCC patients undergoing tumor resection at the Erasmus Medical Center, The Netherlands. Samples were stored at −80 °C and then used for RNA extraction. In total, 74 specimens were obtained from HCC patients, and the corresponding clinicopathological data are summarized in Supplementary Table 2. HCC-specific survival was assessed in all patients and they were stratified according to relative *LGR5* expression (below and above median – 0.047). The Kaplan–Meier method was used to estimate survival outcome curves, and the log-rank test was used to compare the survival between the two groups. The hazard ratio (HR) for HCC-specific survival was estimated using the Cox proportional hazard regression model. The study was approved by the medical ethical committee of Erasmus Medical Center, and all the patients signed the informed consent before tissue donation. In addition, the study protocol conforms to the ethical guidelines of the 1975 Declaration of Helsinki.

### Online database

For analysis of *LGR5* mRNA expression, data were retrieved from three independent HCC cohorts, including The Cancer Genome Atlas (TCGA), Wurmbach^44^, and Roessler^45^. For survival analysis based on *LGR5* mRNA expression, the TCGA cohort was used. For analysis of the relationship between gene mutation and *LGR5* expression, three independent cohorts were investigated, including TCGA, International Cancer Genome Consortium-France (LICA-FR), and International Cancer Genome Consortium-Japan (LIRI-JP).

### Tumor organoid culture

Digestion solution II (37 °C, 30 min, 500 µg/ml of collagenase type XI, 200 µg/ml of Dnase-1, 1% FBS in DMEM medium) (collagenase type XI: Sigma-Aldrich; Dnase-1: Sigma-Aldrich) was used to digest liver or tumor tissues into single-cell suspension. Single-cell suspension was directly mixed with matrigel (Corning BV) and then used for culturing, or sorted for further experiments. Sorted cells were also mixed with matrigel and seeded for organoid initiation. Cells were cultured in organoid culture medium, which was based on advanced DMEM/F12 (Invitrogen), supplemented with 1% (vol/vol) of N2 (Invitrogen), 2% (vol/vol) of B27, 1.25 μM N-acetylcysteine (Sigma-Aldrich, antioxidant agent), 10 nM gastrin (Sigma-Aldrich), 50 ng/ml EGF (Peprotech, epidermal growth factor), 10% (vol/vol) of R-spondin-1 (conditioned medium produced by 293T-H-RspoI-Fc cell line, WNT/β-catenin signaling pathway activator), 100 ng/ml FGF10 (Peprotech, fibroblast growth factor 10), 10 mM nicotinamide (Sigma-Aldrich), and 50 ng/ml HGF (Peprotech, hepatocyte growth factor). For the first 8–12 days, organoids were supplemented with 10 μM Y-27632 (Sigma-Aldrich), Noggin, and Wnt3a-conditioned medium. The medium was refreshed every 2 days, and passage was performed in split ratios of 1:2–1:15 weekly. The proposed tumor organoid phenotypes are based on the expression of EpCAM/CK19 positive for CC-like and HNF4ɑ/AFP positive for HCC-like phenotype.

### Histology, immunohistochemistry, and immunofluorescence

The liver or tumor was fixed in 4% paraformaldehyde (PFA) overnight at 4 °C. For immunofluorescence, samples were further dehydrated with 30% sucrose (Sigma-Aldrich, 4 °C, overnight), stored at −80 °C, and then sectioned at 8 μm for further analysis. Images were acquired with a Zeiss LSM510META confocal microscope. For histology and immunohistochemistry, materials were dehydrated with 70% ethanol, embedded with paraffin, and sectioned at 4 μm for staining. Images were acquired with a Zeiss Axioskop 20 microscope. All antibodies are listed in Supplementary Table 3.

### Organoid-based allograft tumor model

Cold advanced DMEM/F12 medium was used to collect the organoids. After centrifuging, the supernatant was discarded and organoid pellets (organoid fragmentation size: range from 5 to 150 µm) were mixed directly with matrigel in a ratio of 1:1 with a total volume of 200 µl. In total, 4–6-week-old female NOD.Cg-PrkdcSCIDIl2rgtm1Wjl/SzJ (NSG) mice, NOG/JicTac (CIEA NOD.Cg-Prkdc-scid Il2rg-tm1Sug) mice, or nude mice (NMRI:BomTac-Nude) were purchased from Taconic, and subcutaneously injected with the collected tumor organoids. The characterization of phenotypes for murine allograft tumor is based on the expression of EpCAM/CK19 for CC-like and HNF4ɑ/AFP for HCC-like phenotype (Supplementary Data 2). Tumor dimensions were measured using calipers, and tumor volume was calculated as 0.523 × length × width × width^9^. Tumor formation was monitored every other day, and mice were killed to harvest tumors after the tumor diameter reached ~2 cm. Tumor tissues were stored or cultured as described above.

### Cell ablation by DT and treatment of 5-FU/sorafenib

To ablate LGR5^+^ cells in organoids, DT (Calbiochem, 1–10 ng/ml) was added to organoid expansion/initiation medium, followed by further analysis^14^. For in vivo ablation, DT was administered via intraperitoneal injection every other day (50 µg per kg body weight). If mice suffering from weight loss ≥ 10%, compared with the previous injection, the injection was omitted. 5-FU/sorafenib were also administered via intraperitoneal injection every other day (5-FU/sorafenib: 30 mg per kg body weight) (sorafenib: Bio-Connect BV; 5-FU: Sigma-Aldrich).

### qRT-PCR

For freshly FACS-sorted cells and HCC specimens, RNeasy Micro Kit (QIAGEN) was used to isolate RNA. For organoids, Machery-NucleoSpin RNA II kit (Bioké) was used. Quantification was measured with Nanodrop ND-1000 (Wilmington). RNA was then converted to cDNA by using a cDNA Synthesis kit (TAKARA BIO INC.). Real-time PCR reactions were performed with SYBRGreen-based real-time PCR (Applied Biosystems^®^) and amplified in a thermal cycler (GeneAmp PCR System 9700). For cells collected from murine tissues, glyceraldehyde 3-phosphate dehydrogenase (*Gapdh*) gene was used as reference. For quantification of *LGR5* mRNA in human tumors and tumor-free liver tissues, *Gusb* (beta-glucuronidases), *Hprt1* (hypoxanthine phosphoribosyltransferase 1), and *Pmm1* (phosphomannomutase 1) were used as reference genes. All primers are listed in Supplementary Table 4.

### RNA sequencing

The total RNA was isolated using RNeasy Micro Kit (QIAGEN). The quantity of RNA was measured by a NanoDrop 2000. The collected RNA was further amplified by using SMARTer kit. Then, RNA sequencing was performed by Novogene with the paired-end 150-bp (PE 150) sequencing strategy. Gene expression was analyzed. The identification of differentially expressed genes is based on *P* < 0.05 and absolute values of logFc > 1.5. GSEA with the library of Wiki2019 was performed to reveal the alteration of signaling pathways.

### FACS analysis

For FACS analysis, single cells derived from liver tumors/tumor-surrounding tissues or organoids were suspended in DMEM plus 2% FBS. Cell suspensions were analyzed using a BD FACSCalibur or BD FACSAria^TM^ II. For FACS sorting, a BD FACSAria^TM^ II cell sorter was used to isolate the target cell population. Propidium iodide (PI) staining was performed to exclude dead cells, and CD45 staining was adopted for excluding leucocytes.

### Metabolic activity analysis of organoids

Different organoid lines were seeded separately in a 24-/48-well plate. Sorafenib (10 µM) or 5-FU (10 µM) was added to the organoid culture since the initial day or post initiation day 3, respectively. Drugs were refreshed every other day. At days 6–7, organoids were incubated with Alamar Blue (Invitrogen, 1:20 in DMEM) for 4 h (37 °C), and then the medium was collected for analysis of the metabolic activity of the cells. Absorbance was determined by using fluorescence plate reader (CytoFluor^®^ Series 4000, Perseptive Biosystems) at the excitation of 530/25 nm and emission of 590/35. Each treatment condition was repeated four times and matrigel with medium only was used as a blank control.

### Statistical analysis

Prism software (GraphPad Software) was used for all statistical analyses. For statistical significance of the differences between the means of groups, we used Mann–Whitney *U* test; for statistical significance of the differences between groups with inequivalent sample sizes, we used Welch test; for statistical significance of the differences between paired samples, we used paired *T* test; for statistical significance of the differences between multiple independent groups, we used two-way ANOVA (except for Mann–Whitney *U* test, the use of other statistical methods is indicated in the legends). Differences were considered significant at a *P* value less than 0.05.

### Reporting summary

Further information on research design is available in the Nature Research Reporting Summary linked to this article.

## Supplementary information

Suppmentary Information

Peer Review File

Description of Additional Supplementary Information

Supplementary Data 1

Supplementary Data 2

Supplementary Data 3

Supplementary Data 4

Supplementary Data 5

Supplementary Data 6

Reporting summary

## Data Availability

The RNA-seq data have been deposited in the GEO database under the accession code GSE137517. The human *LGR5* expression and HCC mutation data referenced during the study are available in a public repository from the TCGA and LICA databases. The source data underlying Figs. 1c-d, 2, 3e, 4d, f, h, i, p, 5b, f, h-i, 6c, h, I, 7b, c, f, g, 8b, c, f, g and Supplementary Figs. 2a, g, 3, 4, 6b-c, 7b-e, 8a-b, 9, 10c-d, 11, 12a-b and 13g-h are provided as a Source Data file. All the other data supporting the findings of this study are available within the article and its supplementary information files and from the corresponding author upon reasonable request. A reporting summary for this article is available as a Supplementary Information file.

## Source Data

Source Data
