# Supplementary material for: LGR5 marks targetable tumor-initiating cells in mouse liver cancer
Source: Nat Commun. 2020 Apr 23;11:1961. doi: 10.1038/s41467-020-15846-0 (PMC7181628; doi:10.1038/s41467-020-15846-0)
Supplement: Supplementary file 1 — Suppmentary Information [file 41467_2020_15846_MOESM1_ESM.pdf]

Supplementary Information for:

LGR5 marks targetable tumor-initiating cells in mouse liver cancer

Cao et al

Table of contents

Supplementary Figure 1

Supplementary Figure 2

Supplementary Figure 3

Supplementary Figure 4

Supplementary Figure 5

Supplementary Figure 6

Supplementary Figure 7

Supplementary Figure 8

Supplementary Figure 9

Supplementary Figure 10

Supplementary Figure 11

Supplementary Figure 12

Supplementary Figure 13

Supplementary Figure 14

Supplementary Table 1

Supplementary Table 2

Supplementary Table 3

Supplementary Table 4

Supplementary Note

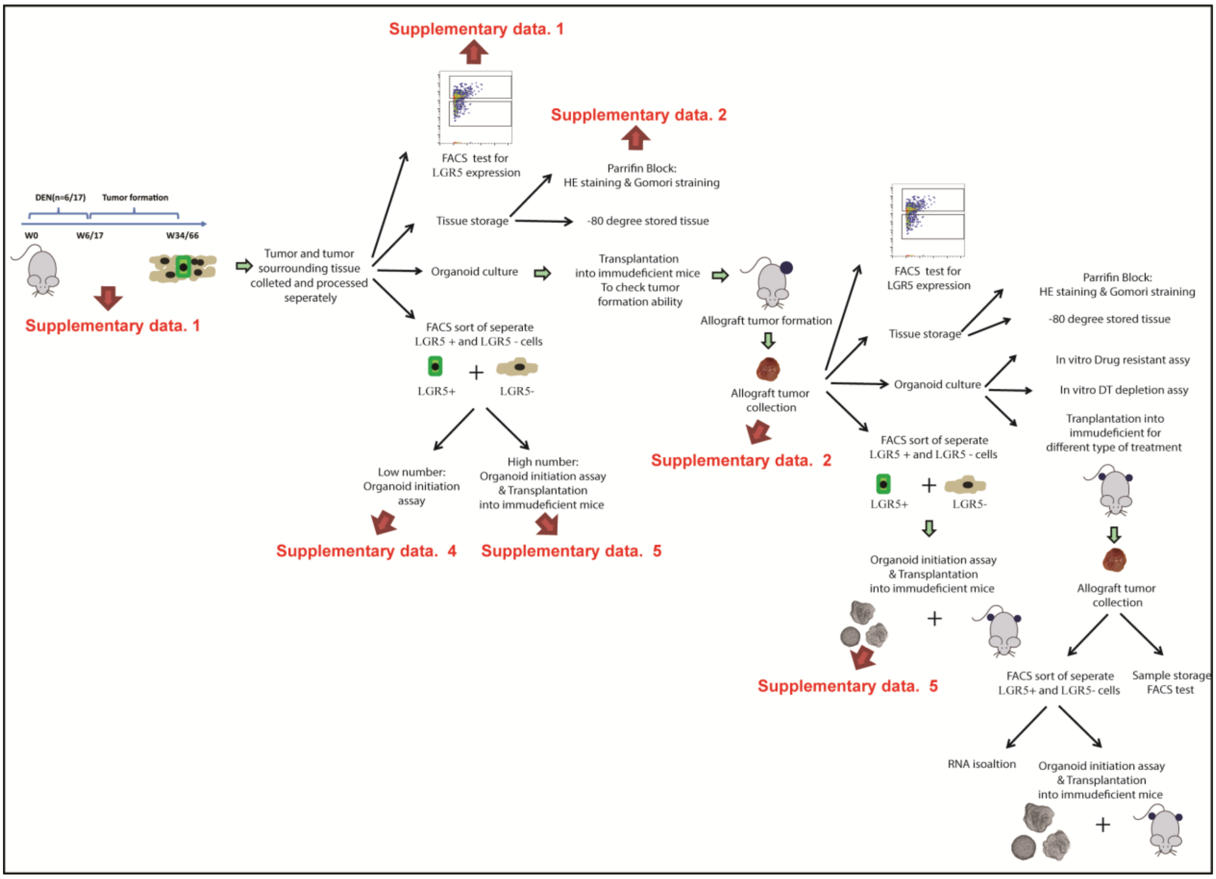

Supplementary Figure 1 | General flowchart of the experimental design.

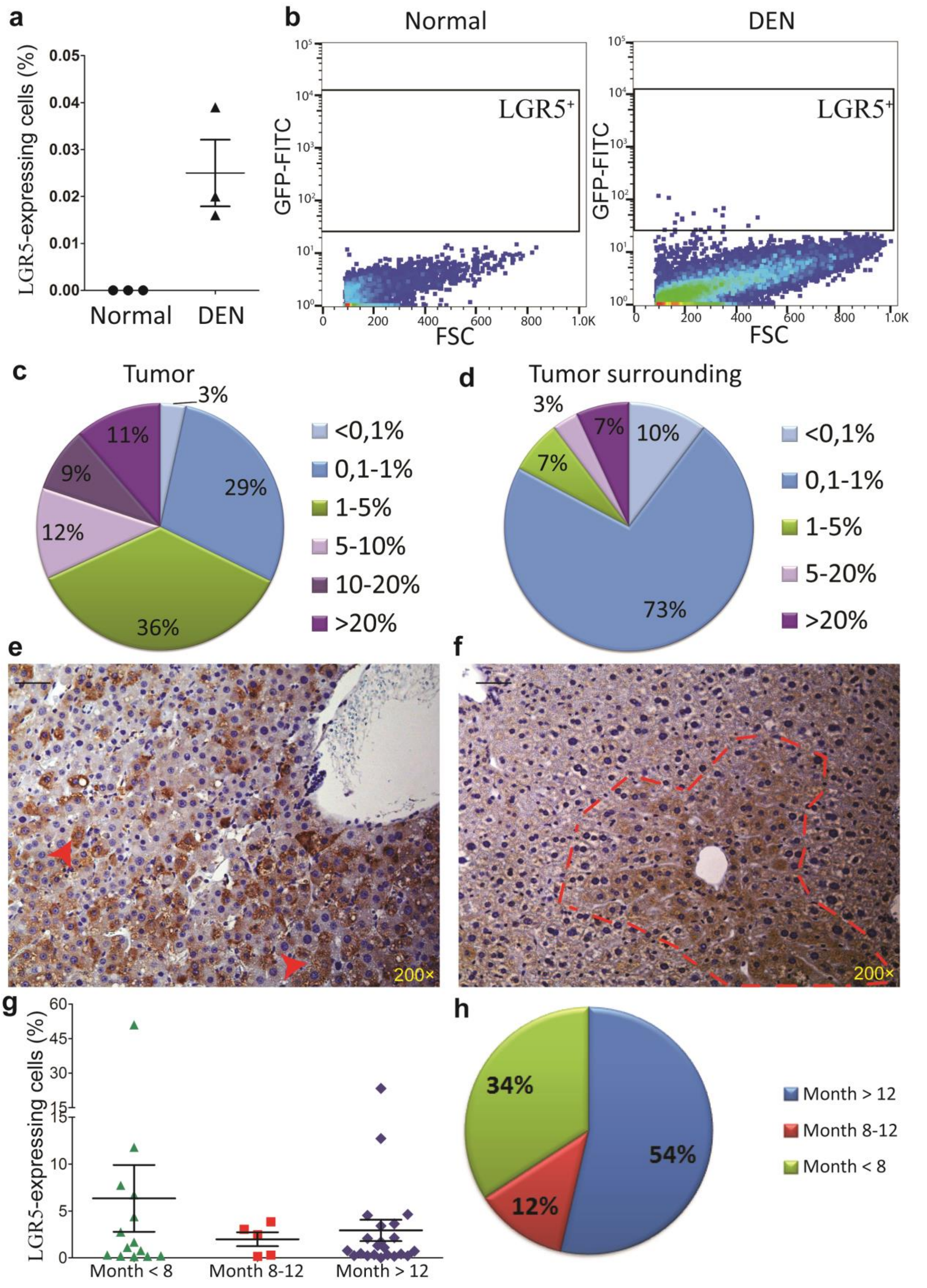

Supplementary Figure 2 | LGR5 expressing cells are present in the DEN-induced primary liver tumor.

a, LGR5^{+} cells are present in the liver upon DEN administration (Post DEN induction day 7). **b**,

Representative FACS plots showing that LGR5$^+$ cells are present in the liver following DEN administration (Post DEN induction day 7). **c**, Relative LGR5$^+$ fraction in DEN-induced liver tumors. **d**, Relative LGR5$^+$ fraction in tissue bordering the DEN-induced liver tumors (denominated as surrounding). **e-f**, Representative immunohistochemistry pictures showing the microscopic aspect of the LGR5$^+$ cells in liver cancer. Two types of LGR5$^+$ cell distribution patterns are apparent, scattered LGR5$^+$ cell distribution (**e**) and LGR5$^+$ island-like clusters (**f**). The pictures show an anti-GFP immunohistochemistry staining in which LGR5$^+$ cells are brown and nuclei are blue. Scale bar = 50µm**.** **g,** The percentage of LGR5-expressing cells within each tissue, grouped by tumor collection month (the month is counted since the first administration of DEN). Mean ± SEM. (**h**) Relative mice number fraction in all DEN-induced mice, grouped by tumor collection month. Source data are provided as a Source Data file.

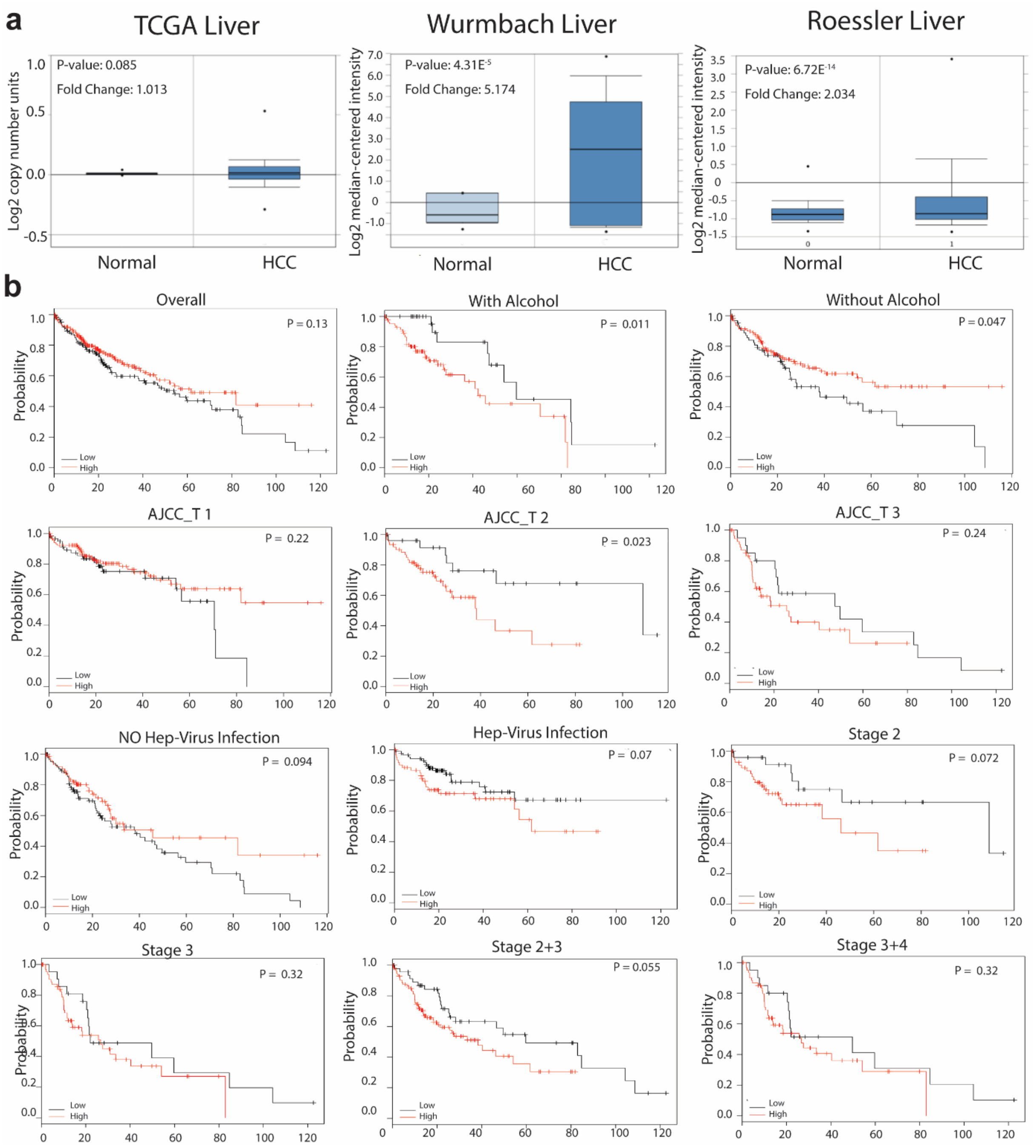

Supplementary Figure 3 | *LGR5* expression in HCC tissues and relation to patient outcome. a, The upregulated expression of *LGR5* in HCC tissues compared with normal tissues from TCGA Liver cohort (Normal vs. HCC: *n* = 115 vs. *n* = 97, *P* = 0.085, Fold change: 1.013), Wurmbach liver cohort (Normal vs. HCC: *n* = 10 vs. *n* = 35, *P* = 4.31E^{-5}, Fold change: 5.174) and Roessler liver cohort (Normal vs. HCC: *n* = 200 vs. *n* = 225, *P* = 6.72E-14, Fold change: 2.034). **b**, Kaplan-Meier curve for HCC of patients based on different etiology/tumor stage of HCC from TCGA Liver cohort. AJCC_T: American Joint Committee on Cancer (AJCC) tumor staging system; Stage: Barcelona Clinic Liver Cancer (BCLC) tumor staging system. Source data are provided as a Source Data file.

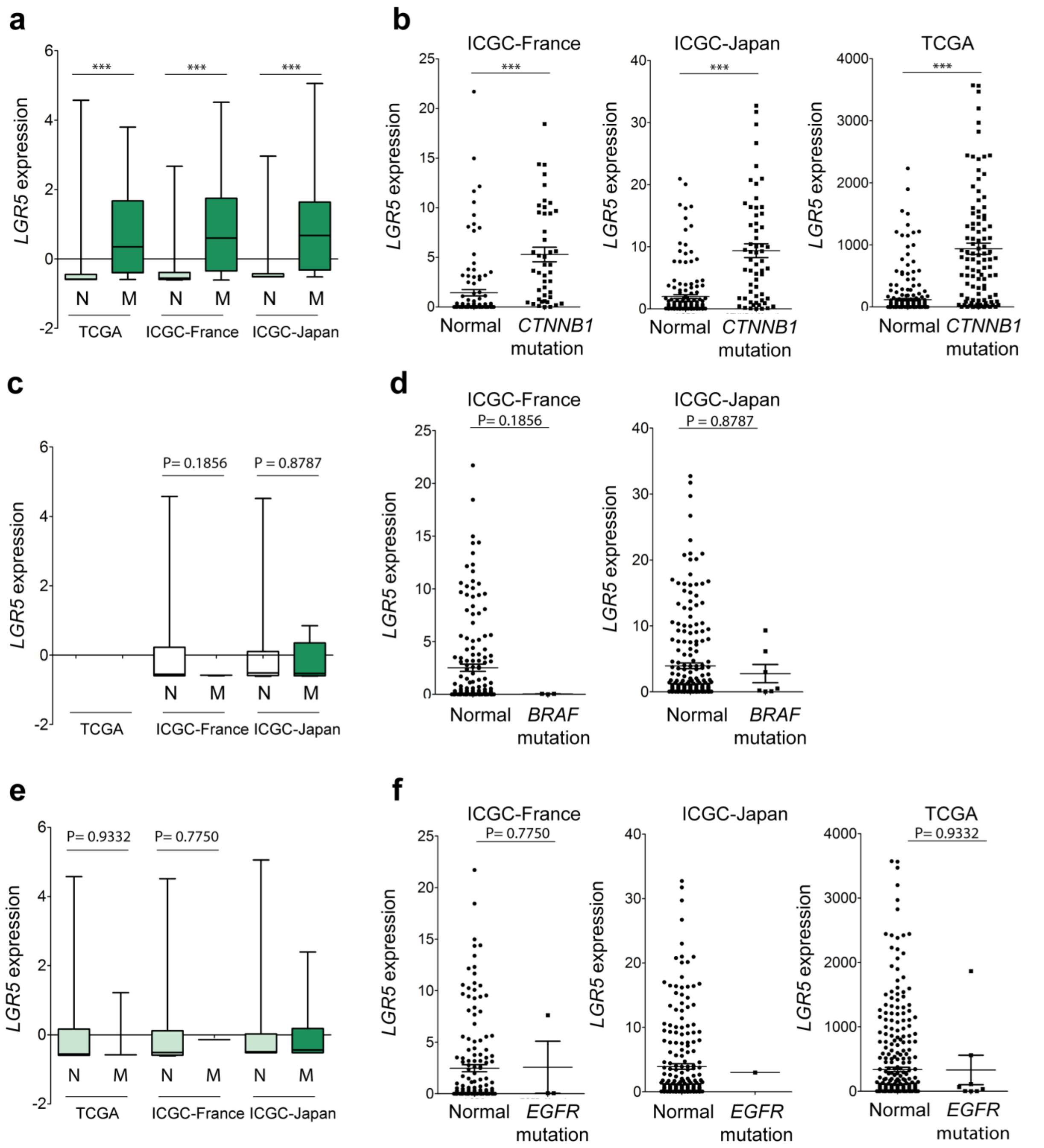

Supplementary Figure 4 | The expression level of *LGR5* is upregulated in *Ctnnb1* mutated HCC tumors. **a-b,** Upregulation of *LGR5* expression in *Ctnnb1* mutated compared to non-*Ctnnb1* mutated patient HCC tumors, in ICGC-France, ICGC-Japan and TCGA cohort. **c-d,** No significant difference of *LGR5* expression in *Braf* mutated compared to non-*Braf* mutated HCC tumors, in ICGC-France and ICGC-Japan cohort**. e-f,** No significant difference of *LGR5* expression in *Egfr* mutated compared to non-*Egfr* mutated HCC tumors, in ICGC-France, ICGC-Japan and TCGA cohort. Mean ± SEM. Source data are provided as a Source Data file.

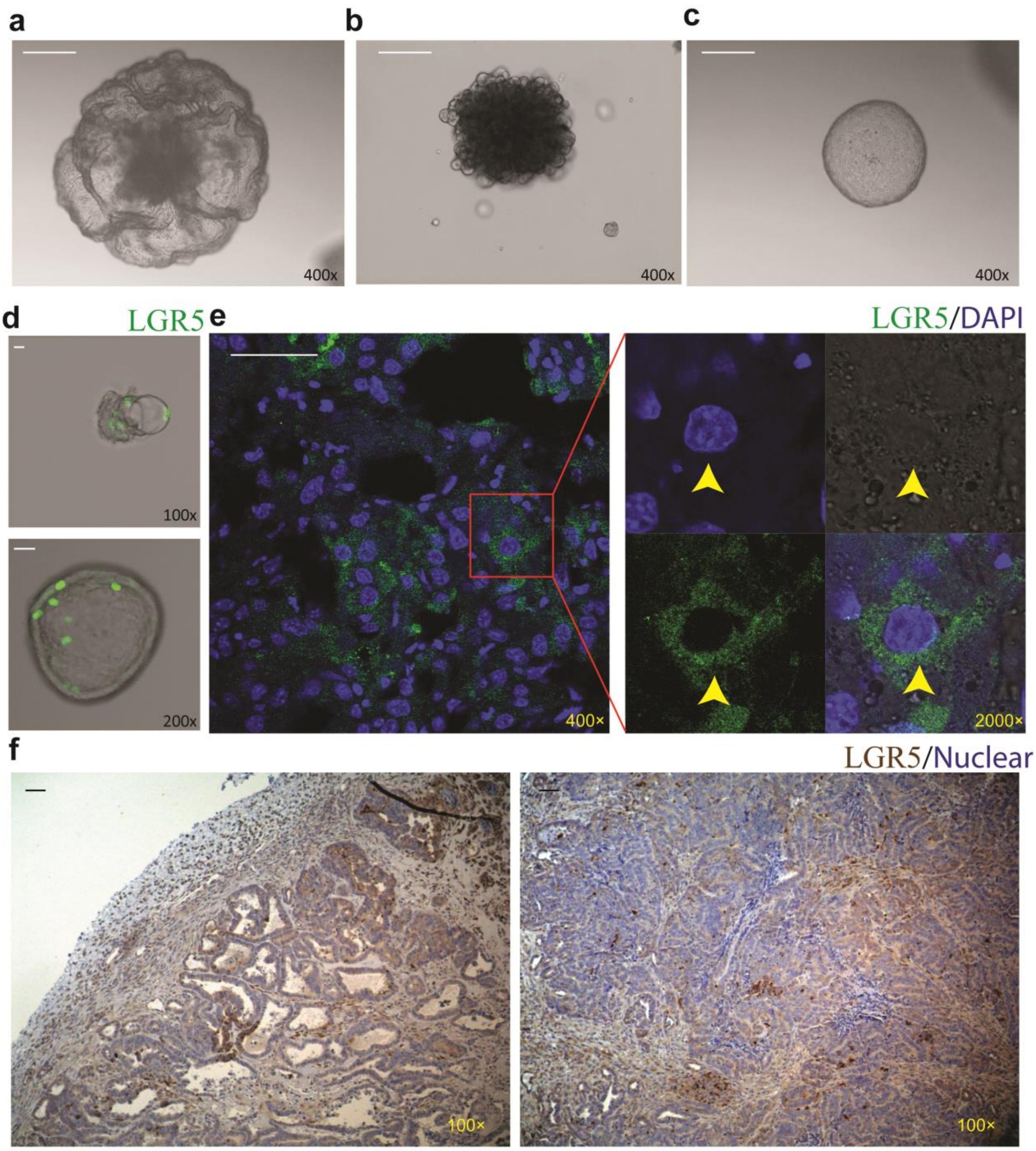

Supplementary Figure 5 | Allograft liver tumors maintain an $LGR5^+$ compartment. a-c, 3-7 days after initiation of allograft tumor-derived cultures, small organoids were observed and subsequently passage was performed every 4-7 days, employing splitting ratios ranging from 1:4 to 1:10 as appropriate. Representative pictures show the different morphologies of the tumor organoids obtained, which include but also flower-like, irregular sheet-like structures (**a**), and grape-like, condensed phenotypes (**b**), and. relative normal hollow sphere-like aspects (**c**). **d**, Representative confocal pictures showing the maintenance of an $LGR5^+$ compartment in tumor organoids (LGR5-driven GFP: green). **e**, Representative immunofluorescent pictures showing an $LGR5^+$ compartment

in allograft tumors (*LGR5*-driven GFP: green; DAPI: blue). **f**, Representative immunohistochemistry pictures showing expression of *LGR5* promotor-driven GFP in allograft tumors (anti-GFP immunohistochemistry: brown; nuclei: blue). Scale bar = 50µm.

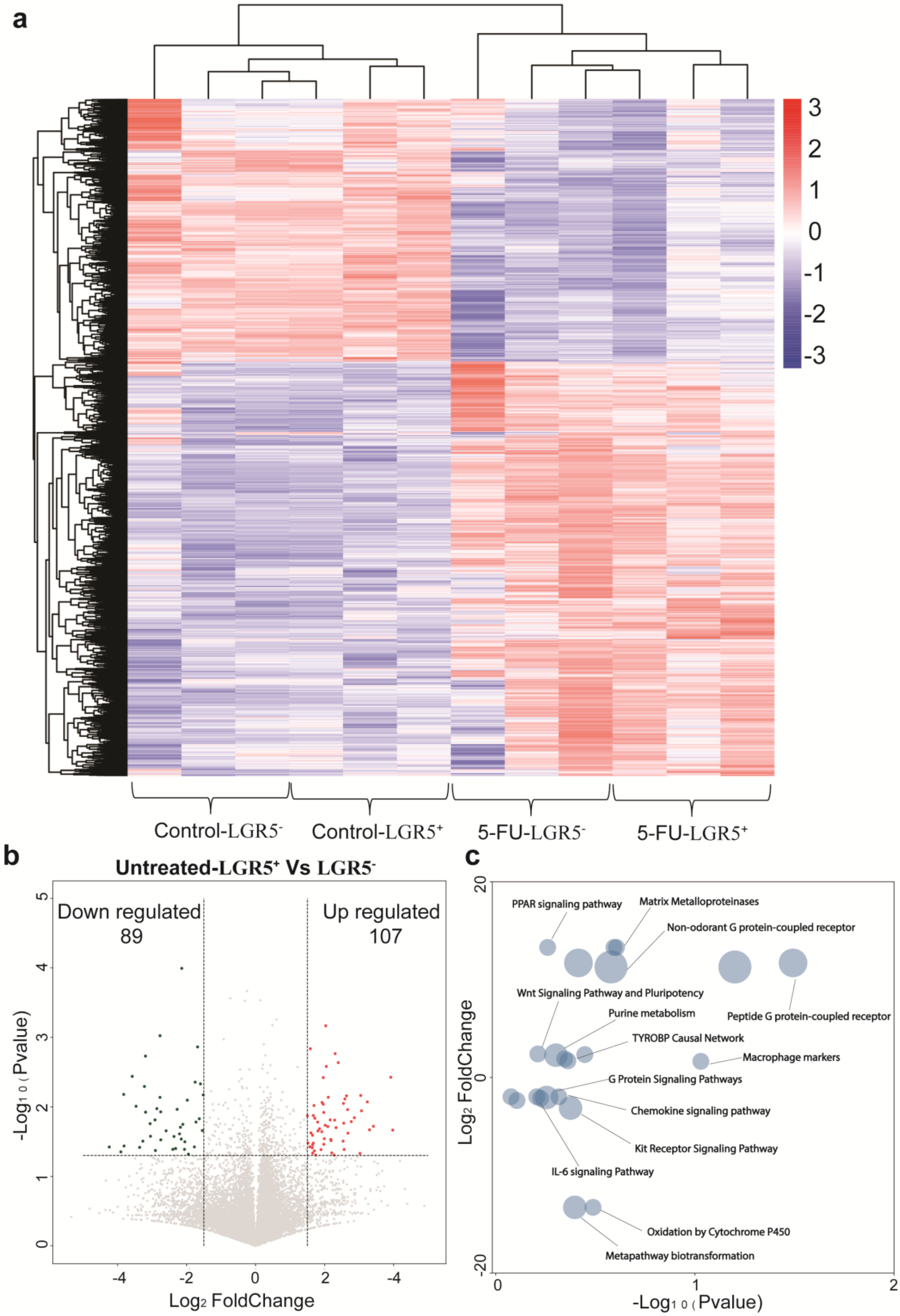
a
3
2
1
0
-1
-2
-3
Control-LGR5-
Control-LGR5+
5-FU-LGR5-
5-FU-LGR5+
b
Untreated-LGR5+ Vs LGR5-
Down regulated
89
Up regulated
107
-Log10 (Pvalue)
Log2 FoldChange
c
PPAR signaling pathway
Matrix Metalloproteinases
Non-odorant G protein-coupled receptor
Wnt Signaling Pathway and Pluripotency
Purine metabolism
Peptide G protein-coupled receptor
TYROBP Causal Network
Macrophage markers
G Protein Signaling Pathways
Chemokine signaling pathway
Kit Receptor Signaling Pathway
IL-6 signaling Pathway
Oxidation by Cytochrome P450
Metapathway biotransformation
Log2 FoldChange
-Log10 (Pvalue)

74

Supplementary Figure 6 | Genome-wide transcriptomic analysis of LGR5^{+} and LGR5^{-} cells by RNA-Seq. a, Hierarchical clustering showed a separation of all four different groups (Untreated LGR5^{+} cells, Untreated LGR5^{-} cells, 5-FU-treated LGR5^{+} cells, 5-FU-treated LGR5^{-} cells). **b**, A Volcano plot showing the most significantly differentially expressed genes between untreated LGR5^{+} and LGR5^{-} cells. **c**, GSEA with the library of Wiki2019 was performed to reveal the alteration of signaling pathways between untreated LGR5^{+} and LGR5^{-} cells. Source data are provided as a Source Data file.

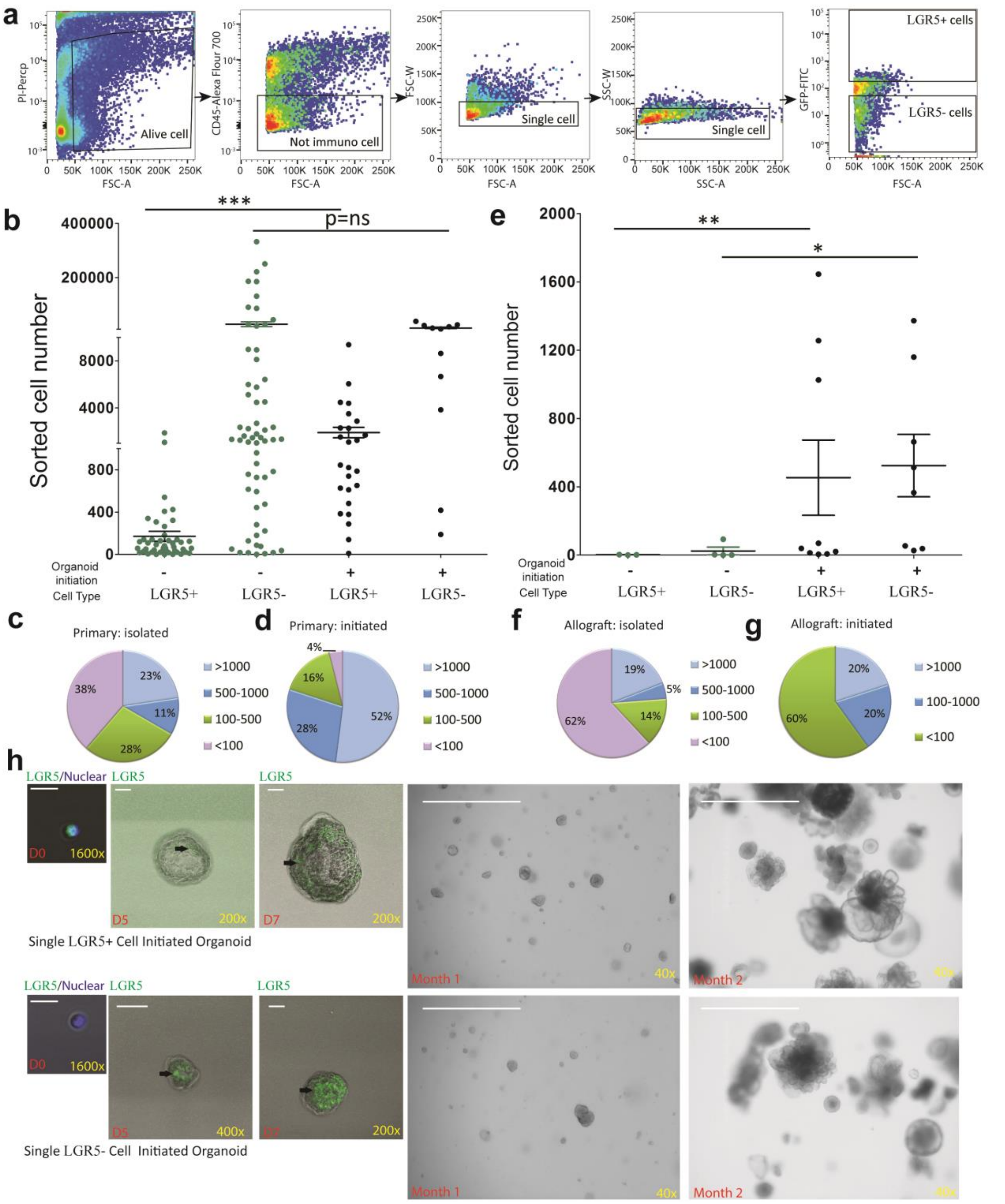

Supplementary Figure 7 | LGR5$^+$ cells have stronger organoid initiation ability. a, The FACS test/sort strategy for isolating LGR5-GFP$^+$ and LGR5-GFP$^-$ cells obtained from liver/allograft tumors/organoids. **b**, The numbers of sorted LGR5-GFP$^+$ and LGR5-GFP$^-$ cells yielding successful (black dots) or failure of organoid initiation (green dots), employing material obtained from DEN-induced murine livers (LGR5$^+$-non-initiation vs. LGR5$^-$-non-initiation vs. LGR5$^+$-initiation vs. LGR5$^-$-initiation: 171.4 ± 47.1, n = 46 vs. 28350 ± 8914, n = 60 vs. 1906 ± 441.6, n = 25 vs. 13860 ± 3654, n = 11, Mean ± SEM). c, The

distribution showing the percentage of the number of $LGR5^+$ cells which were isolated from primary liver tumors. d, The cell number distribution for the sorted $LGR5^+$ cells which can initiate organoid, from primary tumors. e, The exact sorted numbers for $LGR5^+$ cells and $LGR5^-$ cells , for organoid initiated (black dots) and non-organoid initiated (green dots), from allograft tumors ($LGR5^+$-non-initiated vs. $LGR5^-$-non-initiated vs. $LGR5^+$-initiated vs. $LGR5^-$-initiated: 2.0 ± 0.6, n = 3 vs. 24.3 ± 22.9, n = 4 vs. 453.7 ± 220.3, n = 9 vs. 524.3 ± 182.9, n = 8). **f**, Frequency distribution of the relative number of $LGR5^+$ cells obtained from allograft liver tumors. **g**, Frequency distribution of the number of $LGR5^+$ cells that display successful organoid initiation from material obtained from allograft tumors. **h**, Representative pictures tracing organoids initiation and growth from $LGR5^+$ or $LGR5^-$ cells. Black arrow: LGR5 expressing cells. Day0/5/7: Scale bar = 50μm; Month1/2: Scale bar = 1000μm. Source data are provided as a Source Data file.

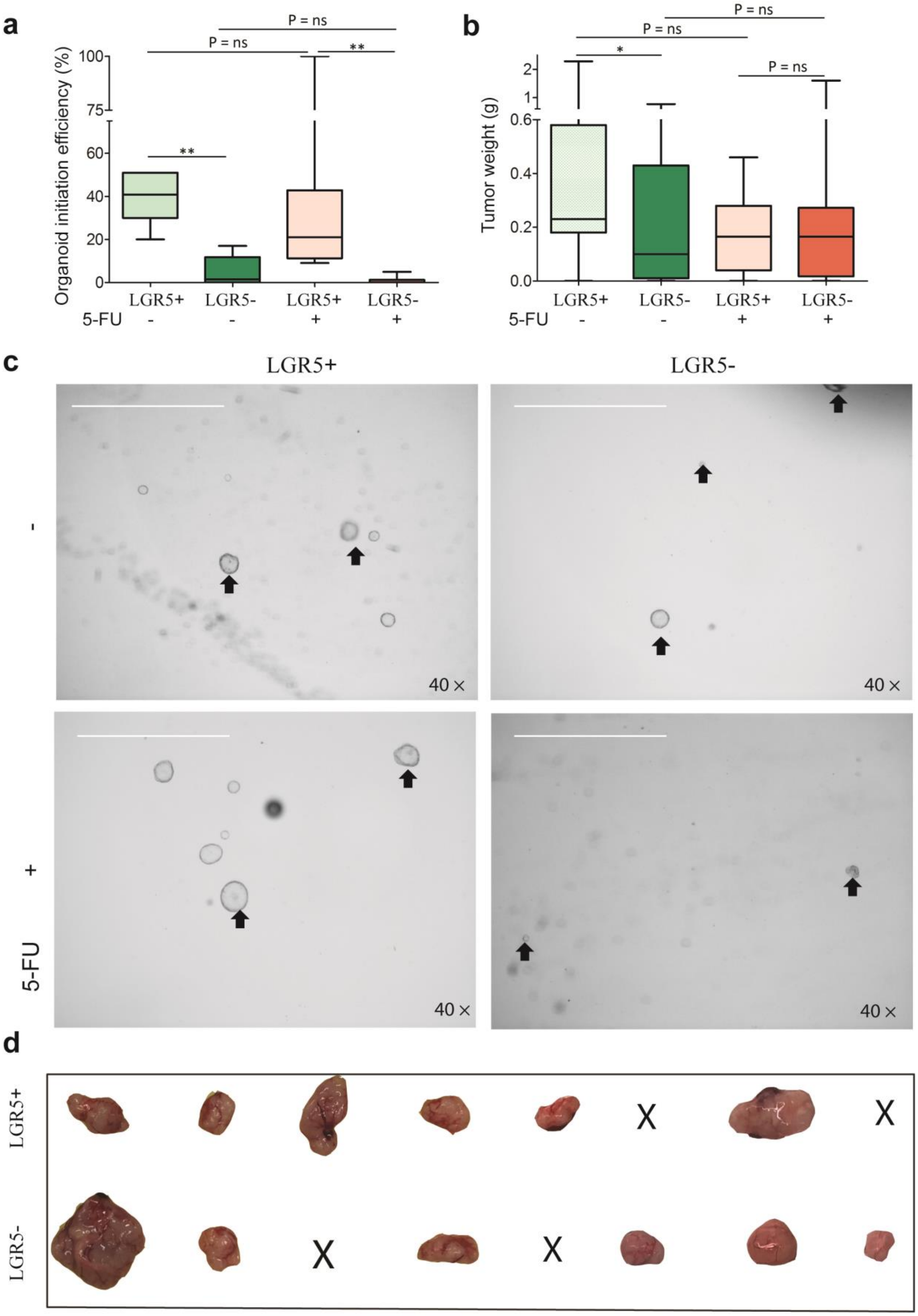
a
b
c
d
P = ns
**
*
Organoid initiation efficiency (%)
100
75
60
40
20
0
LGR5+
LGR5-
5-FU
-
+
Tumor weight (g)
2
1
0.6
0.4
0.2
0.0
40 ×

Supplementary Figure 8 | $LGR5^+$ cells from 5-FU treated tumors can initiate organoid and tumor. a, The organoid initiation ability of untreated $LGR5^+$ cells (39.5 ± 4.8 %, *n* = 6, Mean ± SEM), untreated $LGR5^-$ cells (5.0 ± 2.9 %, *n* = 6), 5-FU-treated $LGR5^+$ cells (31.2 ± 14.0 %, *n* = 6) and 5-FU-treated $LGR5^-$ cells (0.8 ± 0.8 %, *n* = 6). **b,** The tumor initiation ability of untreated $LGR5^+$ cells (0.50 ± 0.15 g, *n* = 15), untreated $LGR5^-$ cells (0.21 ± 0.06 g, *n* = 15), 5-FU-treated $LGR5^+$ cells (0.18 ± 0.05 g, *n* = 8) and 5-FU-treated $LGR5^-$ cells (0.31 ± 0.19 g, *n* = 8). **c**, Representative picture showing the organoid initiation ability of above four groups. Black arrow: initiated organoids. Scale bar = 1000μm. **d**, Pictures showing 5-FU-treated $LGR5^+$ cells and 5-FU-treated $LGR5^-$ cells initiated tumors. Source data are provided as a Source Data file.

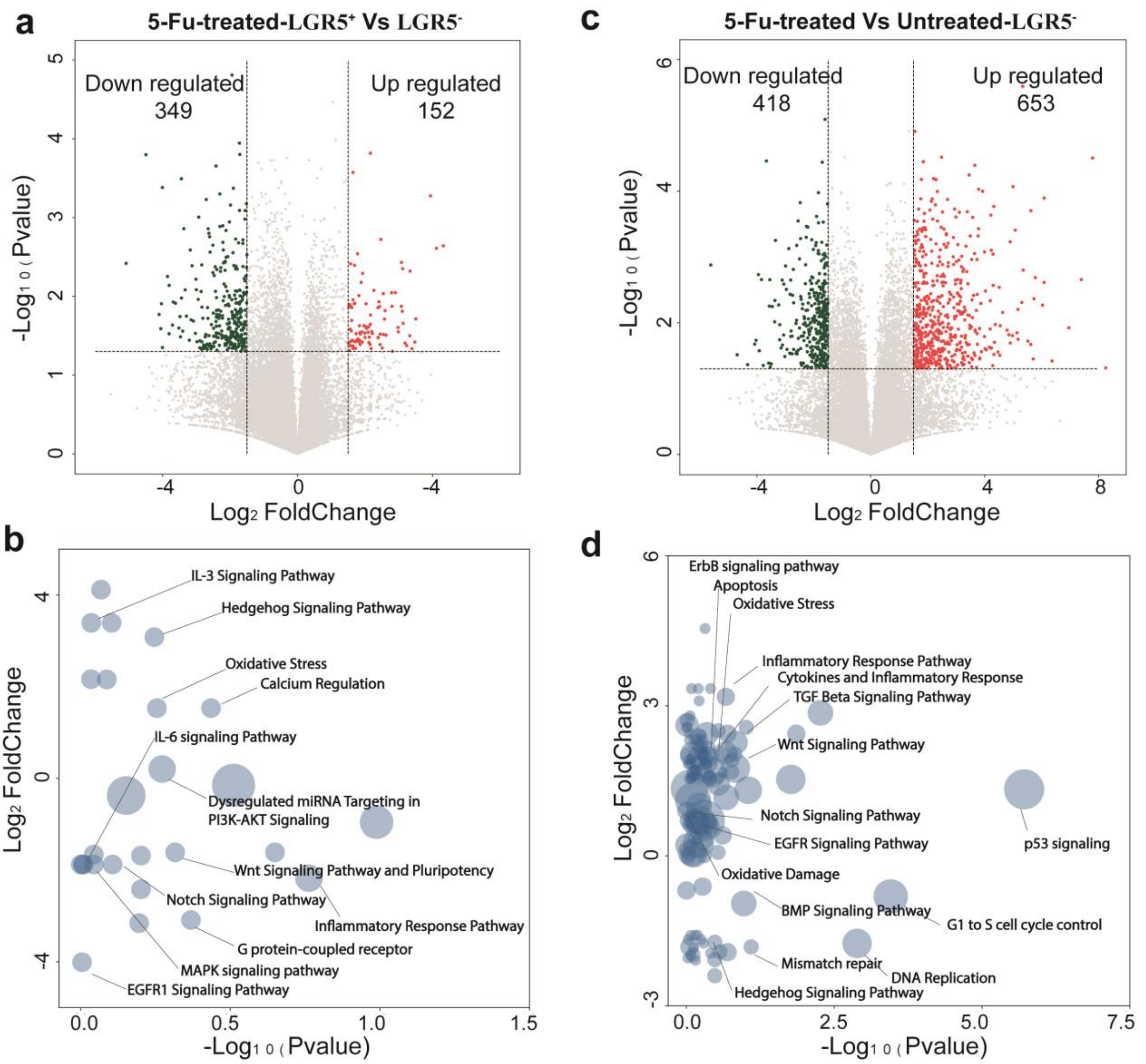

Supplementary Figure 9 | Genome-wide transcriptomic analysis of 5-FU treatment on LGR5$^{+/-}$ cells. **a**, A Volcano plot showing the most significantly differentially expressed genes between 5-FU treated LGR5^{+} cell/LGR5^{-} cells. **b**, Gene enrichment analysis (Wiki2019) of the differentially expressed genes between 5-FU treated LGR5^{+} cell/LGR5^{-} cells. **c**, A Volcano plot showing the most significantly differentially expressed genes between 5-FU treated/untreated LGR5^{-} cells. **d**, Gene enrichment analysis (Wiki2019) of the differentially expressed genes between 5-FU treated/untreated LGR5^{-} cells. Source data are provided as a Source Data file.

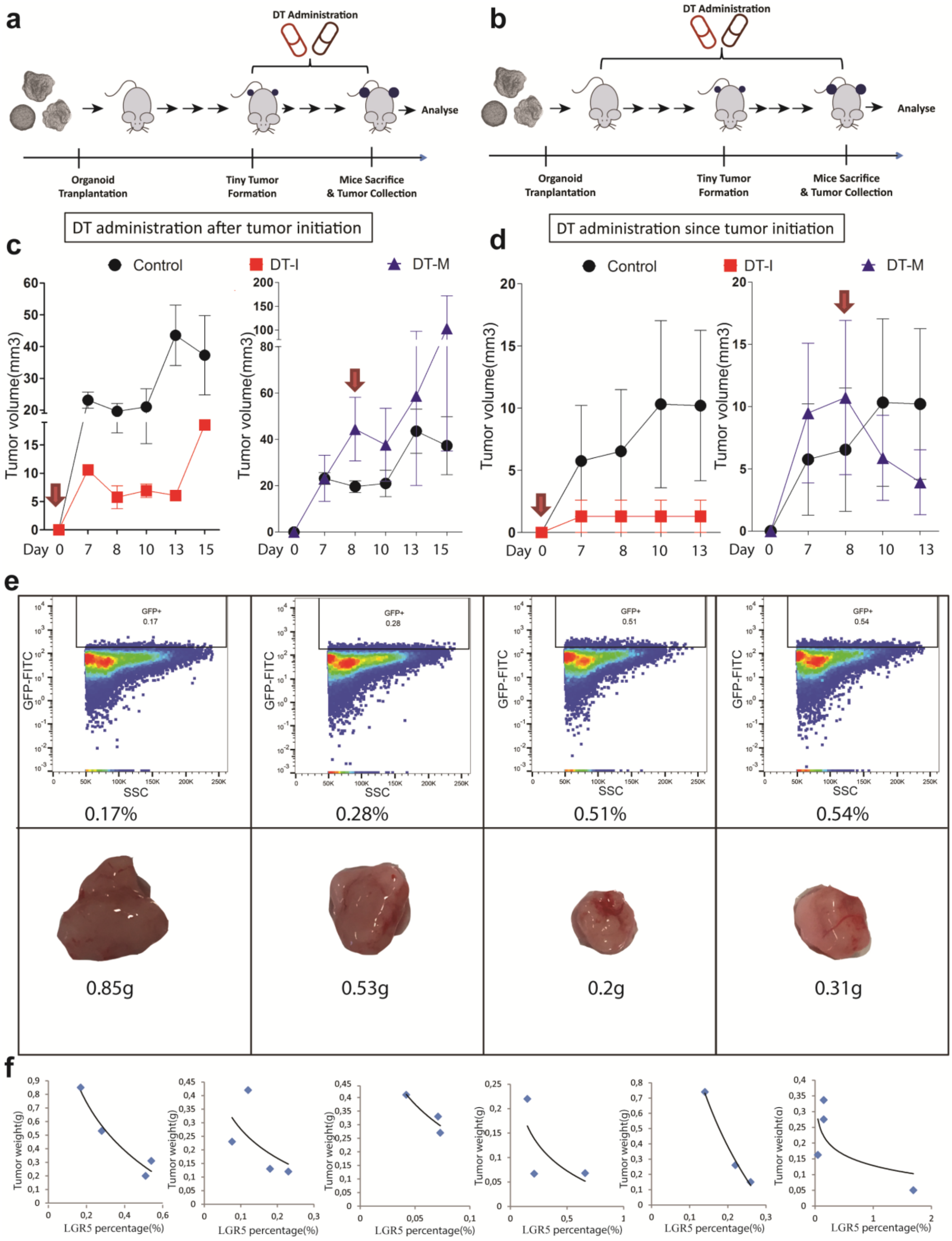

Supplementary Figure 10 | Targeting LGR5 impedes the tumor growth. a-b, Outline of the experimental strategies used to test the effect of DT administration during tumor growth (**a**) and at tumor initiation (**b**). **c-d**, Growth curves showing the effects of DT administration during the entire experimental period (Left, DT-I) and following DT intervention during tumor growth (Right, DT-M) for organoid strain 1 (**c**) and strain 2 (**d**). Mean ± SEM. Red arrow: onset of DT administration (n = 4 for

each time point). **e**, Representative FACS pictures (upper channel, with LGR5-GFP$^+$ expression) and tumor pictures (lower channel, with tumor weights) showing that the same tumor strains, collected from a single mouse, shows variable LGR5 expression. **f**, tumors collected from six individual mice (transplanted with same strain and same amount of organoid; collected on the same day, non-treated tumors) showing that smaller tumors have relatively higher LGR5 expression. Source data are provided as a Source Data file.

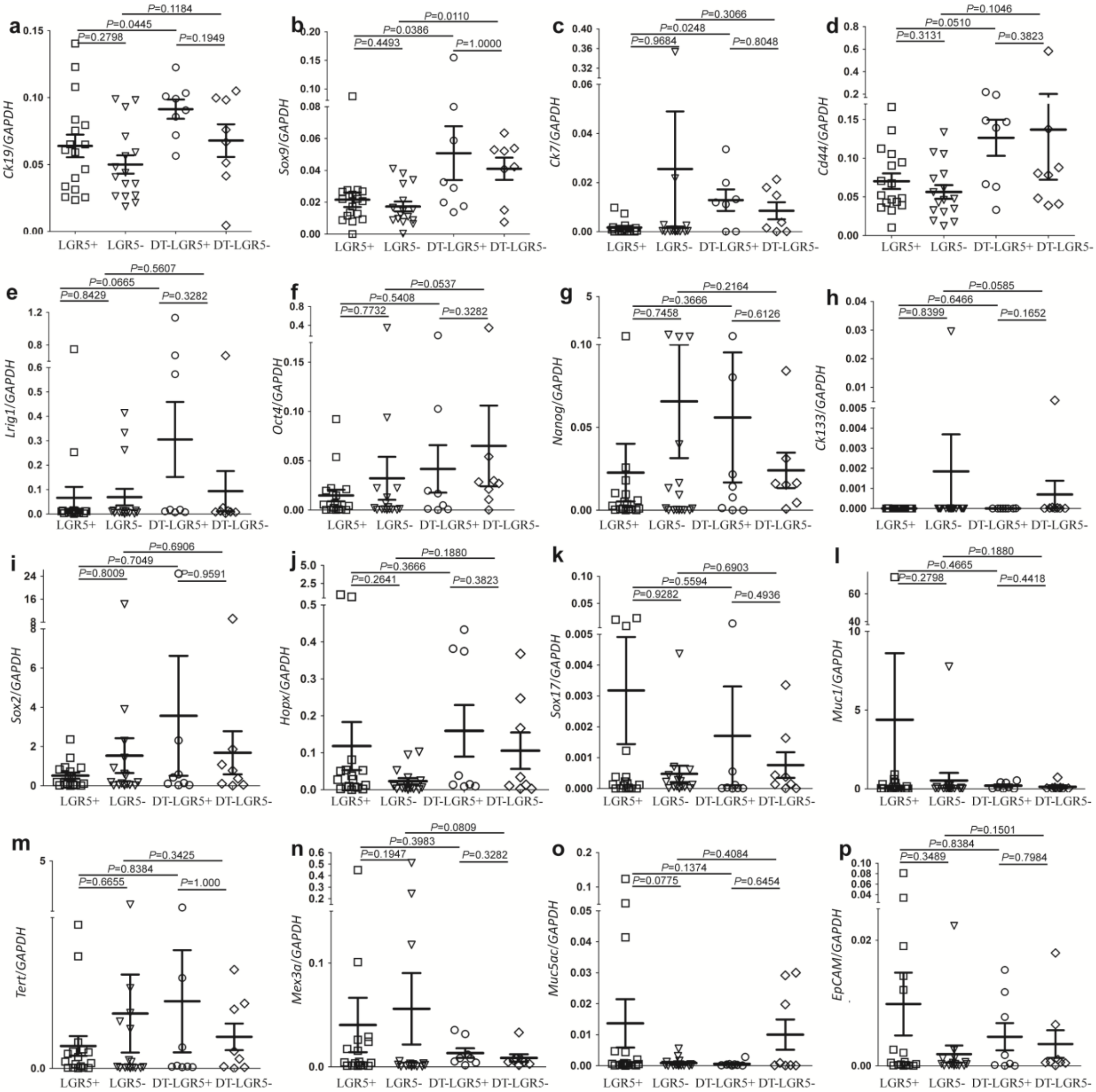

Supplementary Figure 11 | The expression profile of untreated/DT-treated $LGR5^+$ and $LGR5^-$ cells. **a-p,** The expression of stem cell/tumor stem cell markers in $LGR5^+$/$LGR5^-$ and DT treated tumor isolated $LGR5^+$/$LGR5^-$ cells was analyzed using qRT-PCR and related to a reference gene. Mean ± SEM. Source data are provided as a Source Data file.

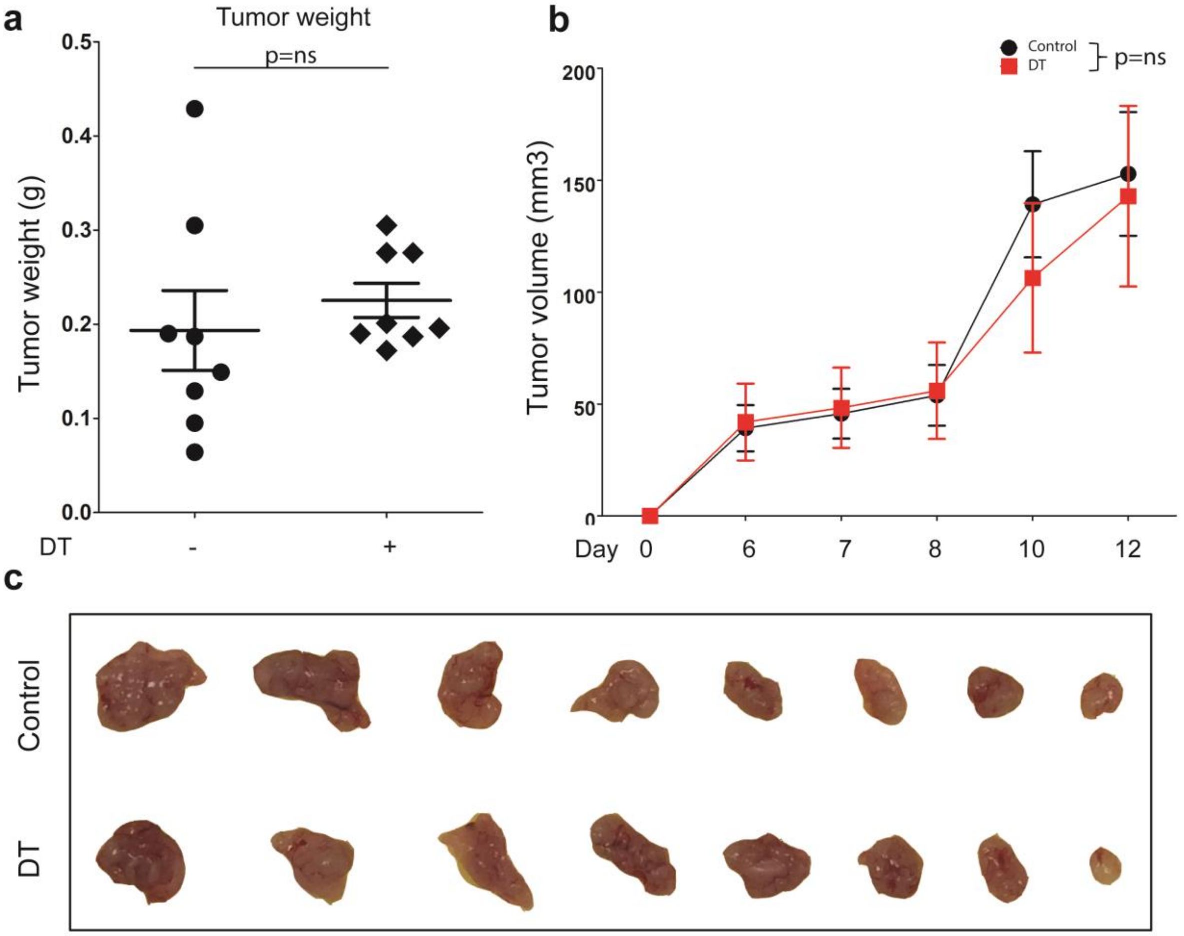

Supplementary Figure 12 | Wide type tumor organoids do not respond to DT administration. a, The weight of tumors initiated by wild type organoids from control, the DT-treated group following sacrifice of the animals involved (Control vs. DT: 0.19 ± 0.042 g vs. 0.23 ± 0.018 g, n = 8, P = ns, Mean ± SEM). **b,** Representative growth curve showing tumor volume in the control group and the DT-treated group (n = 4). **c,** Representative pictures showing the tumors from the control group and DT administrated group. Source data are provided as a Source Data file.

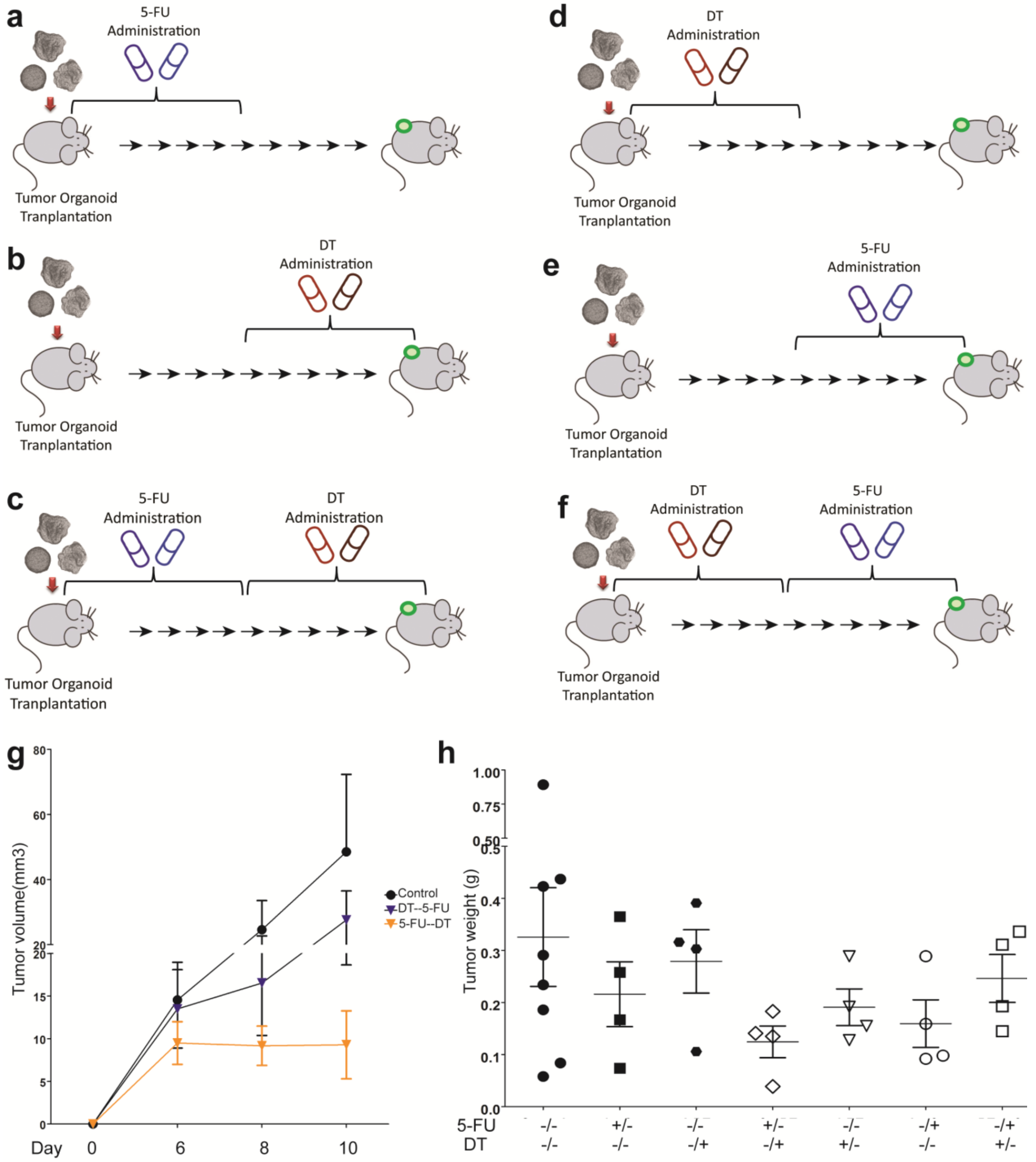

Supplementary Figure 13 | Effects of combining DT treatment with conventional anti-cancer therapy. **a,** 5-FU control: 5-FU was administrated during the first half of the experiment period and tumor volume was assessed continuously. **b**, DT control: animals were treated with DT during the second half of the experiment period and tumor volume was assessed continuously. **c**, Combination strategy 1: 5-FU was administrated during the first half and DT was treated during the second half of the experiment period. Tumor volume was assessed continuously. (**a** and **b** are the relevant control groups for **c**). **d**, DT control: DT was administrated during the first half of the experiment period and tumor volume was assessed continuously. **e**, 5-FU control: 5-FU was administrated during the second half of the

experiment period the and tumor volume was assessed continuously. **f**, Combination strategy 2: First DT was applied (first half of the experiment) followed by 5-FU treatment (second half of the experiment) (**d** and **e** are the relevant control groups for **f**). **g**, Representative growth curve showing tumor volumes of the control, Combination strategy 1 (5-FU--DT) and Combination strategy 2 (DT--5-FU) group (*n* = 4). **h**, Weight of tumors from the different groups described above at the end of the experiment. +/-: Treatment was administrated for the first half of the experiment; -/+: Treatment was administrated for the second half of the experiment; -/-: No treatment. Mean ± SEM. Source data are provided as a Source Data file.

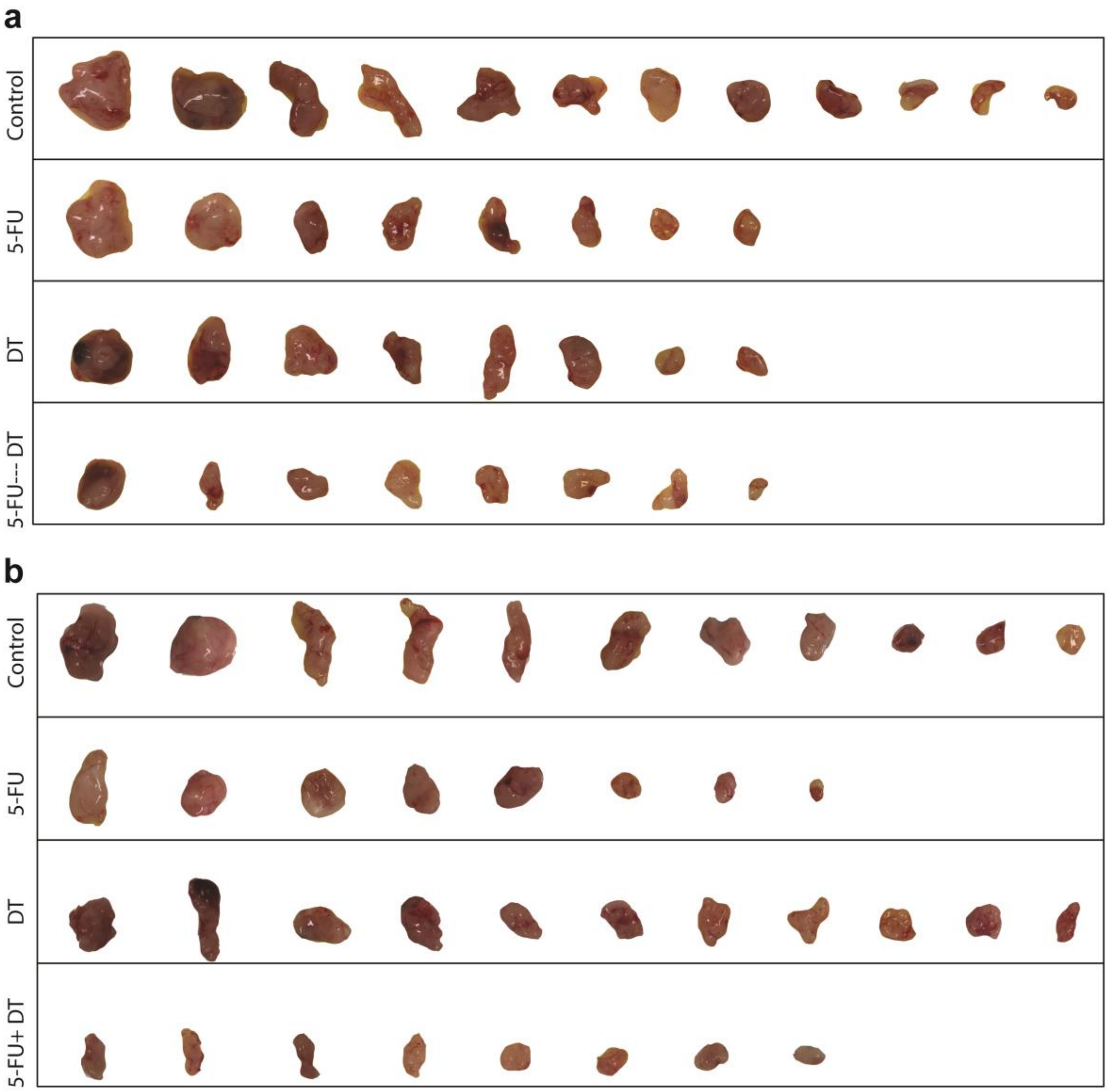

Supplementary Figure 14 | Combination of LGR5 lineage ablation with conventional therapy. a-b, Pictures showing the tumors from the different groups.

Supplementary Table 1 | Allograft tumor

Allograft Tumor						
Initiated Tumor Code	Isolated Cell Popualtion	Original Strain	Injected Cell Number	Initiated Tumor Weight(g)	Tumor Formation Time	Immunodeficient mice type
1	LGR5+	SAL1	72	0.58	23	Nude mice
	LGR5-		72	0.27	23	Nude mice
2	LGR5+	SAL1	57	0.44	23	Nude mice
	LGR5-		57	0.01	23	Nude mice
3	LGR5+	SAL1	90	0.14	23	Nude mice
	LGR5-		90	0.11	23	Nude mice
4	LGR5+	SAL1	167	0.22	23	Nude mice
	LGR5-		167	0.08	23	Nude mice
5	LGR5+	AL43	66	2.28	38	Nude mice
	LGR5-		66	0.45	38	Nude mice
6	LGR5+	AL43	53	1.11	38	NSG
	LGR5-		53	0.43	38	NSG
7	LGR5+	AL43	57	0.51	38	NSG
	LGR5-		57	0.77	38	NSG
8	LGR5+	SAL2	237	0.8	26	Nude mice
	LGR5-		237	0.05	26	Nude mice
9	LGR5+	SAL2	699	0.23	26	Nude mice
	LGR5-		699	0.1	26	Nude mice
10	LGR5+	SAL1	182	0.53	26	Nude mice
	LGR5-		182	0.09	26	Nude mice
11	LGR5+	SAL1	127	0.18	26	Nude mice
	LGR5-		127	0.61	26	Nude mice

Supplementary Table 2 | Clinical-pathological data of Erasmus cohort

**Table. Patient characteristics**	
**Characteristic**	**HCC patients (n=74)**
**Age at surgery (years)**	
Mean ± SD	60 ± 15,9
Median (range)	63 (11-82)
**Sex – no. (%)**	
Male	45 (60,8)
Female	29 (39,2)
**Race – no. (%)**	
White	61 (82,4)
African	6 (8,1)
Asian	6 (8,1)
Not reported	1 (1,4)
**Etiology – no. (%)**	
No known liver disease	21 (28,4)
Alcohol	16 (21,6)
Hepatitis B	9 (12,2)
NASH	8 (10,8)
Hepatitis C + Alcohol	6 (8,1)
Hepatitis B + Alc/HepC/NASH	5 (6,8)
Hepatitis C	5 (6,8)
Fibrolamellar HCC	3 (4,1)
Hemochromatosis + NASH	1 (1,4)
**Hepatitis status – no. (%)**	
Hepatitis B or C positive	25 (33,8)
Chronic Hepatitis B	14 (18,9)
Chronic Hepatitis C	12 (16,2)
**Cirrhosis – no. (%)**	
Yes	21 (28,4)
No	53 (71,6)
**Tumor differentiation – no. (%)**	
Good	8 (10,8)
Moderate	40 (54,1)
Poor	14 (18,9)
Unknown	12 (16,2)
**Vascular invasion – no. (%)**	
Yes	29 (39,2)
No	38 (51,4)
Unknown	7 (9,5)
**Number of lesions – no. (%)**	
1	40 (54,1)
>1	34 (45,9)
**Size of largest lesion (cm)**	
Mean ± SD	7,7 ± 5,6
Median (range)	6,1 (1-24)
**AFP level before resection (ug/l)**	
Mean ± SD	6661,1 ± 407289,4
Median (range)	8 (1-3118700)

Supplementary Table 3 | Antibody

Antibody	Antibody clone/ reference	Raised	Origin
CD45	56-0451-82	Mouse	eBioscience
AFP	SAB3500533-100UG	Goat	Sigma
HNF4a	ab41898	Mouse	Abcam
EpCAM	ab71916	Rabbit	Abcam
Cytokeratin 19	ab52625	Rabbit	Abcam
GFP	A-11122	Rabbit	Invitrogen/Life Technologies
Alexa Fluor® 488 AffiniPure Donkey Anti-Goat IgG (H+L)	705-545-147	Donkey	Bio-Connect
Donkey anti-Rabbit IgG (H+L) Secondary Antibody, Alexa Fluor® 594 conjugate	R37119	Donkey	Thermo fisher
Donkey anti-mouse IgG (H+L) Secondary Antibody, Alexa Fluor® 594 conjugate	R37115	Donkey	Thermo fisher

Supplementary Table 4 | Primer

Gene name	Gene Symbo	Sequence	
Leucine-rich repeat-containing G-protein coupled receptor 5	Mouse-*Lgr5*	Fw	CTG ACT TTG AAT GGT GCC TCG
		Re	ATG TCC ACT ACC GCG ATT AC
Cytokeratin-19	Mouse-*Krt19*	Fw	GTG AAG ATC CGC GAC TGG T
		Re	AGG CGA GCA TTG TC AAT CTG
Transcription factor SOX-9	Mouse-*Sox9*	Fw	CGA CTA CGC TGA CCA TCA GA
		Re	GAC TGG TTG TTC CCA GTG CT
CD133	Mouse-*Prom1*	Fw	TCT GTT CAG CAT TTC CTC AC
		Re	TCA GTA TCG AGA CGG GTC
CD44	Mouse-*CD44*	Fw	CGT CCA ACA CCT CCC ACT AT
		Re	AGC CGC TGC TGA CAT CGT
Keratin, type II cytoskeletal 7	Mouse-*Ck7*	Fw	ATC CGC GAG ATC ACC ATC
		Re	ATG TGT CTG AGA TCT GCG ACT
Leucine-rich repeats and immunoglobulin-like domains protein 1	Mouse-*Lrig1*	Fw	AAGGGAACTCAACTTGGCGAG
		Re	ACGTGAGGCCTTCAATCAGC
Octamer-binding transcription factor 4	Mouse-*Oct4*	Fw	CTGTAGGGAGGGCTTCGGGCACTT
		Re	CTGAGGGCCAGGCAGGAGCACGAG
Homeobox protein NANOG	Mouse-*Nanog*	Fw	AGGGTCTGCTACTGAGATGCTCTG
		Re	CAACCACTGGTTTTTCTGCCACCG
SRY (sex determining region Y)-box 2	Mouse-*Sox2*	Fw	GGCAGCTACAGCATGATGCAGGAGC
		Re	CTGGTCATGGAGTTGTACTGCAGG
Homeodomain-only protein	Mouse-*Hopx*	Fw	CATCCTTAGTCAGACGCGCA
		Re	AGGCAAGCCTTCTGACCGC
Telomerase reverse transcriptase	Mouse-*Tert*	Fw	GCAGGTGAACAGCCTCCAGACAG
		Re	TCCTAACACGCTGGTCAAAGGGAAGC
RNA-binding protein MEX3A	Mouse-*Mex3a*	Fw	ACACCACGGAGTGCGTTC
		Re	GTTGGTTTTGGCCCTCAGA
Mucin 5AC	Mouse-*Muc5ac*	Fw	GGACCAAGTGGTTTGACACTGAC
		Re	CCTCATAGTTGAGGCACATCCCAG
Epithelial cell adhesion molecule	Mouse-*Epcam*	Fw	CGCAGCTCAGGAAGAATGTG
		Re	TGAAGTACACTGGCATTGACG
SRY-box 17	Mouse-*Sox17*	Fw	GGCGCAGCAGAATCCAGA
		Re	CCACGACTTGCCCAGCAT
Cell surface associated or polymorphic epithelial mucin	Mouse-*Muc1*	Fw	CCCCAGTTGTCTGTTGGGGTC
		Re	GGATTCTACCACCACGGAGCC
Glyceraldehyde 3-phosphate dehydrogenase	Mouse-*Gapdh*	Fw	TCACCACCATGGAGAAGGC
		Re	GCTAAGCAGTTGGTGGTGCA
Beta-glucuronidases	Human-*GUSB*	Fw	CAGGTGATGGAAGAAGTGG
		Re	GTTGCTCACAAGGTCACAG
Hypoxanthine phosphoribosyltransferase 1	Human-*HPRT1*	Fw	GCTATAAATTCTTTGCTGACCTGCTG
		Re	AATTACTTTTATGTCCCCTGTTGACTGG
Phosphomannomutase 1	Human-*PMM1*	Fw	CGAGTTCTCCGAACTGGAC
		Re	CTGTTTTCAGGGCTTCCAC
Leucine-rich repeat-containing G-protein coupled receptor 5	Human-*LGR5*	Fw	TCAGTCAGCTGCTCCCGAAT
		Re	CGTTTCCCGCAAGACGTAAC

Supplementary Note:

Description for Supplementary Data

[1] Supplementary Data 1

Content:

The detailed information for *Lgr5-DTRGFP* mice which used to induce liver cancer, including:

1)The mice code

2)The mice background

3)Primary Code: The corresponding code of initiated primary organoid strain. Black mark: the tissue did not initiate an organoid strain or the strain was already lost due to infection in the following culture.

4)Post Den Time (Month): The sacrifice time after the induction of DEN.

5)DEN Administration (Week): Time passed between administration of DEN and sacrifice.

6)Tissue Type: S: tumor surrounding tissue; T: tumor tissue. S—T: initially marked with tumor surrounding tissue and then characterized as tumor.

7)Percentage of LGR5-expressing cells (%): The percentage of LGR5-expressing cells within each tissue.

8)Allograft Strains: The strain code which initiated allograft tumor in the immunodeficient mice.

Remarks: In total, 41 mice were monitored. 10 (mice code: 8,12,17,18,21,22,29,31,35,41) out of 41 mice liver did not show obvious tumor formation. After the following characterization, 2 (mice code: 8, 22) out of 10 were proven to be liver tumor.

[2] Supplementary Data 2

Content:

The H&E/Gomori/EpCAM/AFP/CK19/GFP staining of primary/allograft tissues for all the allograft strains.

[3] Supplementary Data 3

Content:

Gene enrichment analysis of the differentially expressed genes between Untreated LGR5+ Vs. LGR5-, 5-FU-treated LGR5+ Vs. LGR5-, 5-FU-treated Vs. Untreated LGR5+ and 5-FU-treated Vs. Untreated LGR5- cells.

[4] Supplementary Data 4

Content:

Gene: The differentially expressed genes between untreated LGR5+ cells and LGR5- cells.

Gene description: The gene description.

Survival Analyses: Online database (The Human Protein Atlas, http://www.proteinatlas.org/).

P value for Survival Analyses: Online database (The Human Protein Atlas, http://www.proteinatlas.org/).

[5] Supplementary Data 5

Content:

The detailed information for single cells isolated from DEN induced murine livers and allograft tumors, then used for organoid initiation, including:

1)Code: The corresponding tissues of DEN induced murine livers or allograft tumors. Green mark: the groups did initiate organoids after sorting.

2) Initiated organoid number for each group.

3) Organoid initiated efficiency (%) for each group.

Remarks: In total 89 tissues were collected. Among them, 71 tissues were sorted for following single cell initiation; 18 tissues were failed for the sort due to FACS machine issue or other technical reasons.

[6] Supplementary Data 6

Content:

The detailed information for single cells isolated from DEN induced murine livers, then injected directly into immunodeficient mice for tumor formation, including:

1)Code: The corresponding tissues of DEN induced murine livers and initiated allograft tumors. Green mark: the groups did initiate tumors after sorting.

2) Injected cell number for each group.

3) Pictures of primary tumors and corresponding allograft tumors.
