# Supplementary material for: LGR5 marks targetable tumor-initiating cells in mouse liver cancer
Source: Nat Commun. 2020 Apr 23;11:1961. doi: 10.1038/s41467-020-15846-0 (PMC7181628; doi:10.1038/s41467-020-15846-0)
Supplement: Supplementary file 3 — Description of Additional Supplementary Information [file 41467_2020_15846_MOESM3_ESM.pdf]

Description of Additional Supplementary Files

File Name: Supplementary Data 1
Description: The detailed information for Lgr5-DTRGFP mice which used to induce liver cancer, including: 1)The mice code 2)The mice background 3)Primary Code: The corresponding code of initiated primary organoid strain. Black mark: the tissue did not initiate an organoid strain or the strain was already lost due to infection in the following culture. 4)Post Den Time (Month): The sacrifice time after the induction of DEN. 5)DEN Administration (Week): Time passed between administration of DEN and sacrifice. 6)Tissue Type: S: tumor surrounding tissue; T: tumor tissue. S—T: initially marked with tumor surrounding tissue and then characterized as tumor. 7) Percentage of LGR5-expressing cells (%): The percentage of LGR5-expressing cells within each tissue. 8)Allograft Strains: The strain code which initiated allograft tumor in the immunodeficient mice.

File Name: Supplementary Data 2
Description: The H&E/Gomori/EpCAM/AFP/CK19/GFP staining of primary/allograft tissues for all the allograft strains.

File Name: Supplementary Data 3
Description: Gene enrichment analysis of the differentially expressed genes between Untreated LGR5+ Vs. LGR5-, 5-FU-treated LGR5+ Vs. LGR5-, 5-FU-treated Vs. Untreated LGR5+ and 5-FU-treated Vs. Untreated LGR5- cells.

File Name: Supplementary Data 4
Description: Gene: The differentially expressed genes between untreated LGR5+ cells and LGR5- cells.

File Name: Supplementary Data 5
Description: The detailed information for single cells isolated from DEN induced murine livers and allograft tumors, then used for organoid initiation, including: 1) Code: The corresponding tissues of DEN induced murine livers or allograft tumors. Green mark: the groups did initiate organoids after sorting. 2) Initiated organoid number for each group. 3) Organoid initiated efficiency (%) for each group.

File Name: Supplementary Data 6
Description: The detailed information for single cells isolated from DEN induced murine livers, then injected directly into immunodeficient mice for tumor formation, including: 1) Code: The corresponding tissues of DEN induced murine livers and initiated allograft tumors. Green mark: the groups did initiate tumors after sorting. 2) Injected cell number for each group. 3) Pictures of primary tumors and corresponding allograft tumors.
