# Supplementary material for: LGR5 marks targetable tumor-initiating cells in mouse liver cancer
Source: Nat Commun. 2020 Apr 23;11:1961. doi: 10.1038/s41467-020-15846-0 (PMC7181628; doi:10.1038/s41467-020-15846-0)
Supplement: Supplementary file 4 — Supplementary Data 1 [file 41467_2020_15846_MOESM4_ESM.pdf]

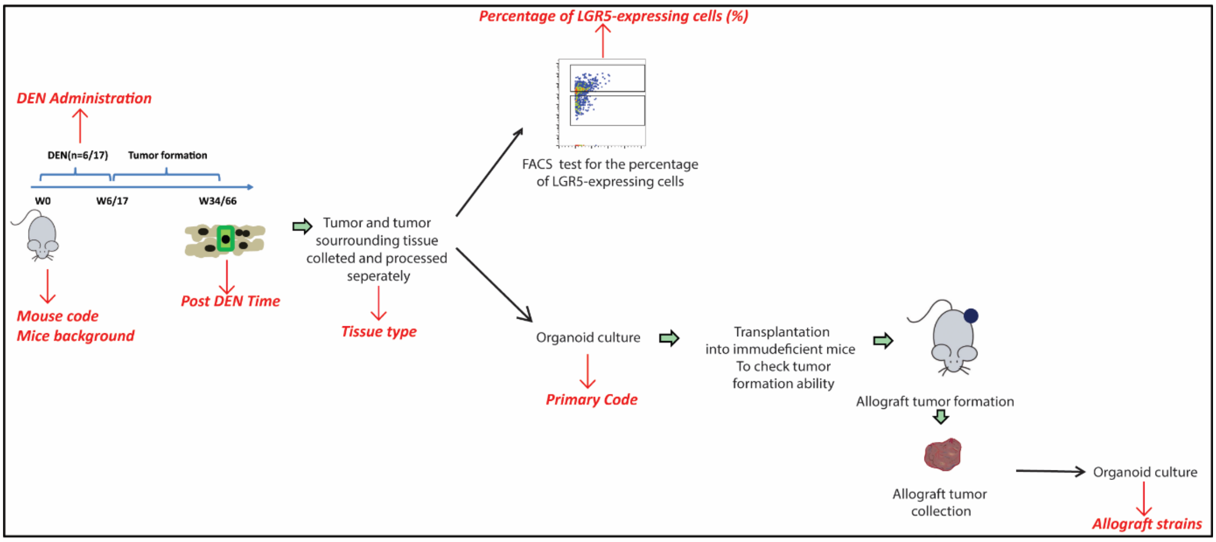
Percentage of LGR5-expressing cells (%)
DEN Administration
DEN(n=6/17)
Tumor formation
W0
W6/17
W34/66
Mouse code
Mice background
Post DEN Time
Tumor and tumor sourrounding tissue colleted and processed seperately
Tissue type
FACS test for the percentage of LGR5-expressing cells
Organoid culture
Primary Code
Transplantation into immudeficient mice To check tumor formation ability
Allograft tumor formation
Allograft tumor collection
Organoid culture
Allograft strains

Mouse Code	Mice Backgroud	Primary Code	Post Den Time (Month)	DEN Administration (Week)	Tissue Type	Percentage of LGR5-expressing cells (%)	Allograft Strains
M1	B6	PT1	7	6	S	0,28	
		PT2			T	0,16	
M2	B6	PT3	7	6	S	0,79	
		PT4			T	1,1	
M3	B6	PT5	12	6	S	0,16	
		PT6			T	0,18	
M4	B6	PT7	9	6	S	0,57	
		PT8			T1	5,66	AL8
		PT9			T2	0,32	
		PT10			T3	5,61	AL10
M5	B6	PT11	9	6	S	0,22	
		PT12			T	2,5	
M6	B6&C3H	PT13	5	17	S	0	AL13
		PT14			T	2,75	
M7	B6	PT15	13	6	S	0,4	
		PT16			T	2,07	
M8	B6	PT17	13	6	S--T	0,31	AL17
M9	B6	PT18	12	6	S	1,1	
		PT19			T	3,05	
M10	B6	PT20	15	6	S	0,85	
		PT21			T1	2,85	
		PT22			T2	1,43	
M11	B6	PT23	15	6	S	0,89	
		PT24			T	0,17	

Mouse Code	Mice backgroud	Primary code	Post Den time	DEN		Lgr5 expression	Allograft strains
M12	B6&C3H	PT25	3	11	S	0,18	
M13	B6	PT26	10	17	S	0,062	
		PT27			T	0,3	
M14	B6	PT28	13	6	S	0,33	
		PT29			T1	0,47	
		PT30			T2	2,15	
M15	B6	PT31	13	6	S	0	
		PT32			T1	0,45	
		PT33			T2	0,5	
M16	B6	PT34	13	6	S	0,12	
		PT35			T	1,71	
M17	B6	PT36	13	6	S	0,72	
M18	B6	PT37	13	6	S	0,24	
M19	B6	PT38	13	6	S	3,41	AL38
		PT39			T	1,11	
M20	B6	PT40	16	6	S	0,15	
		PT41			T	0,22	
M21	B6	PT42	14	6	S	0,33	
M22	B6&C3H	PT43	7	17	S--T	0,77	AL43
M23	B6&C3H	PT44	7	17	S	3,08	
		PT45			T1	3,29	
		PT46			T2	0,05	AL46

Mouse Code	Mice backgroud	Primary code	Post Den time	DEN		Lgr5 expression	Allograft strains
M24	B6&C3H	PT47	7	17	S	25	
		PT48			T1	46,1	
		PT49			T2	55,6	
		PT50			T3	51,2	
M25	B6	PT51	14	6	T	4,63	
M26	B6	PT52	14	6	S	0,021	
		PT53			T	0	
M27	B6&C3H	PT54	7	17	T	7,73	
M28	B6	PT55	8	17	S	20,3	
		PT56			T1	10,7	
		PT57			T2	1,67	
		PT58			T3	0,48	
		PT59			T4	4,66	
M29	B6	PT60	15	6	S	0,4	
M30	B6&C3H	PT61	8	17	T1	2,62	AL61
		PT62			T2	7,01	AL62
		PT63			T3	10,5	
M31	B6&C3H	PT64	8	17	S	0,3	
M32	B6	PT65	15	6	S	20,3	
		PT66			T1	1,04	
		PT67			T2	8,03	
M33	B6	PT68	15	6	T1	47,9	
		PT69			T2	17,4	
		PT70			T3	4,69	

Mouse Code	Mice backgroud	Primary code	Post Den time	DEN		Lgr5 expression	Allograft strains
M34	B6	PT71	15	6	S	17,4	
		PT72			T1	3,86	
		PT73			T2	21,6	
M35	B6	PT74	15	6	S	0,22	
M36	B6	PT75	15	6	S	0,25	
		PT76			T	3,41	
M37	B6	PT77	15	6	T1	1,41	
		PT78			T2	0,23	
		PT79			T3	0,65	
M38	B6&C3H	PT80	8	17	S	0,26	
		PT81			T	0,14	
M39	B6	PT82	13	6	T1	0,29	
		PT83			T2	5,13	
		PT84			T3	0,18	AL84
		PT85			T4	8.90	AL85
M40	B6&C3H	PT86	7	17	T1	14,6	
		PT87			T2	6,82	
		PT88			T3	13,9	
M41	B6&C3H	PT89	8	17	S	0,21	

Group Code	Group	Tissue Number	Lgr5 Expression
A	The collected mice liver tissue	89	5.583%
B	The mice liver which did not initiate tumor	8	0.325%
C	Tumor surrounding tissue	34	2.930%
D	Tumor tissue	55	7.294%
