# Supplementary material for: LGR5 marks targetable tumor-initiating cells in mouse liver cancer
Source: Nat Commun. 2020 Apr 23;11:1961. doi: 10.1038/s41467-020-15846-0 (PMC7181628; doi:10.1038/s41467-020-15846-0)
Supplement: Supplementary file 5 — Supplementary Data 2 [file 41467_2020_15846_MOESM5_ESM.pdf]

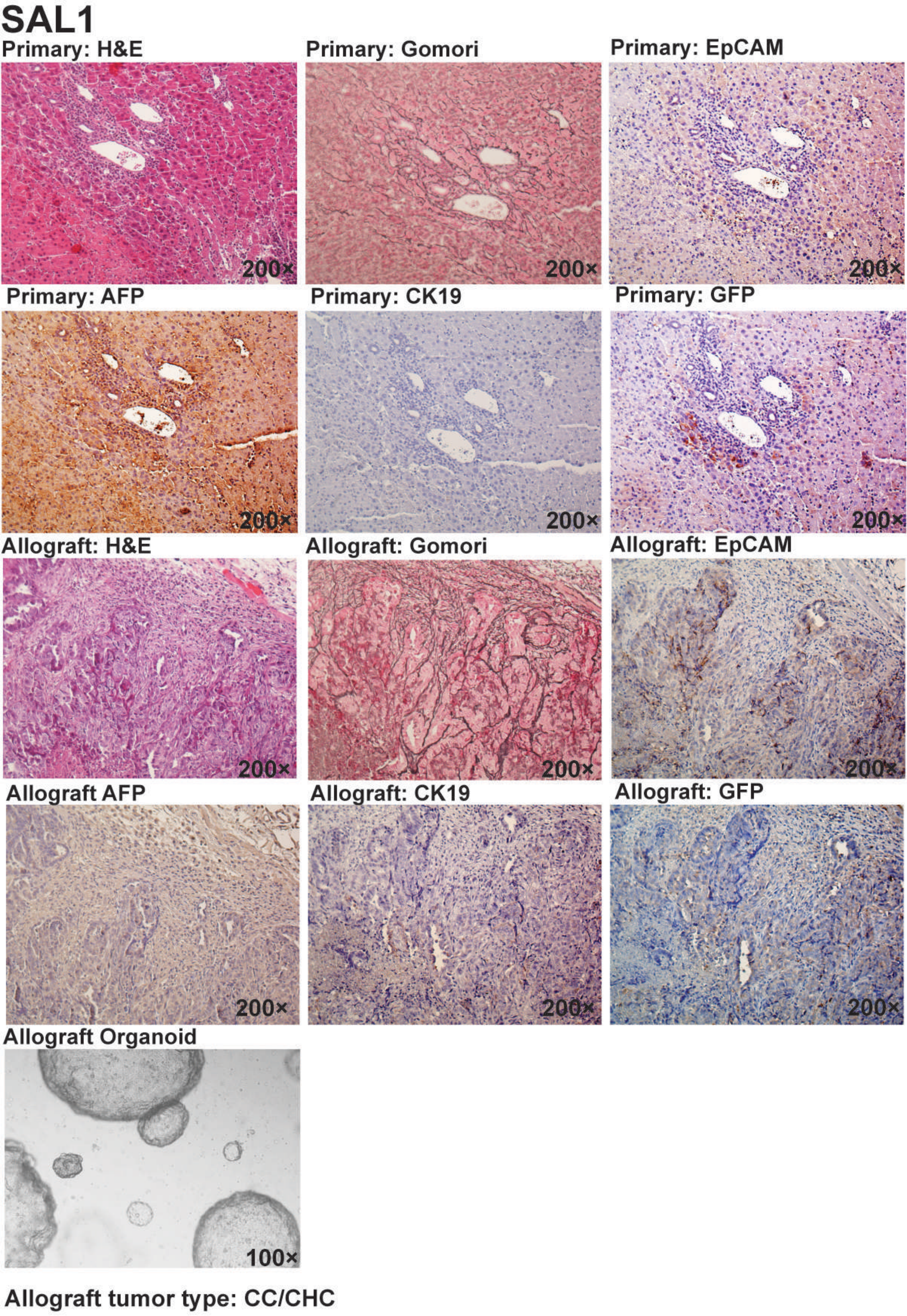
SAL1
Primary: H&E
200×
Primary: Gomori
200×
Primary: EpCAM
200×
Primary: AFP
200×
Primary: CK19
200×
Primary: GFP
200×
Allograft: H&E
200×
Allograft: Gomori
200×
Allograft: EpCAM
200×
Allograft AFP
200×
Allograft: CK19
200×
Allograft: GFP
200×
Allograft Organoid
100×
Allograft tumor type: CC/CHC

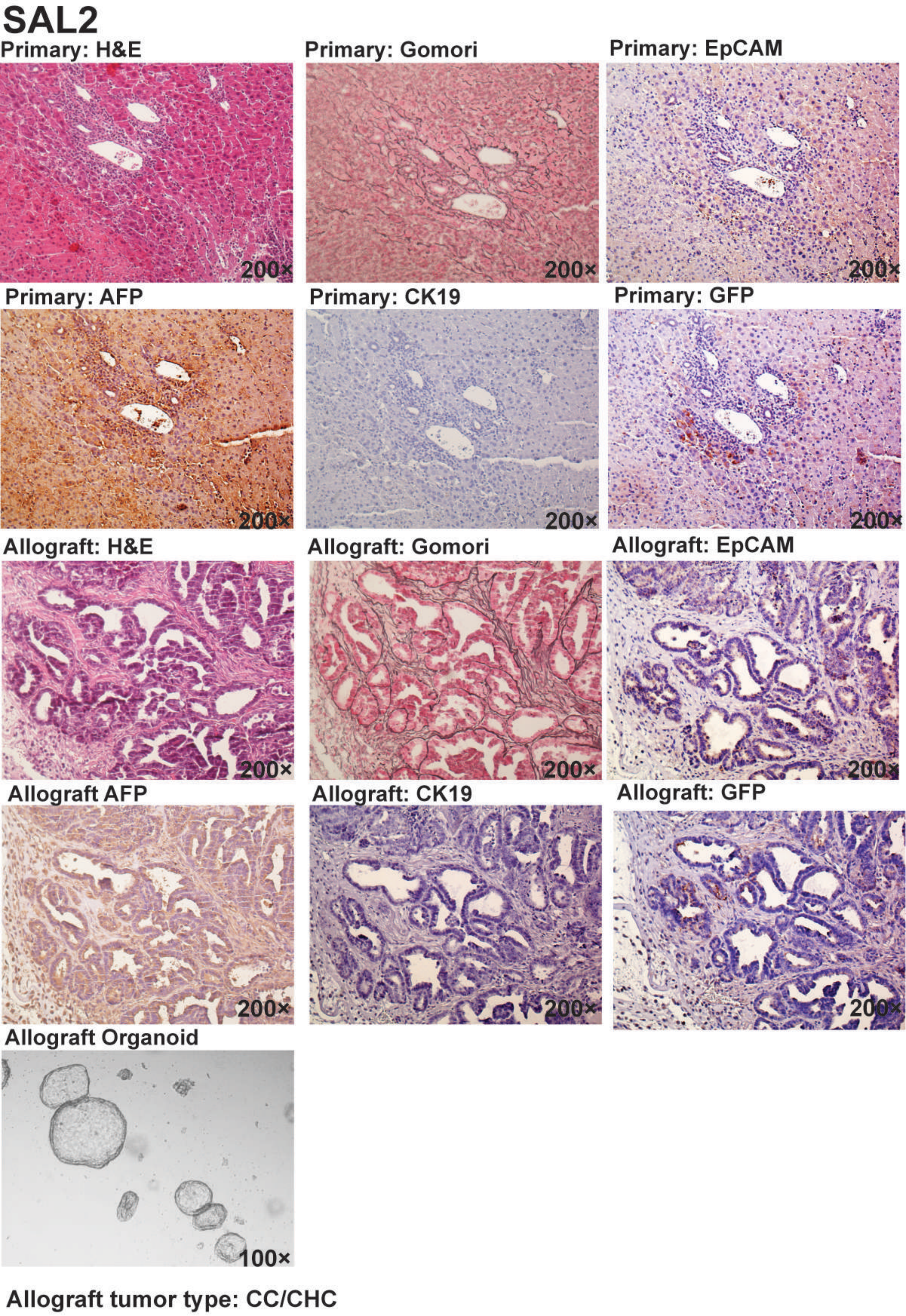
SAL2
Primary: H&E
200×
Primary: Gomori
200×
Primary: EpCAM
200×
Primary: AFP
200×
Primary: CK19
200×
Primary: GFP
200×
Allograft: H&E
200×
Allograft: Gomori
200×
Allograft: EpCAM
200×
Allograft AFP
200×
Allograft: CK19
200×
Allograft: GFP
200×
Allograft Organoid
100×
Allograft tumor type: CC/CHC

SAL3

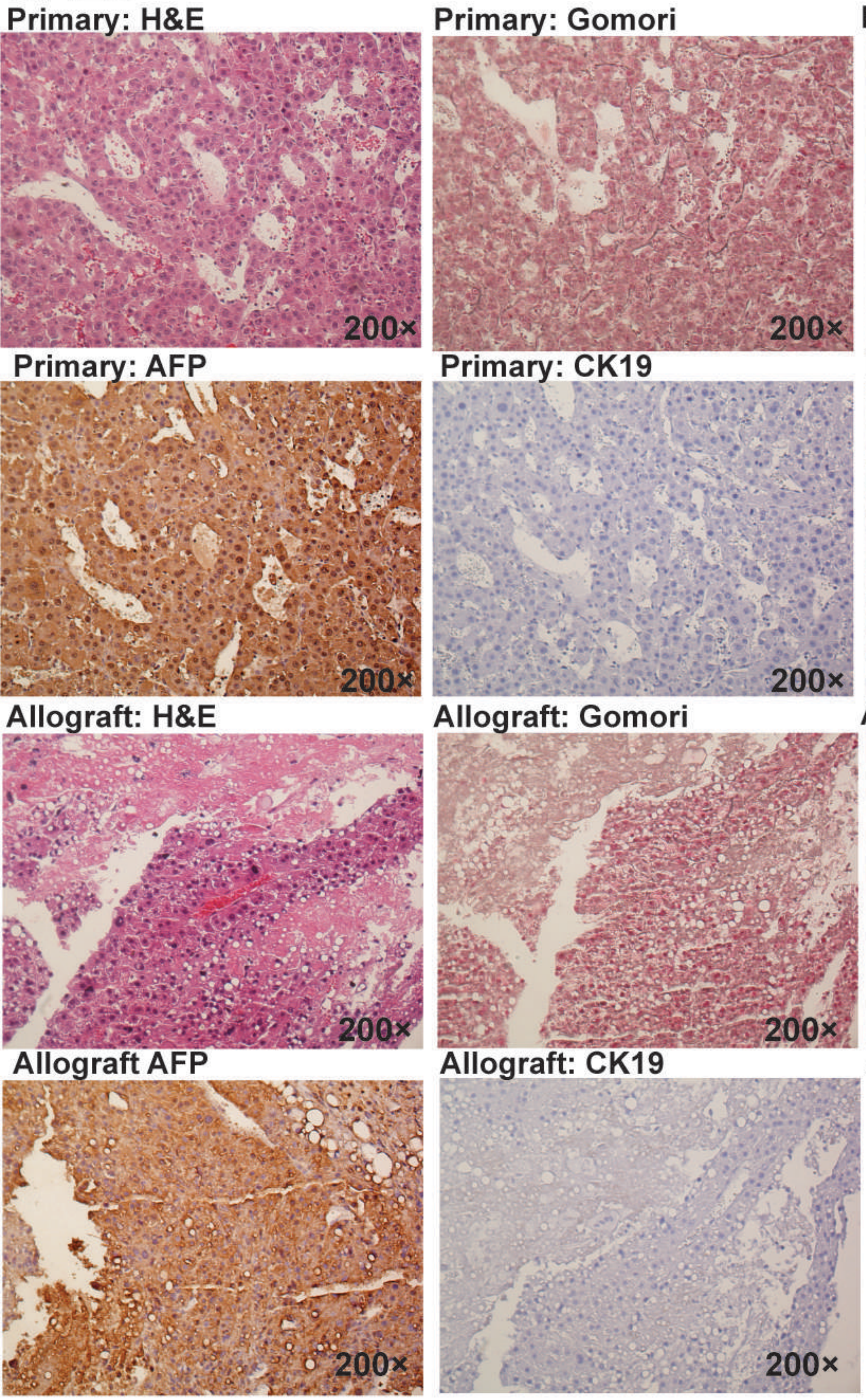

Allograft tumor type: HCC
Strain lost due to stop proliferation ex vivo

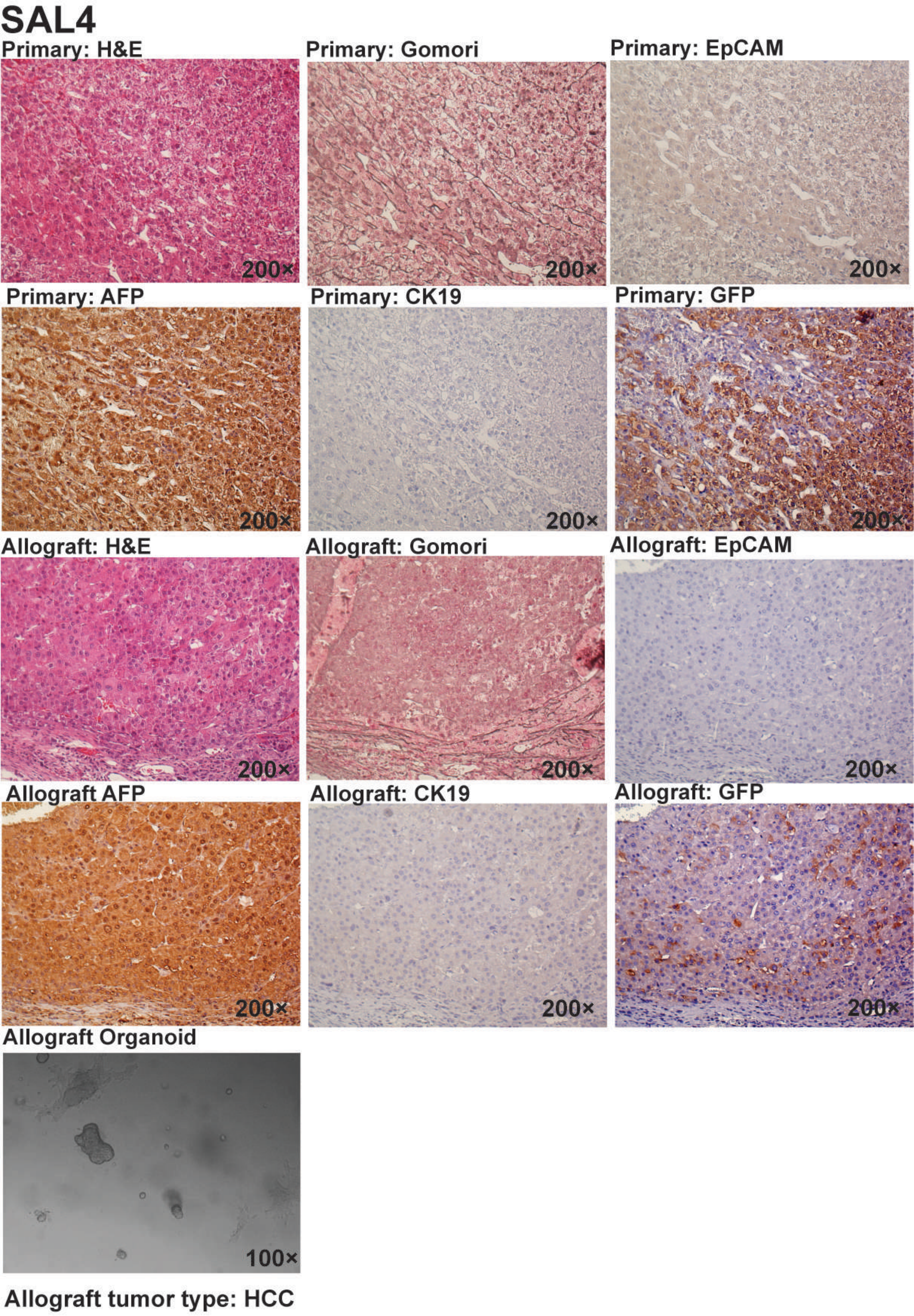
SAL4
Primary: H&E
200×
Primary: Gomori
200×
Primary: EpCAM
200×
Primary: AFP
200×
Primary: CK19
200×
Primary: GFP
200×
Allograft: H&E
200×
Allograft: Gomori
200×
Allograft: EpCAM
200×
Allograft AFP
200×
Allograft: CK19
200×
Allograft: GFP
200×
Allograft Organoid
100×
Allograft tumor type: HCC

AL8

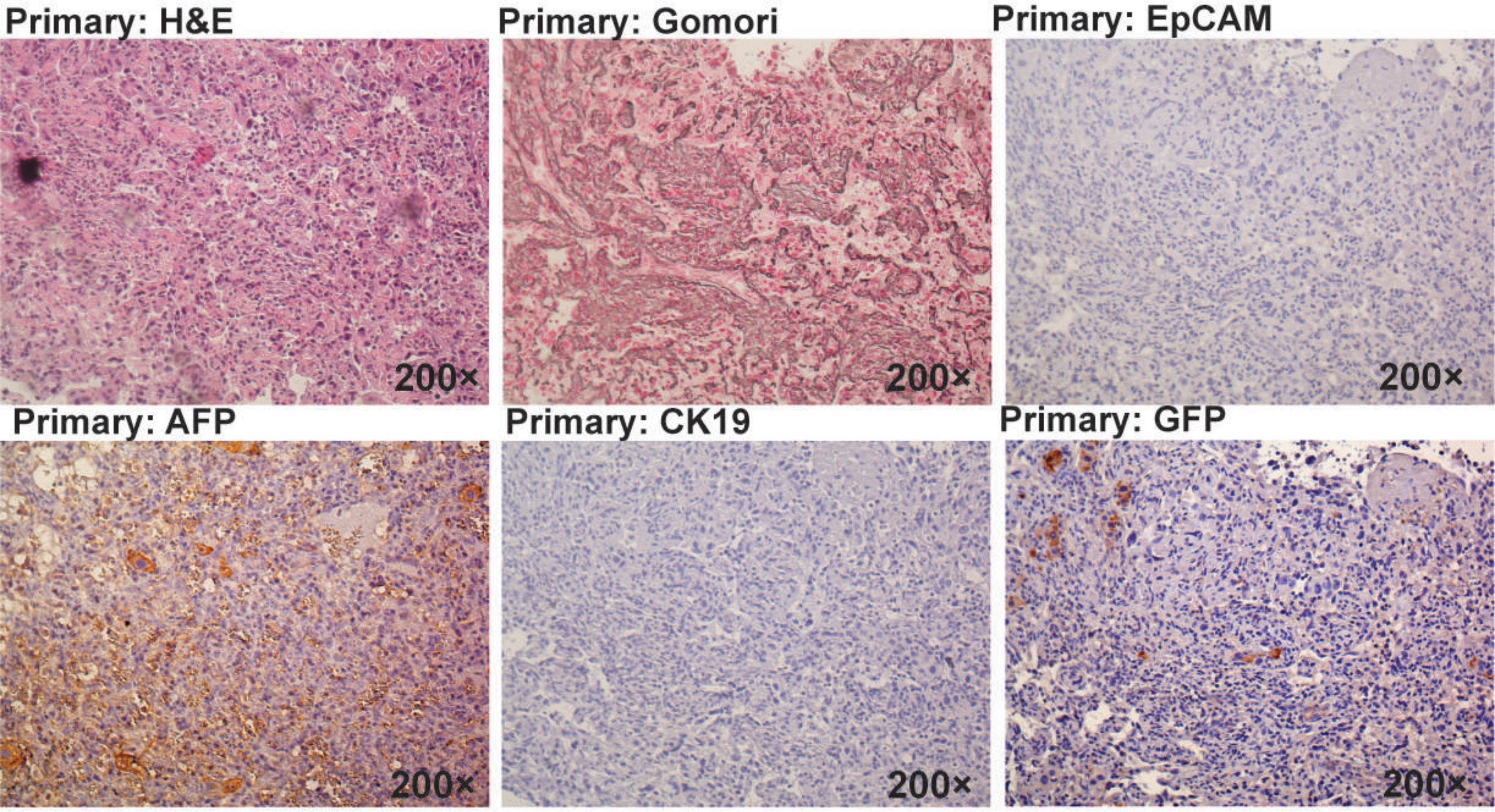

Allograft tumor type: Unclear
Strain lost due to stop proliferation ex vivo

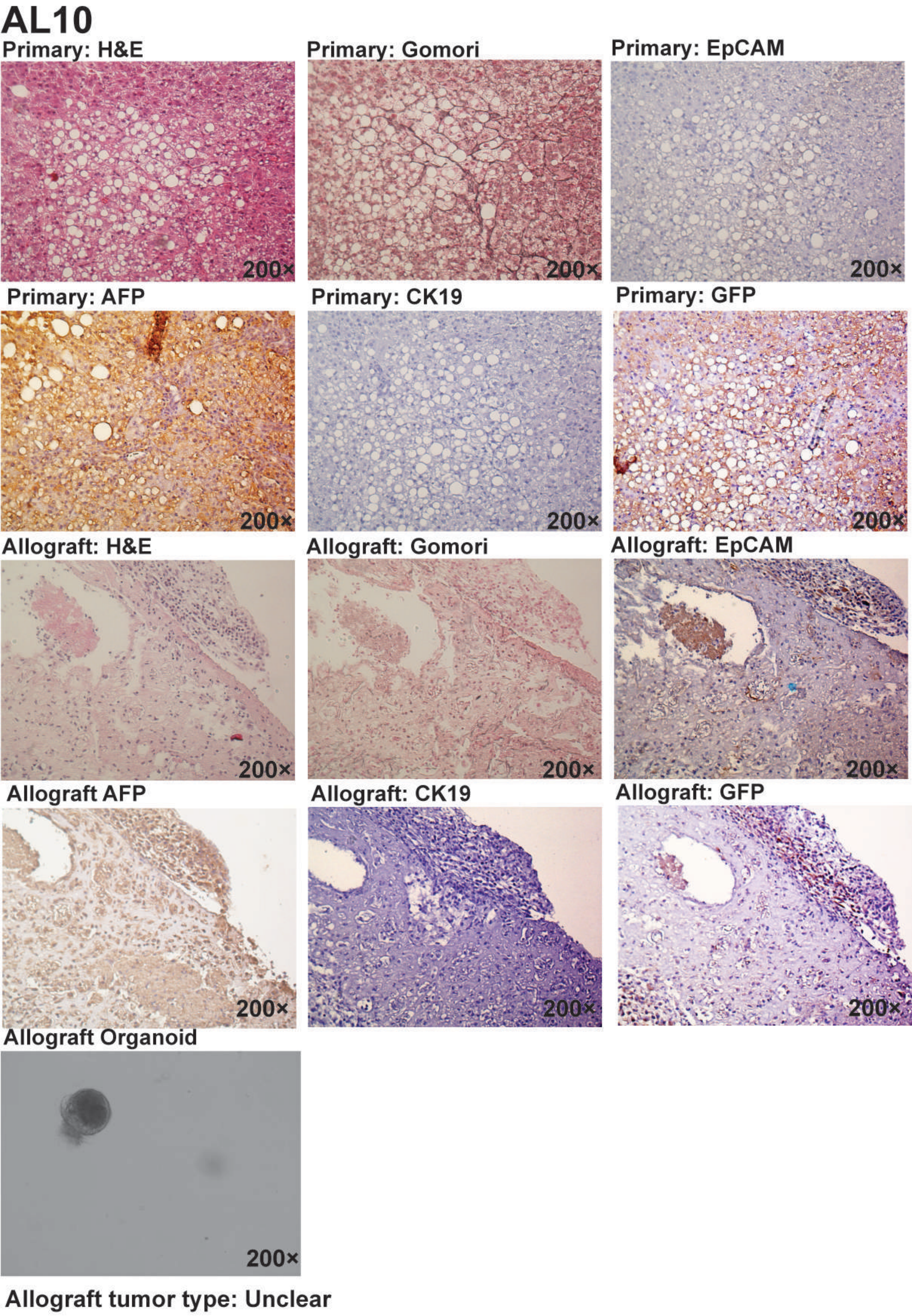
AL10
Primary: H&E
200×
Primary: Gomori
200×
Primary: EpCAM
200×
Primary: AFP
200×
Primary: CK19
200×
Primary: GFP
200×
Allograft: H&E
200×
Allograft: Gomori
200×
Allograft: EpCAM
200×
Allograft AFP
200×
Allograft: CK19
200×
Allograft: GFP
200×
Allograft Organoid
200×
Allograft tumor type: Unclear

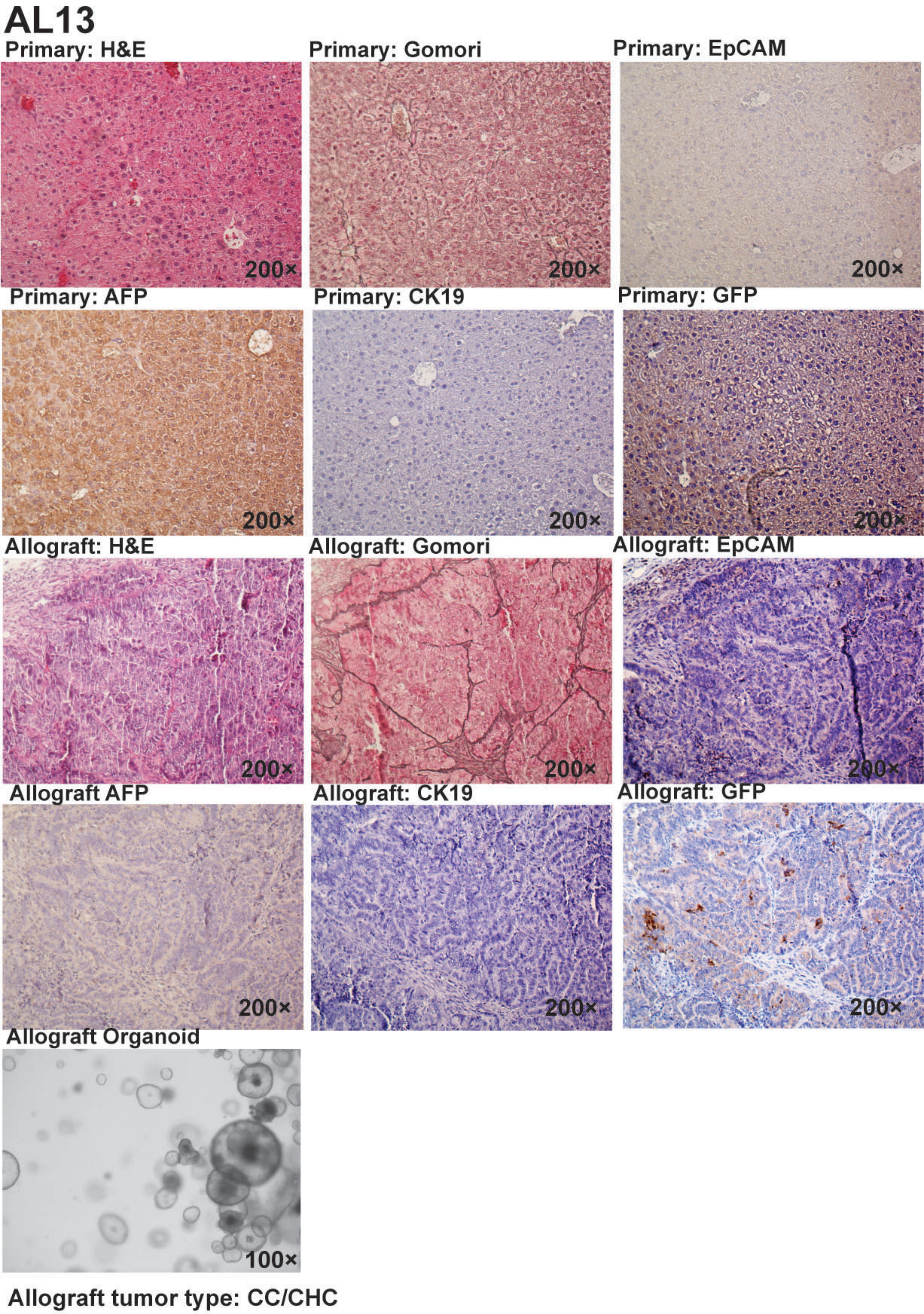
AL13
Primary: H&E
200×
Primary: Gomori
200×
Primary: EpCAM
200×
Primary: AFP
200×
Primary: CK19
200×
Primary: GFP
200×
Allograft: H&E
200×
Allograft: Gomori
200×
Allograft: EpCAM
200×
Allograft AFP
200×
Allograft: CK19
200×
Allograft: GFP
200×
Allograft Organoid
100×
Allograft tumor type: CC/CHC

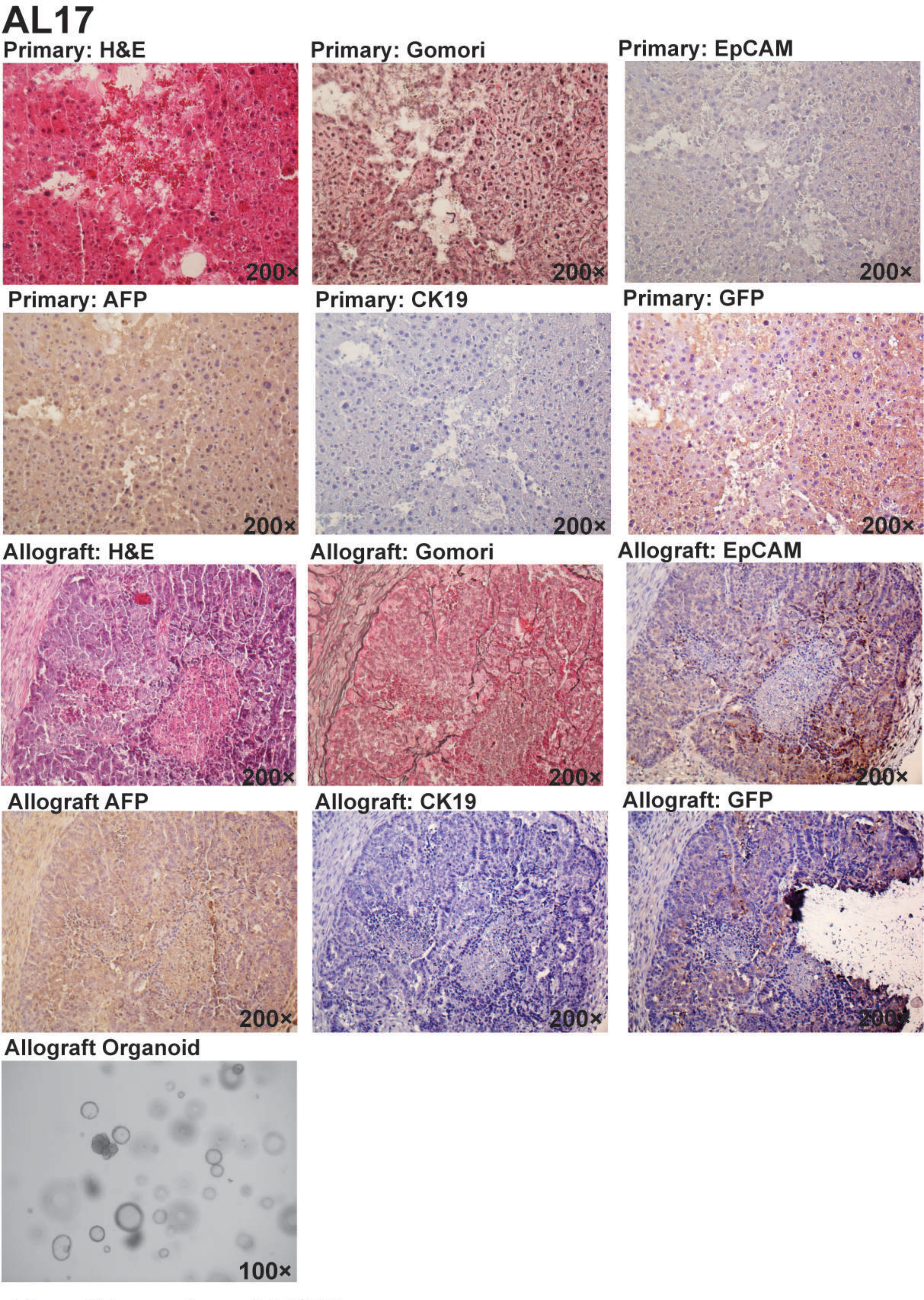

Allograft tumor type: CC/CHC

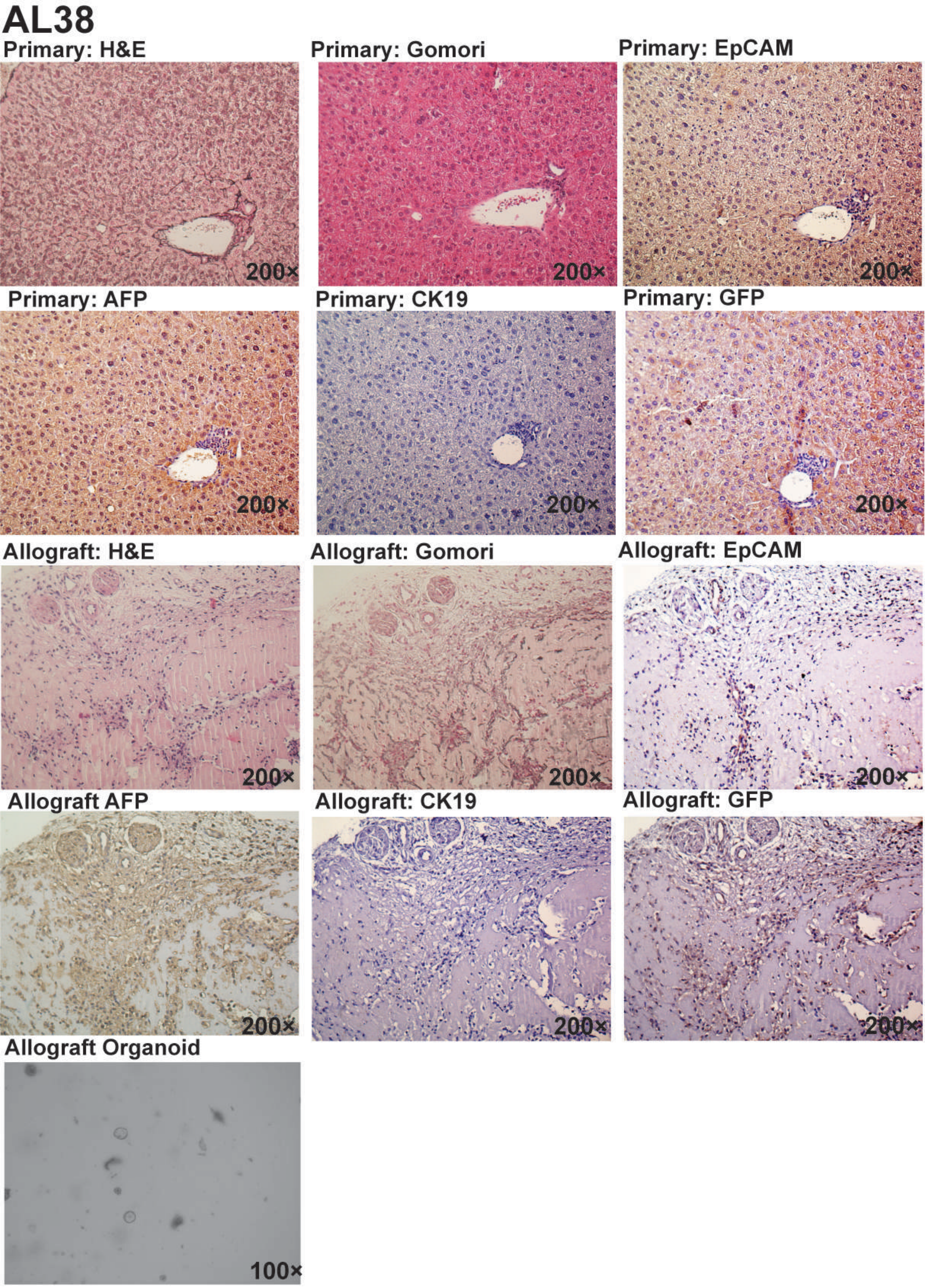

Allograft tumor type: Unclear

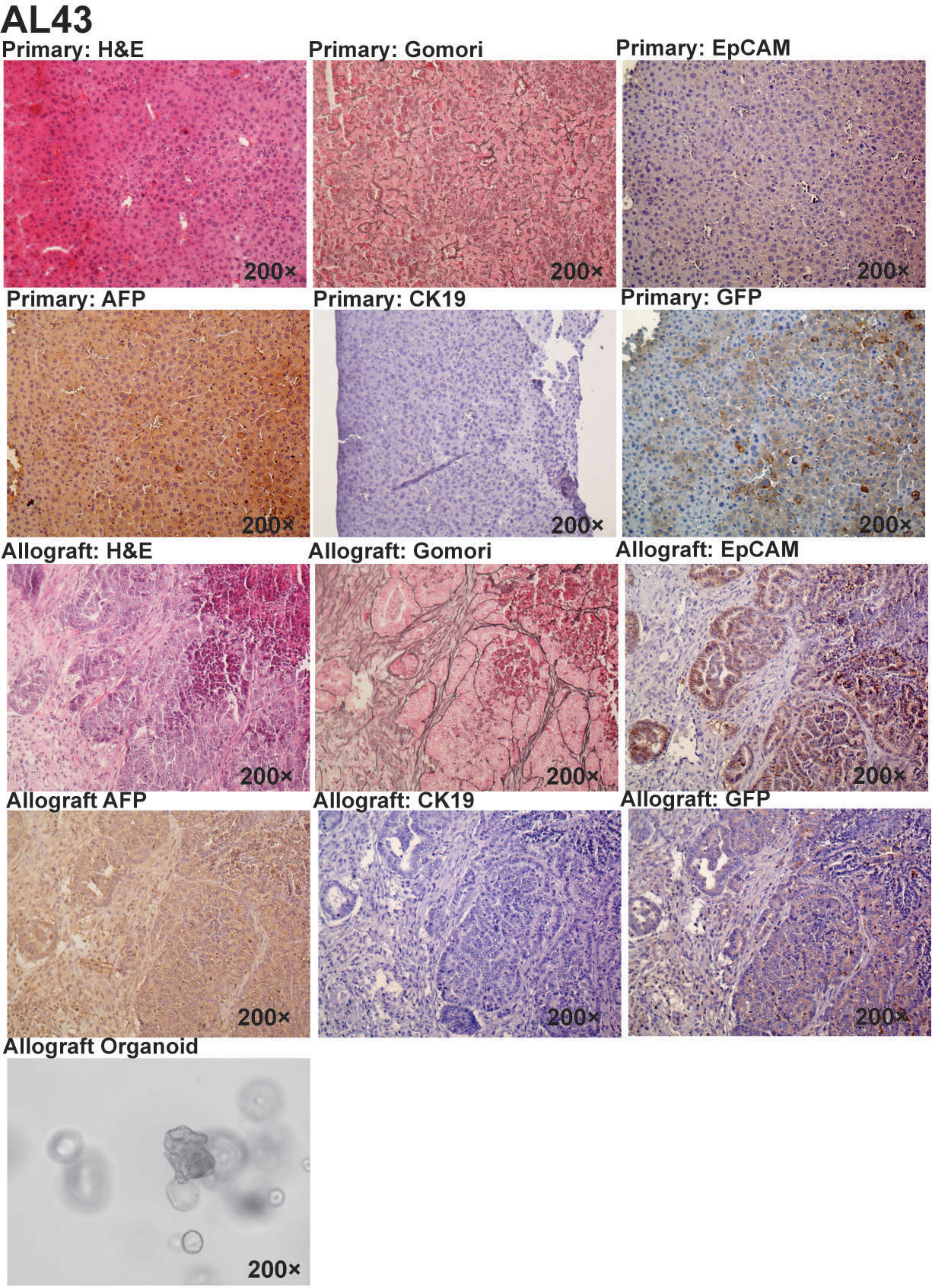

Allograft tumor type: CC/CHC

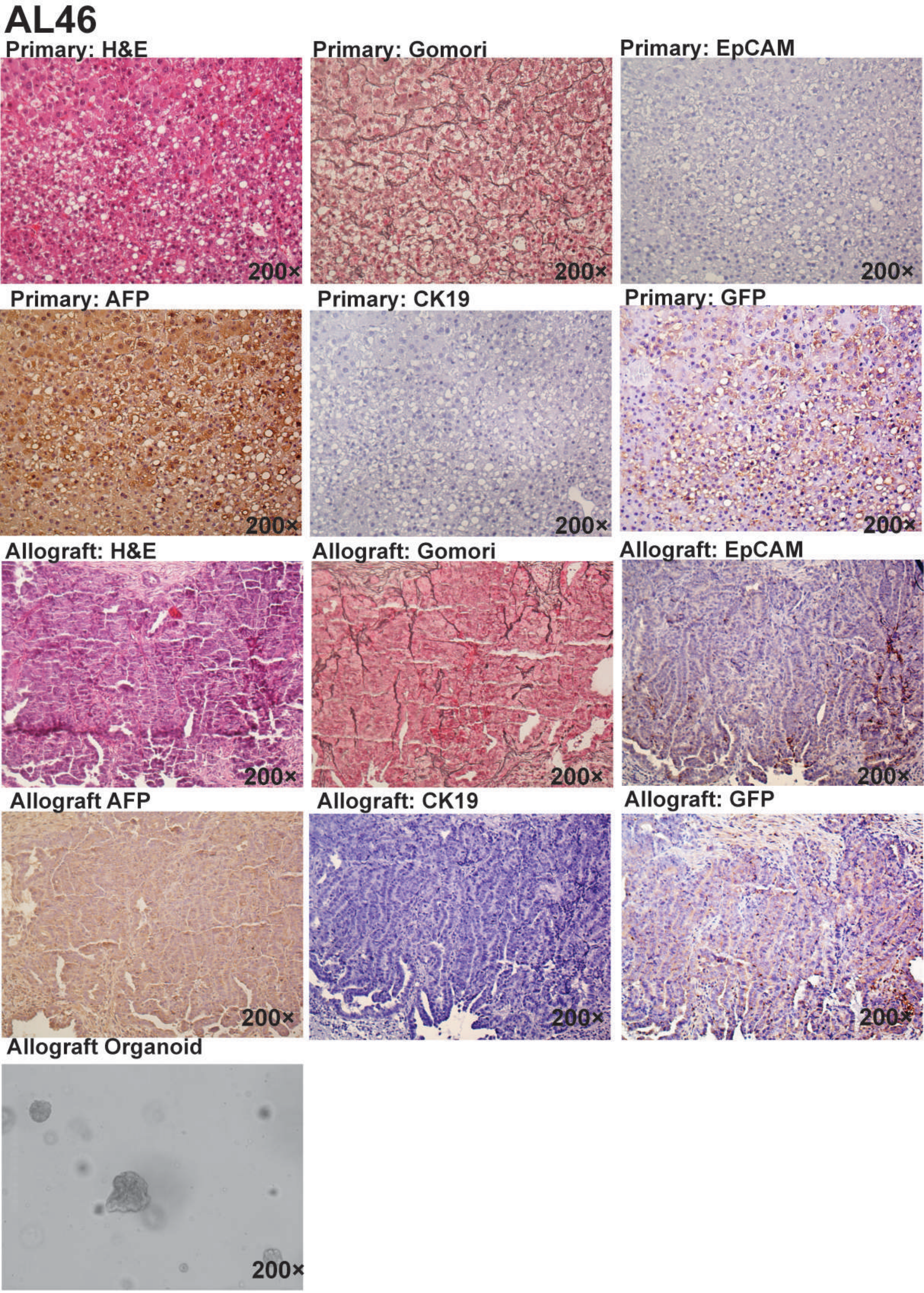

Allograft tumor type: CC/CHC

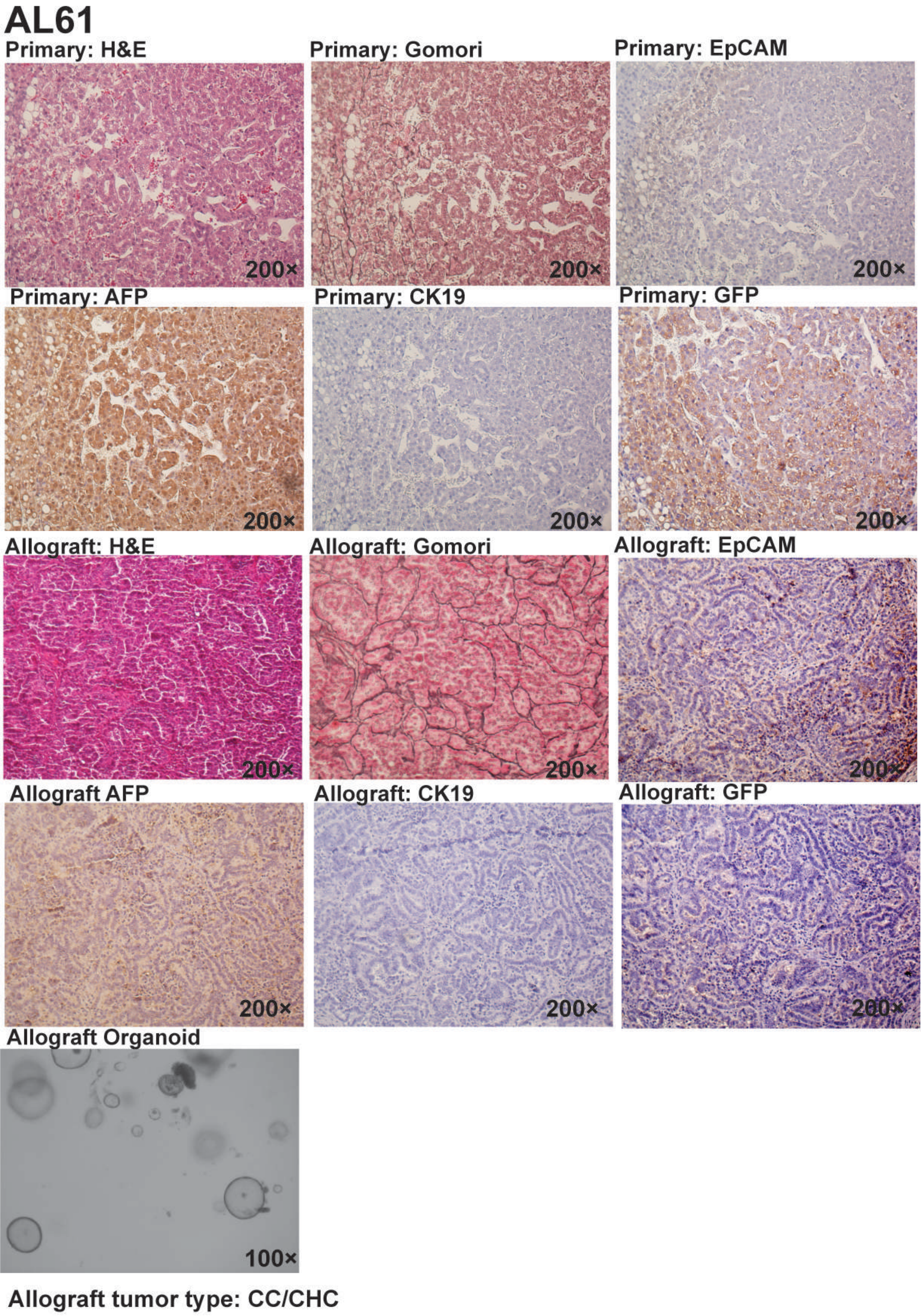
AL61
Primary: H&E
200×
Primary: Gomori
200×
Primary: EpCAM
200×
Primary: AFP
200×
Primary: CK19
200×
Primary: GFP
200×
Allograft: H&E
200×
Allograft: Gomori
200×
Allograft: EpCAM
200×
Allograft AFP
200×
Allograft: CK19
200×
Allograft: GFP
200×
Allograft Organoid
100×
Allograft tumor type: CC/CHC

AL62

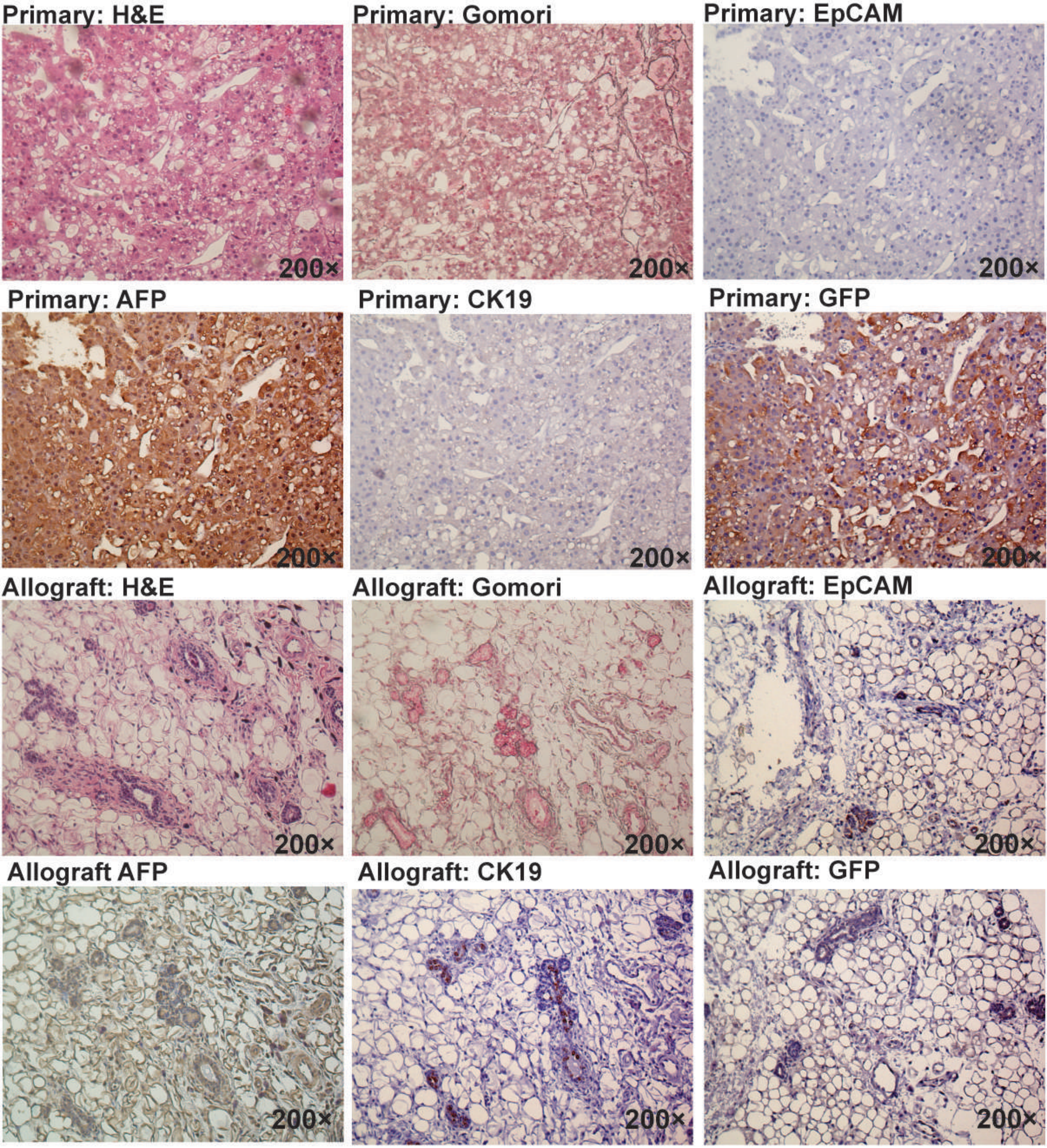

Allograft tumor type: unclear
Strain lost due to stop proliferation ex vivo

AL84

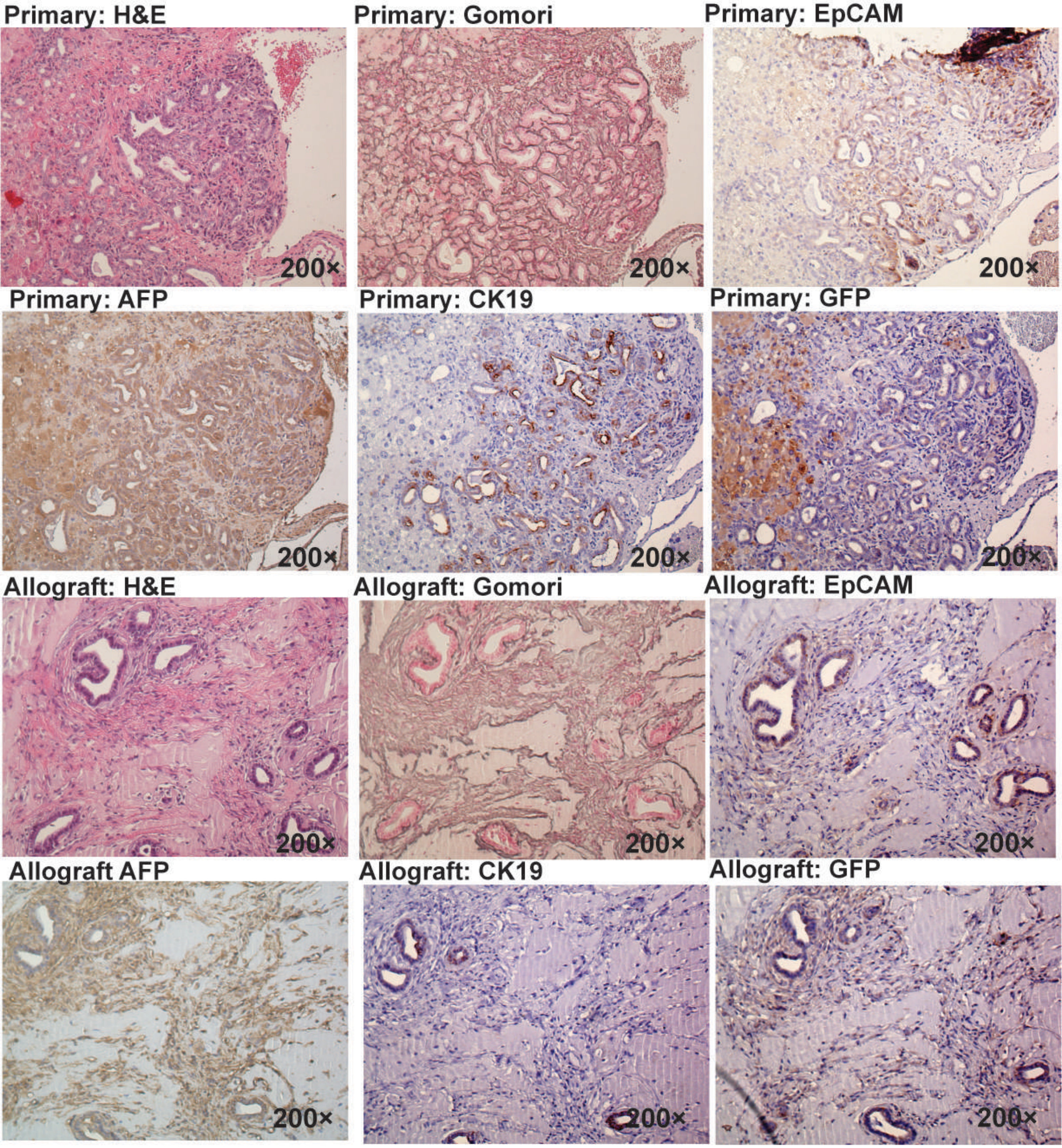

Allograft tumor type: CC
Strain lost due to stop proliferation ex vivo

AL85

Primary: H&E

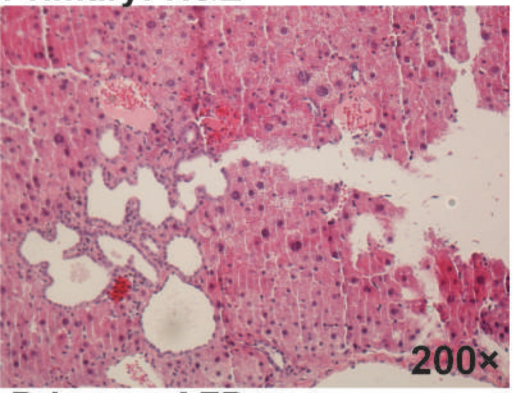

Primary: Gomori

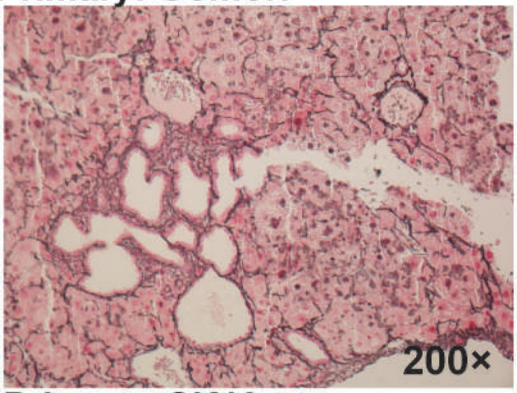

Primary: EpCAM

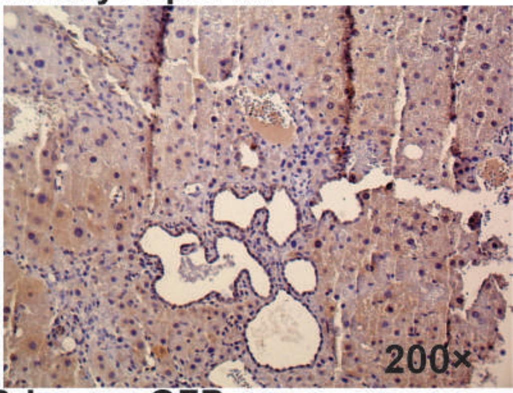

Primary: AFP

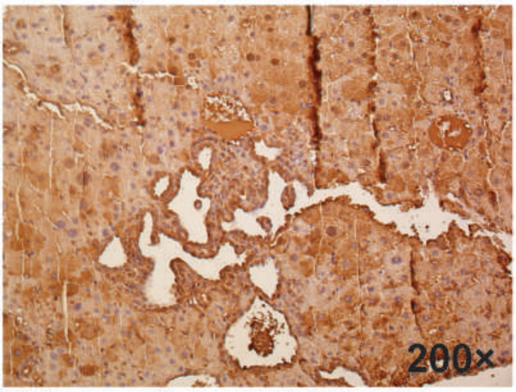

Primary: CK19

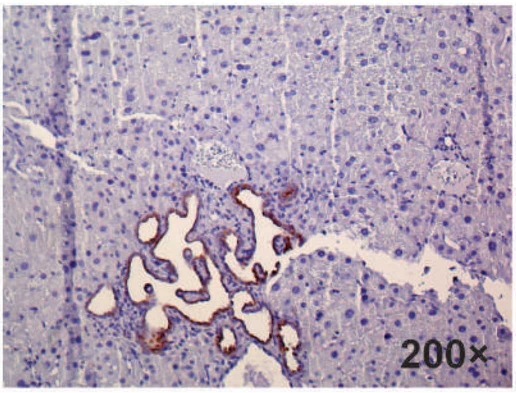

Primary: GFP

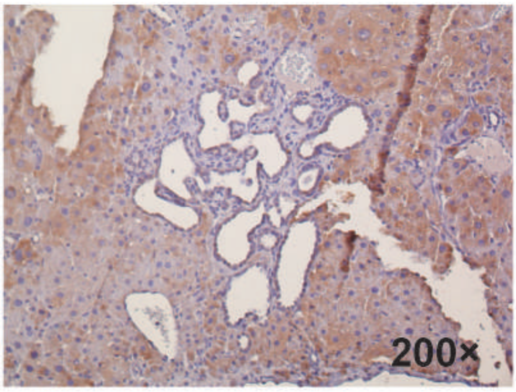

Primary Organoid

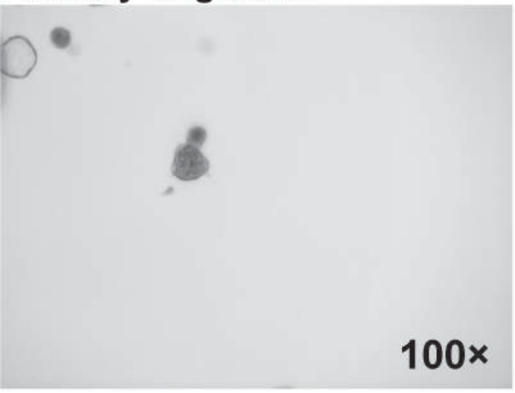

Allograft tumor type: Unclear

Strain lost due to stop proliferation ex vivo
