# Supplementary material for: LGR5 marks targetable tumor-initiating cells in mouse liver cancer
Source: Nat Commun. 2020 Apr 23;11:1961. doi: 10.1038/s41467-020-15846-0 (PMC7181628; doi:10.1038/s41467-020-15846-0)
Supplement: Supplementary file 7 — Supplementary Data 4 [file 41467_2020_15846_MOESM7_ESM.pdf]

Untreated LGR5+ vs. LGR5-

Upregulated Gene	Gene description	Survival Analyses	P value for Survival Analyses
Gm11042			
AL589879.2			
1700003G13Rik			
Snord93			
Gm24325			
Gm8326			
Taf7l	TATA-box binding protein associated factor 7 like	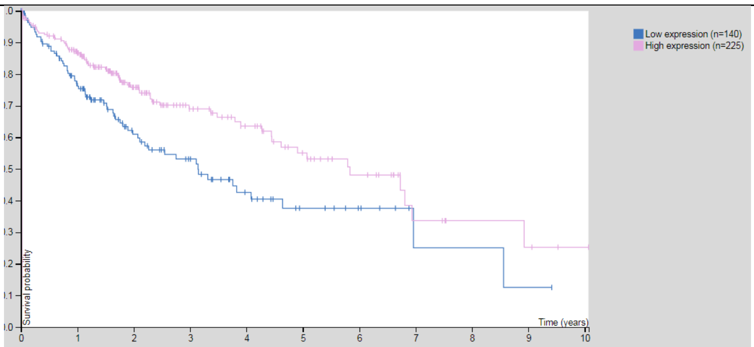	0.0039
Gm11977			
Gm24328			
Gm15558			
B230112G18Rik			
Gm42586			
Rpl36-ps7			
2210017I01Rik			
Gm43058			
Gm38321			
4833428L15Rik			
Tm6sf1	Transmembrane 6 superfamily member 1	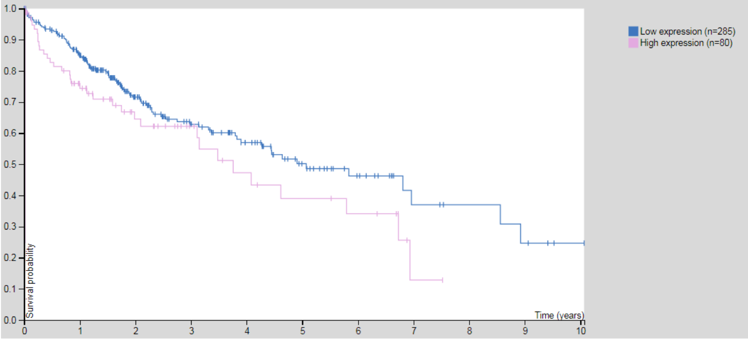	0.065
Gm18014			
Zfp572	Zfp572 zinc finger protein 572		
Kcnk4	Potassium two pore domain channel subfamily K member 4		

Spn	Sialophorin	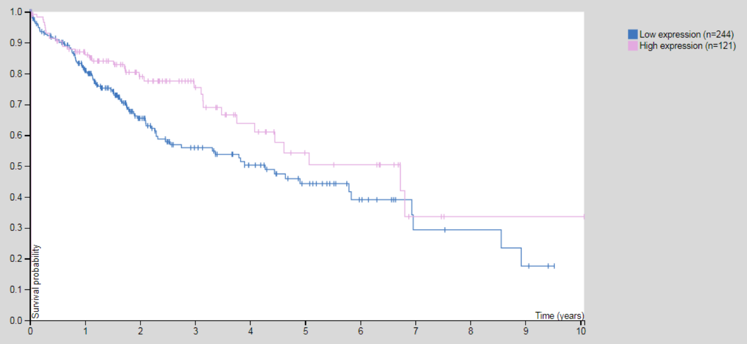	0.040
Smtnl1	Smoothelin like 1	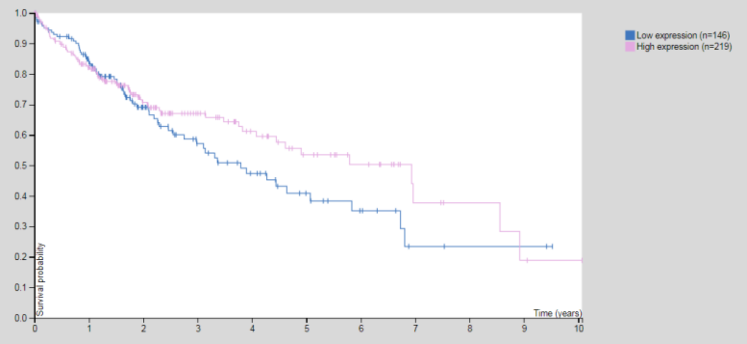	0.22
Gm12320			
Gm6976			
Gm24769			
Oas1h			
Tnfsfm13	tumor necrosis factor (ligand) superfamily, membrane-bound member 13		
Gm12227			
Gm12196			
Gm6568			
Gm15862			
Pitx3	Paired like homeodomain 3		
Rln1	Relaxin 1		
Fpr1	Formyl peptide receptor 1	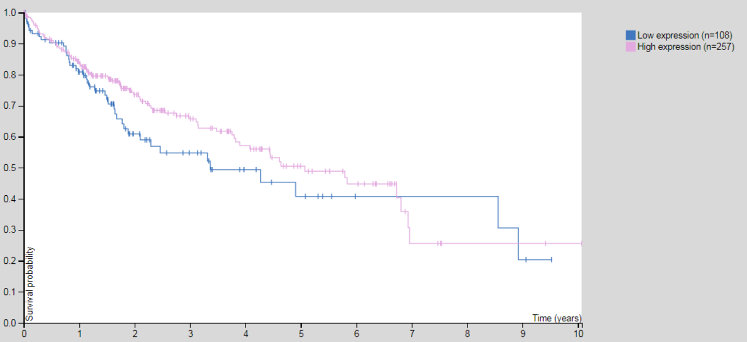	0.20
4933429H19Rik			
RP24-530N5.5			
Gm26899			
Ube4bos3			
Gm25445			
4933402D24Rik			
Mmp8	Matrix		

	metallopeptidase 8		
2300005B03Rik			
Fabp2	Fatty acid binding protein 2		
4930562D21Rik			
Gm38058			
Sox2	SRY-box 2	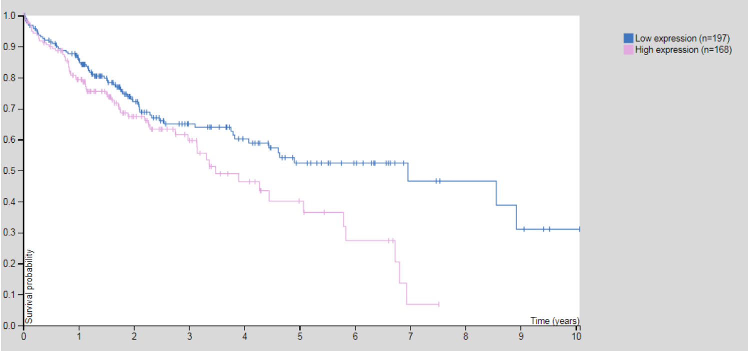	0.018
Gm4795			
Gm12043			
Dsg4	membrane proteins	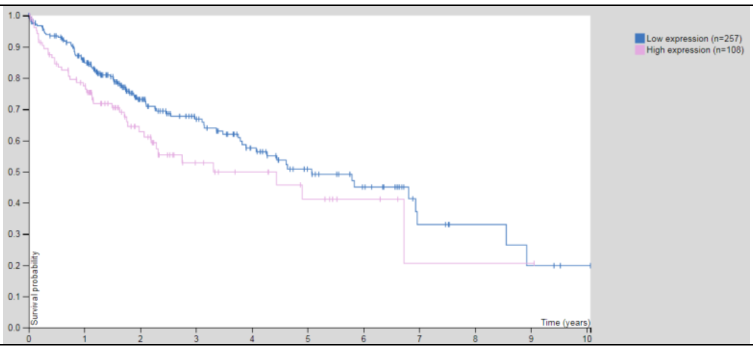	0.056
Gm15780			
Pnp2	Purine nucleoside phosphorylase		
Mcpt8	mast cell protease 8		
Nid1	Nidogen-1	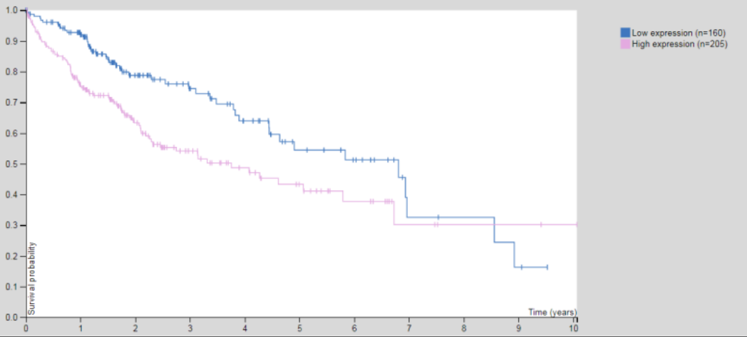	0.0024
Tshr	Thyroid stimulating hormone receptor		
Dnajb8	DnaJ heat shock protein family (Hsp40) member B8		
Nlrp4c	NACHT, LRR and PYD domains-containing protein 4C		
Gm10392			
Sstr1	Somatostatin		

	receptor 1		
Gm13578			
Gm14822			
Erich5	Glutamate rich 5	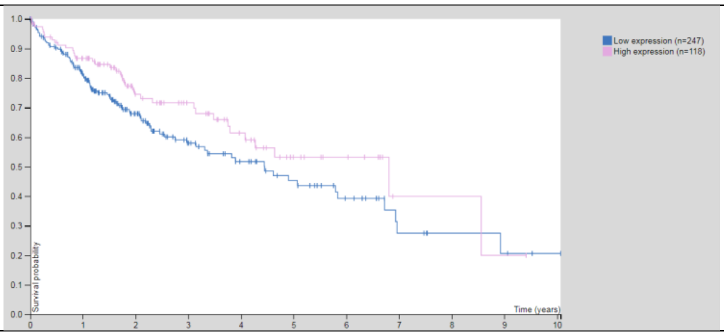	0.087
Gm18301			
6430571L13Rik			
Gm11465			
Gm15982			
Nkd2	Naked cuticle homolog 2		
Myl4	Myosin light chain 4		
Gm15964			
Gm42807			
Gm9765			
Gm16177			
Klhl3	Kelch like family member 3	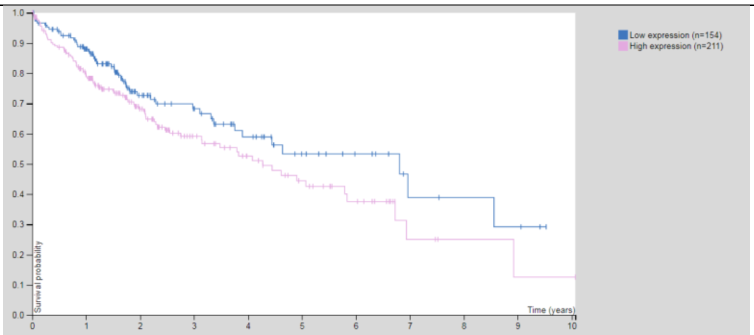	0.062
1700031P21Rik			
Gm24336			
Palm3	Paralemmin 3	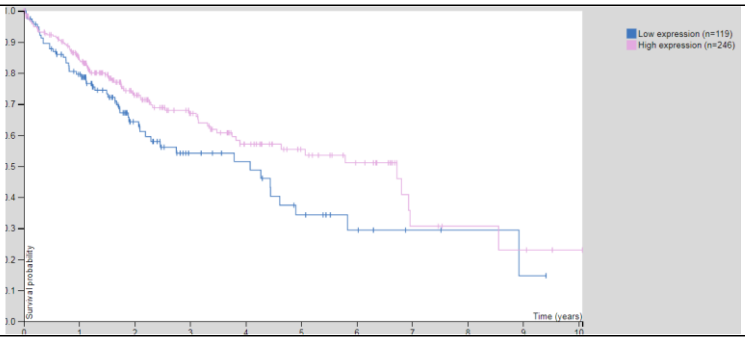	0.047
Gm14019			
Gm13110			
Gm42682			
Slc16a9	Solute carrier family 16 member 9		

Ambp	Alpha-1-microglobulin/bikunin precursor	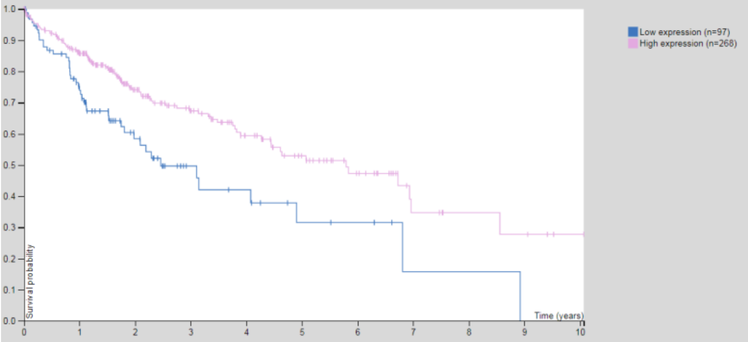	0.001
Gm37229			
Gm11764			
3110035E14Rik			
Gm6526			
Gm10030			
Mboat4	Membrane bound O-acyltransferase domain containing 4	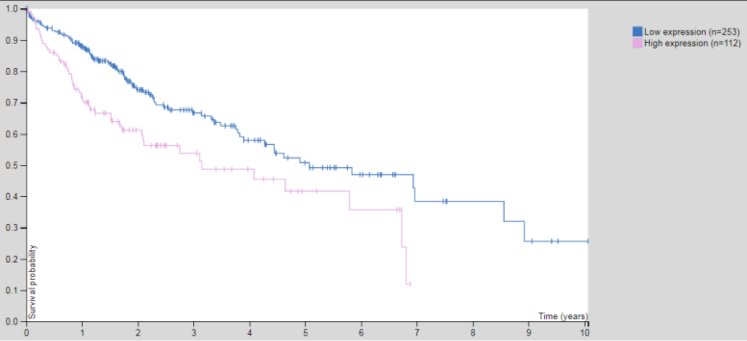	0.0019
Gm14379			
Cma1	Chymase 1	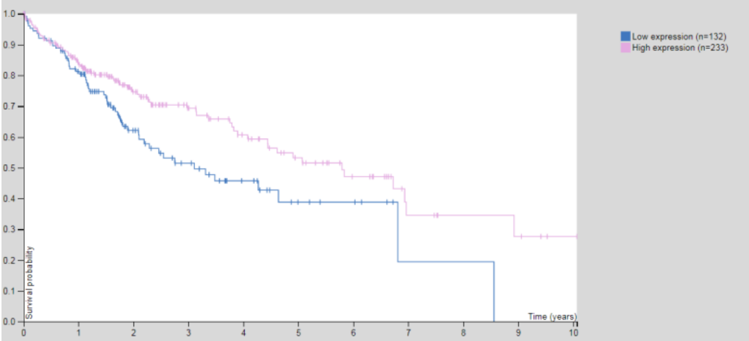	0.015
1600002D24Rik			
Gm5093			
Cd300lg	CD300 molecule like family member g		
Rab37	RAB37, member RAS oncogene family	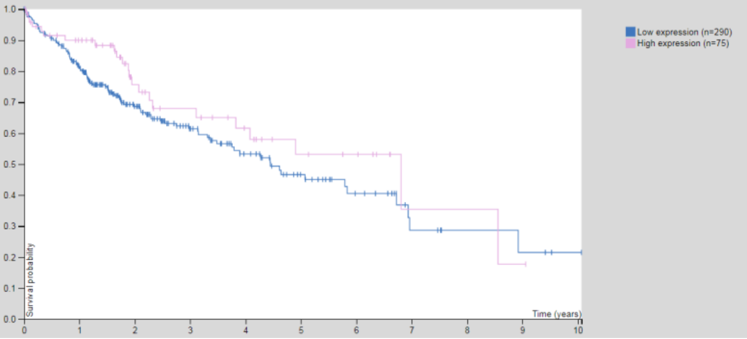	0.17
Gm28177			
Gm42500			
Hlx	H2.0 like homeobox		

Gm6226			
Gm37933			
Rai2	Retinoic acid induced 2		
Gm20056			
RP24-83C9.5			
Lyz2	Lysozyme C-2		
1700003M07Rik			
Gm5909			
Gm27033			
Gm13448			
Npm3-ps1			

Down regulated Gene	Gene description	Survival Analyses	P value for Survival Analyses
Gm12331			
Slc5a4b	Solute carrier family 5 (neutral amino acid transporters, system A)		
Defa-rs7			
Cks1brt	Cyclin-dependent kinases regulatory subunit 1		
Gm12868			
Gm7583			
Gm973			
Gm11401			
Gm17689			
Gm12583			
Gm14894			
Gm8101			
Gm13313			
Gm15516			
1700058P15Rik			
RP23-133N24.2			
Gata5os			
RP23-298C3.14			
Nxf3	Nuclear RNA export factor 3	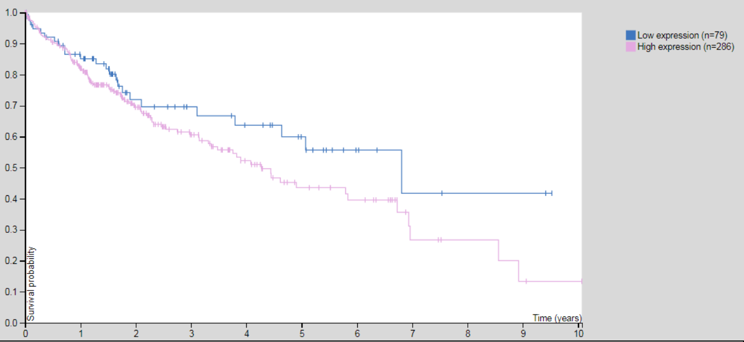	0.12
Rgs5	Regulator of G-protein signaling 5	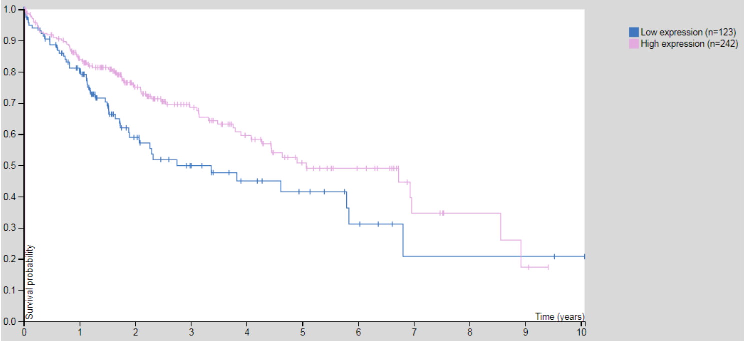	0.017
Gm16226			
Gm43216			
RP23-64D23.6			
Gm11869			
Gm27857			
Gm14435			

**Gdap1l1**	**Ganglioside induced differentiation associated protein 1 like 1**	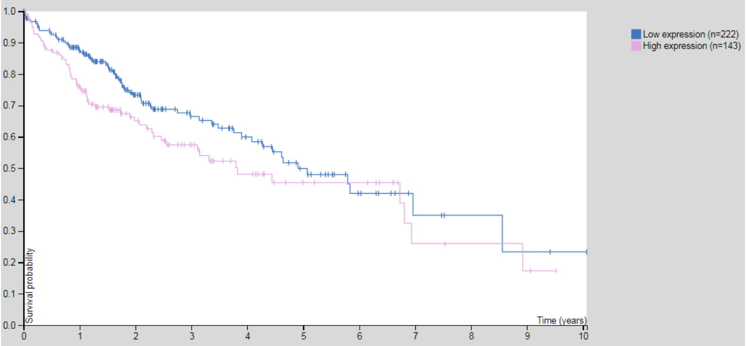	**0.065**
**Cyp4x1**	**Cytochrome P450 family 4 subfamily X member 1**	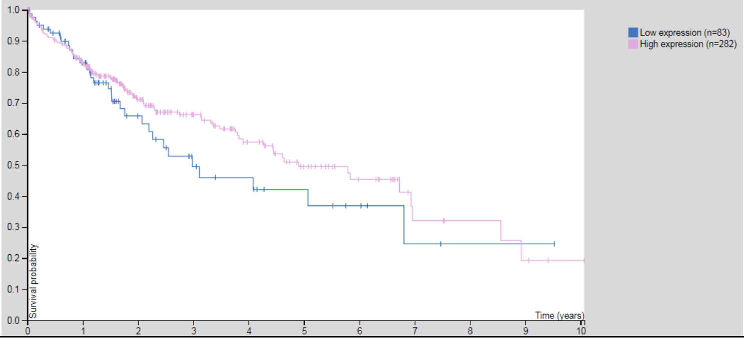	**0.23**
**Gm11453**			
**Kcnk6**	**Potassium two pore domain channel subfamily K member 6**	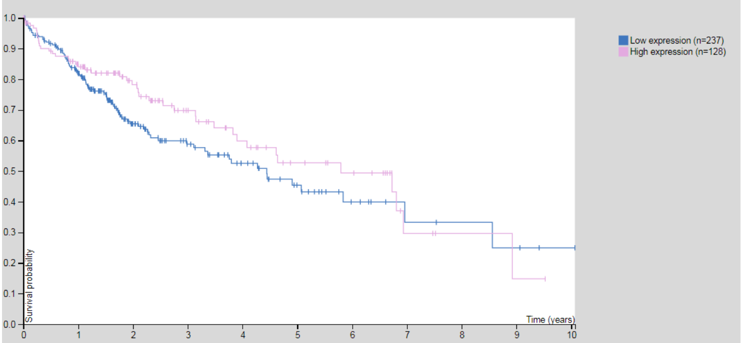	**0.19**
**Sebox**	**SEBOX homeobox**	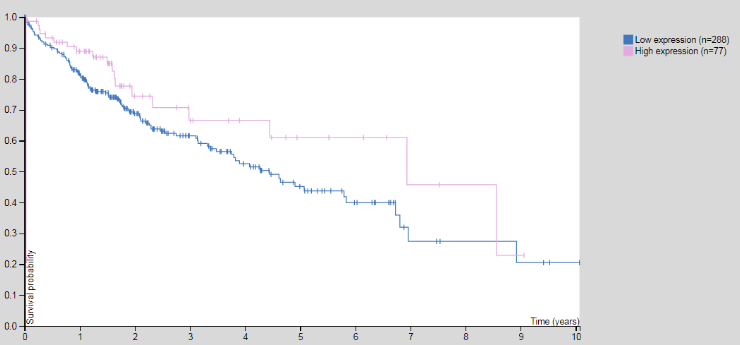	**0.087**
**Fmo2**	**Flavin containing monooxygenase 2**	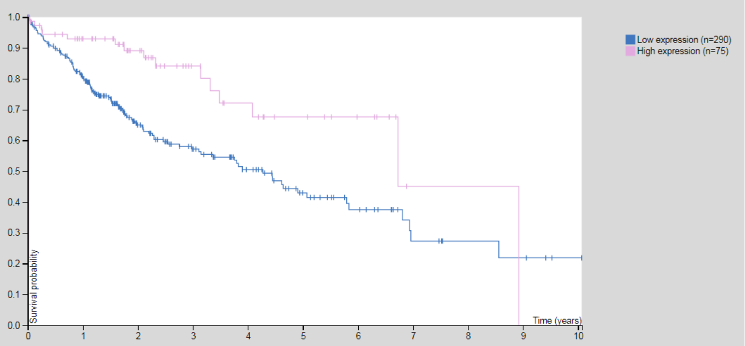	**0.0017**
**Gm9003**			
**Gm26175**			

Gm26353			
Gm22656			
Gm12042			
RP24-127M20.6			
n-R5s27			
Gm19620			
Trdv4	T cell receptor delta variable 4		
Gm13511			
D630029K05Rik			
Gm16581			
Gm38344			
Gm26530			
Rpl21-ps7			
Gm26728			
Mir7662			
Zfp872	zinc finger protein 872		
Gm17281			
Gm30667			
Gm26850			
B230322F03Rik			
Gm37932			
Mir322			
Scrt2	Scratch family transcriptional repressor 2		
Defb1	Defensin beta 1	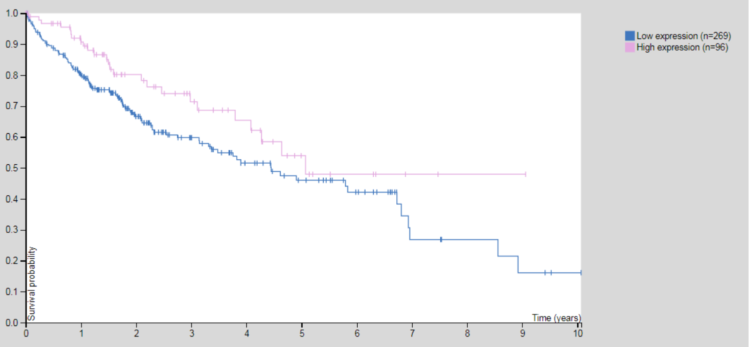	0.044
Lect2	Leukocyte cell derived chemotaxin 2		
Hist2h2bb			
Gm1848			
Asic2	Acid sensing ion channel subunit 2	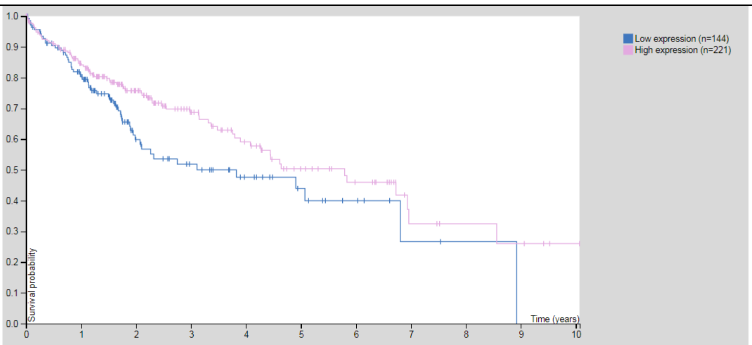	0.049

**Lhx4**	**LIM homeobox 4**	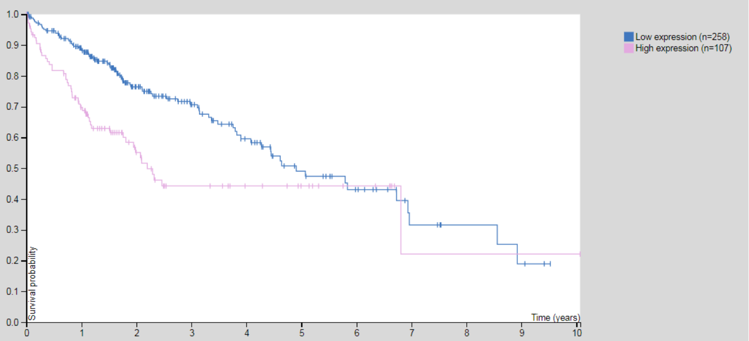	**0.00094**
**Sec14l4**	**SEC14 like lipid binding 4**	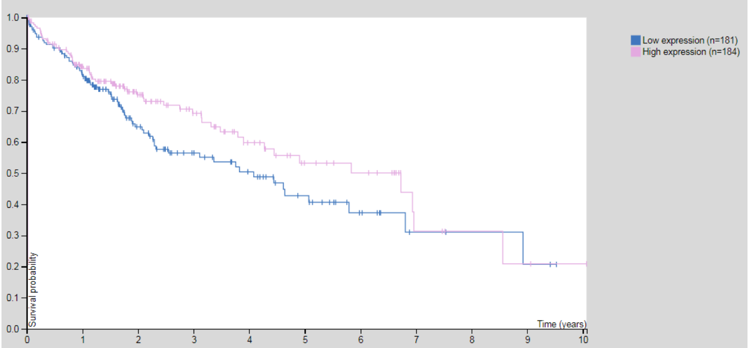	**0.099**
**Hsf5**	**Heat shock transcription factor 5**	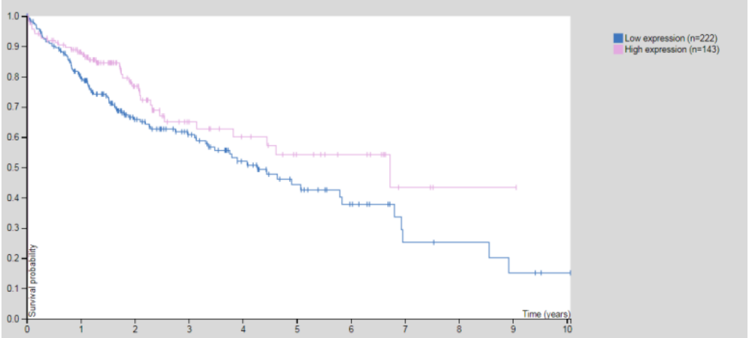	**0.042**
**1700047N06Rik**			
**Celsr3**	**Cadherin EGF LAG seven-pass G-type receptor 3**	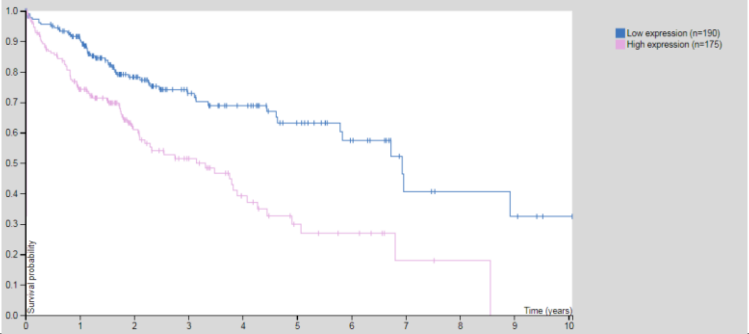	**0.0000036**
**Saa1**	**Serum amyloid A1**	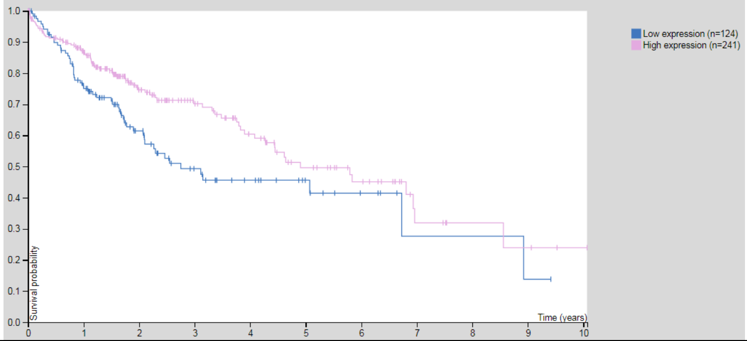	**0.016**
**Gm2147**			
**Gm16416**			

**Gp1ba**	**Glycoprotein Ib platelet alpha subunit**		
**Gm26854**			
**Gm11975**			
**Cpxm2**			
**Adcy4**	**Adenylate cyclase 4**	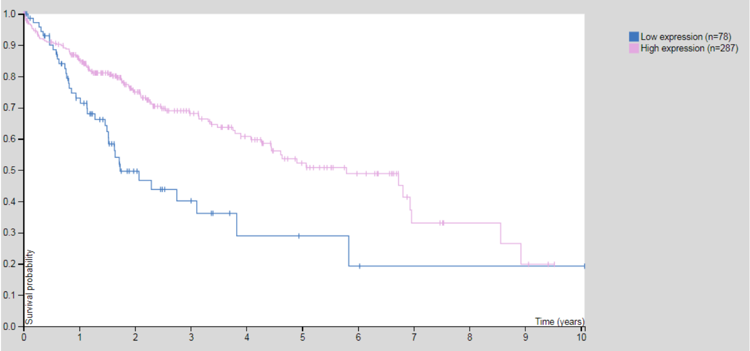	**0.00018**
**Gm43578**			
**Gm23301**			
**Kcnj10**	**Potassium voltage-gated channel subfamily J member 10**	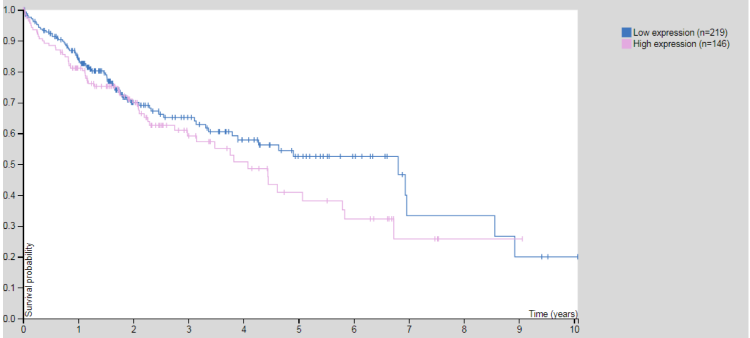	**0.20**
**Fgr**	**FGR proto-oncogene, Src family tyrosine kinase**	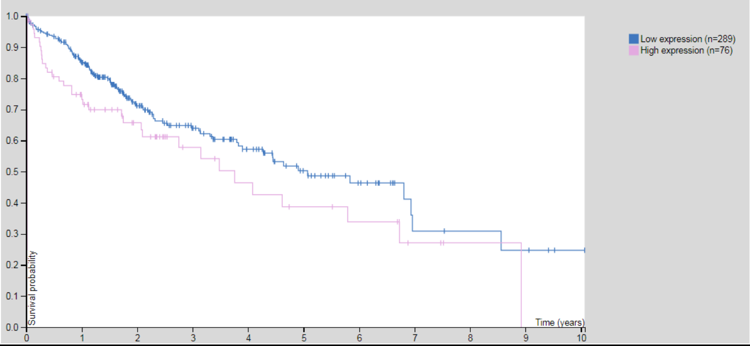	**0.058**
**Gm42503**			
**RP23-296N5.4**			
**Sct**	**Secretin**	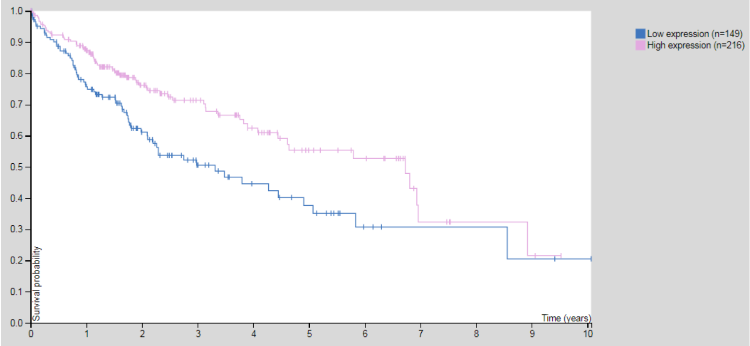	**0.0024**
**Gm6583**			
**Gm2199**			

**Gm43110**			
**Gm43702**			
**RP23-304D1.6**			
**Cilp**	**Cartilage intermediate layer protein**	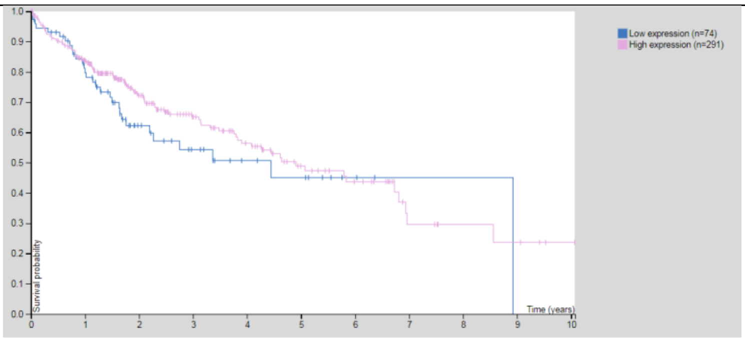	**0.26**
**Gm26394**			
