# Supplementary material for: LGR5 marks targetable tumor-initiating cells in mouse liver cancer
Source: Nat Commun. 2020 Apr 23;11:1961. doi: 10.1038/s41467-020-15846-0 (PMC7181628; doi:10.1038/s41467-020-15846-0)
Supplement: Supplementary file 8 — Supplementary Data 5 [file 41467_2020_15846_MOESM8_ESM.pdf]

Primary Tissue						
Relative Organoid strain	Isolated LGR5$^+$ cell number	Initiated organoid number	Organoid initiated efficiency (%)	Isolated LGR5$^-$ cell number	Initiated organoid number	Organoid initiated efficiency (%)
PT1	0	0	0	1	0	0
PT2	1	0	0	7	0	0
PT3	10	1	10	17	0	0
PT11	307	0	0	8124	0	0
PT12	787	2	0.25	39066	20	0.05
PT13	24	0	0	332457	0	0
PT14	140	0	0	44901	0	0
PT15	58	0	0	186498	0	0
PT16	49	0	0	21632	0	0
PT17	59	0	0	221656	0	0
PT18	51	0	0	185640	0	0
PT19	81	0	0	8985	0	0
PT20	379	1	0.26	26090	75	0.29
PT21	611	1	0.16	13775	39	0.28
PT22	20	0	0	475	0	0
PT23	322	0	0	27748	0	0
PT24	3	0	0	8951	0	0
PT26	32	0	0	87	0	0
PT27	17	0	0	19104	30	0.16
PT28	17	0	0	8643	3	0.035
PT29	62	0	0	10809	22	0.20
PT30	124	0	0	6680	5	0.075
PT31	140	0	0	2692	0	0
PT32	41	0	0	6430	0	0
PT33	141	0	0	23853	10	0.042
PT34	36	0	0	2355	0	0
PT35	540	0	0	26689	0	0
PT36	265	0	0	86774	0	0
PT37	109	0	0	90979	0	0
PT38	1253	10	8.00	131695	0	0
PT39	1073	0	0	250982	0	0
PT40	115	0	0	1329	0	0
PT41	20	0	0	1464	0	0
PT42	181	0	0	1193	0	0
PT43	131	0	0	5998	0	0
PT44	71	0	0	76	0	0
PT45	740	3	0.41	1348	0	0

PT46	22	0	0	1485	0	0
PT47	2304	5	0.22	5356	0	0
PT48	845	1	0.12	859	0	0
PT49	482	1	0.21	418	1	0.24
PT50	385	2	0.52	444	0	0
PT51	140	5	3.57	189	1	0.53
PT52	12	0	0	594	0	0
PT53	1	0	0	180	0	0
PT54	6062	50	0.82	5767	0	0
PT55	407	0	0	2121	0	0
PT56	2282	364	16.0	3841	2	0.05
PT57	288	3	1.04	960	0	0
PT58	120	0	0	1613	0	0
PT59	821	23	2.8	758	0	0
PT60	30	0	0	1299	0	0
PT61	1879	0	0	2242	0	0
PT62	2881	10	3.5	4514	0	0
PT63	3510	27	7.7	1262	0	0
PT64	6	0	0	16	0	0
PT65	340	0	0	281	0	0
PT66	1425	0	0	1222	0	0
PT67	4404	3	0.00068	1026	0	0
PT68	9386	25	2.7	5129	0	0
PT69	93	0	0	128	0	0
PT70	1694	10	0.59	728	0	0
PT71	1526	14	0.92	1792	0	0
PT72	4482	6	0.13	4482	0	0
PT73	1101	4	0.36	1097	0	0
PT80	103	0	0	729	0	0
PT81	58	0	0	51	0	0
PT82	129	0	0	37	0	0
PT83	627	14	2.23	616	0	0
PT84	31	0	0	18	0	0
PT85	652	8	1.2	784	0	0

Allograft Tissue						
Relative Organoid strain	Isolated $LGR5^+$ cell number	Initiated organoid number	Organoid initiated efficiency (%)	Isolated $LGR5^-$ cell number	Initiated organoid number	Organoid initiated efficiency (%)
AL17	70	64	91.4	513	46	9,0
AL13	1026	233	22.7	663	5	0,8
SAL1	7	7	100	38	7	18,4
AL43	39	15	38.5	54	18	33,3
AL46	13	5	38.5	1	0	0,0
AL13.1	1646	180	10.9	1373	176	12,8
AL17.2	21	11	52.4	366	11	3,0
AL8.1	62	17	27.4	100	3	3,0
AL8.2	107	12	11.2	181	7	3,9
AL8.3	138	16	11.6	178	0	0,0
