# Supplementary material for: LGR5 marks targetable tumor-initiating cells in mouse liver cancer
Source: Nat Commun. 2020 Apr 23;11:1961. doi: 10.1038/s41467-020-15846-0 (PMC7181628; doi:10.1038/s41467-020-15846-0)
Supplement: Supplementary file 9 — Supplementary Data 6 [file 41467_2020_15846_MOESM9_ESM.pdf]

	Tissue Code	Injected Cell Type	Injected Cell Number	Tumor initiation
1	PT47	LGR5^{+} cells	3000	no
		LGR5^{-} cells	3000	no
2	PT50	LGR5^{+} cells	2000	SAL1
		LGR5^{-} cells	2000	SAL2
3	PT63	LGR5^{+} cells	2000	SAL3
		LGR5^{-} cells	2000	no
4	PT65	LGR5^{+} cells	6000	no
		LGR5^{-} cells	6000	no
5	PT67	LGR5^{+} cells	1000	no
		LGR5^{-} cells	1000	no
6	PT68	LGR5^{+} cells	16000	SAL4
		LGR5^{-} cells	16000	no
7	PT72	LGR5^{+} cells	5000	no
		LGR5^{-} cells	5000	no
8	PT73	LGR5^{+} cells	1000	no
		LGR5^{-} cells	1000	no
9	PT85	LGR5^{+} cells	3000	no
		LGR5^{-} cells	3000	no

Tissue code	Primary tumor	Allograft Tumor
SAL1	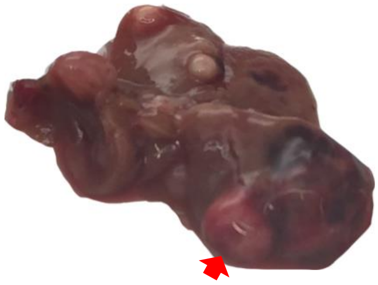	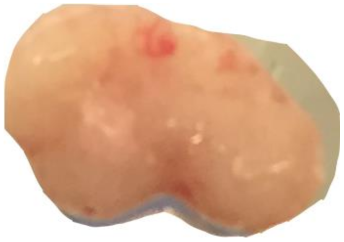
SAL2	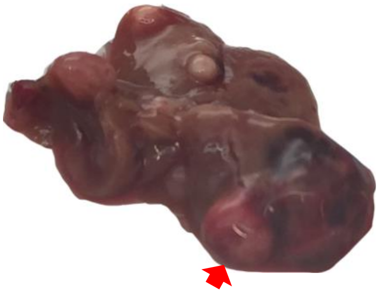	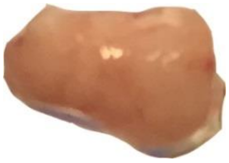
SAL3	Picture lacking	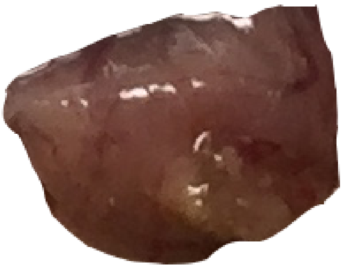
SAL4	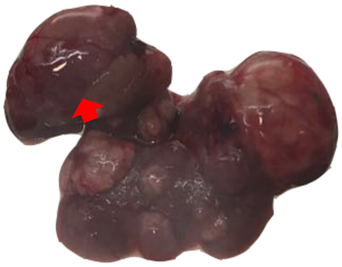	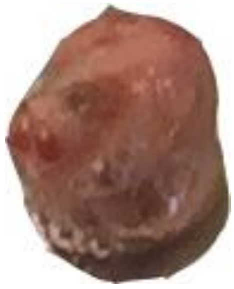
