# Supplementary material for: LGR5 marks targetable tumor-initiating cells in mouse liver cancer
Source: Nat Commun. 2020 Apr 23;11:1961. doi: 10.1038/s41467-020-15846-0 (PMC7181628; doi:10.1038/s41467-020-15846-0)
Supplement: Supplementary file 10 — Reporting summary [file 41467_2020_15846_MOESM10_ESM.pdf]

natureresearch

Corresponding author(s): Qiuwei Pan

Last updated by author(s): Mar 18, 2020

Reporting Summary

Nature Research wishes to improve the reproducibility of the work that we publish. This form provides structure for consistency and transparency in reporting. For further information on Nature Research policies, see Authors & Referees and the Editorial Policy Checklist .

Statistics

For all statistical analyses, confirm that the following items are present in the figure legend, table legend, main text, or Methods section.

n/a	Confirmed	
☐	☒	The exact sample size (*n*) for each experimental group/condition, given as a discrete number and unit of measurement
☐	☒	A statement on whether measurements were taken from distinct samples or whether the same sample was measured repeatedly
☐	☒	The statistical test(s) used AND whether they are one- or two-sided<br>*Only common tests should be described solely by name; describe more complex techniques in the Methods section.*
☐	☒	A description of all covariates tested
☐	☒	A description of any assumptions or corrections, such as tests of normality and adjustment for multiple comparisons
☐	☒	A full description of the statistical parameters including central tendency (e.g. means) or other basic estimates (e.g. regression coefficient) AND variation (e.g. standard deviation) or associated estimates of uncertainty (e.g. confidence intervals)
☐	☒	For null hypothesis testing, the test statistic (e.g. *F*, *t*, *r*) with confidence intervals, effect sizes, degrees of freedom and *P* value noted<br>*Give P values as exact values whenever suitable.*
☒	☐	For Bayesian analysis, information on the choice of priors and Markov chain Monte Carlo settings
☒	☐	For hierarchical and complex designs, identification of the appropriate level for tests and full reporting of outcomes
☒	☐	Estimates of effect sizes (e.g. Cohen's *d*, Pearson's *r*), indicating how they were calculated

Our web collection on statistics for biologists contains articles on many of the points above.

Software and code

Policy information about availability of computer code

Data collection	No software was used
Data analysis	No software was used

For manuscripts utilizing custom algorithms or software that are central to the research but not yet described in published literature, software must be made available to editors/reviewers. We strongly encourage code deposition in a community repository (e.g. GitHub). See the Nature Research guidelines for submitting code & software for further information.

Data

Policy information about availability of data

All manuscripts must include a data availability statement. This statement should provide the following information, where applicable:

- Accession codes, unique identifiers, or web links for publicly available datasets
- A list of figures that have associated raw data
- A description of any restrictions on data availability

The RNA-seq data have been deposited in the GEO database under the accession code GSE137517 . The human LGR5 expression and HCC mutation data referenced during the study are available in a public repository from the TCGA and LICA database. The source data underlying Figs. 1c-d, 2, 3e, 4d, f, h, i, p, 5b, f, h-i, 6c, h, I, 7b, c, f, g, 8b, c, f, g and Supplementary Figs. 2a, g, 3, 4, 6b-c, 7b-e, 8a-b, 9, 10c-d, 11, 12a-b and 13g-h are provided as a Source Data file. All the other data supporting the findings of this study are available within the article and its supplementary information files and from the corresponding author upon reasonable request. A reporting summary for this article is available as a Supplementary Information file.

Field-specific reporting

Please select the one below that is the best fit for your research. If you are not sure, read the appropriate sections before making your selection.

- [x] Life sciences
- [ ] Behavioural & social sciences
- [ ] Ecological, evolutionary & environmental sciences

For a reference copy of the document with all sections, see nature.com/documents/nr-reporting-summary-flat.pdf

Life sciences study design

All studies must disclose on these points even when the disclosure is negative.

Sample size	Sample size is determined according to precious study.
Data exclusions	No data exclusions.
Replication	All the measurements are replicated. The N is indicated in the manuscript.
Randomization	Not relevant to the study
Blinding	Not relevant to the study

Reporting for specific materials, systems and methods

We require information from authors about some types of materials, experimental systems and methods used in many studies. Here, indicate whether each material, system or method listed is relevant to your study. If you are not sure if a list item applies to your research, read the appropriate section before selecting a response.

Materials & experimental systems

n/a	Involved in the study	
☐	☒	Antibodies
☒	☐	Eukaryotic cell lines
☒	☐	Palaeontology
☐	☒	Animals and other organisms
☒	☐	Human research participants
☒	☐	Clinical data

Methods

n/a	Involved in the study	
☒	☐	ChIP-seq
☐	☒	Flow cytometry
☒	☐	MRI-based neuroimaging

Antibodies

Antibodies used

CD45(56-0451-82,eBioscience)
AFP(SAB3500533-100UG,Sigma)
HNF4a(ab41898,Abcam); EpCAM (ab71916, Abcam)
Cytokeratin 19(ab52625,Abcam)
GFP(A-11122; Invitrogen/Life Technologies)
Alexa Fluor® 488 AffiniPure Donkey Anti-Goat IgG (H+L) (705-545-147,Bio-Connect)
Donkey anti-Rabbit IgG (H+L) Secondary Antibody Alexa Fluor® 594 conjugate(R37119,Thermo fisher)
Donkey anti-mouse IgG (H+L) Secondary Antibody, Alexa Fluor® 594 conjugate(R37115, Thermo fisher)

Validation

CD45: mouse, expel immune cells
AFP: mouse, hepatocyte/HCC straining
HNF4a: mouse, hepatocyte/HCC straining
EpCAM: mouse, indicate cholangiocyte/CC/CHC
Cytokeratin 19: mouse, indicate cholangiocyte/CC/CHC
GFP: mouse, indicate LGR5-GFP
Alexa Fluor® 488 AffiniPure Donkey Anti-Goat IgG (H+L) : mouse, secondary antibody
Donkey anti-Rabbit IgG (H+L) Secondary Antibody, Alexa Fluor® 594 conjugate: mouse, secondary antibody
Donkey anti-mouse IgG (H+L) Secondary Antibody, Alexa Fluor® 594 conjugate: mouse, secondary antibody

Animals and other organisms

Policy information about studies involving animals; ARRIVE guidelines recommended for reporting animal research

Laboratory animals	NOD.Cg-PrkdcSCIDIl2rgtm1Wjl/SzJ (NSG) mice: Female, Week 6-40<br>NOG/JicTac (CIEA NOD.Cg-Prkdc-scid Il2rg-tm1Sug) mice: Female, Week 6-40<br>Nude mice (NMRI:BomTac-Nude): Female, Week 6-40<br>LGR5-DTRGFP: Female/Male, Week 6-76
Wild animals	This study did not involve wide type animals.
Field-collected samples	This study did not involve samples collected from field.
Ethics oversight	The Committee on the Ethics of Animal Experiments of the Erasmus Medical Center

Note that full information on the approval of the study protocol must also be provided in the manuscript.

Flow Cytometry

Plots

Confirm that:

- [x] The axis labels state the marker and fluorochrome used (e.g. CD4-FITC).
- [x] The axis scales are clearly visible. Include numbers along axes only for bottom left plot of group (a 'group' is an analysis of identical markers).
- [x] All plots are contour plots with outliers or pseudocolor plots.
- [x] A numerical value for number of cells or percentage (with statistics) is provided.

Methodology

Sample preparation	For FACS analysis, single cells derived from liver tumors/tumor surrounding tissues or organoids were suspended in DMEM plus 2% FBS. Cell suspensions were analyzed
Instrument	BD FACSAriaTM II
Software	BD FACSDIVA Software
Cell population abundance	The collected cells were re-measured to check the abundance.
Gating strategy	A figure exemplifying the gating strategy is provided in the Supplementary Information

- [x] Tick this box to confirm that a figure exemplifying the gating strategy is provided in the Supplementary Information.
