# Supplementary material for: LGR5 marks targetable tumor-initiating cells in mouse liver cancer
Source: Nat Commun. 2020 Apr 23;11:1961. doi: 10.1038/s41467-020-15846-0 (PMC7181628; doi:10.1038/s41467-020-15846-0)
Supplement: Supplementary file 2 — Peer Review File [file 41467_2020_15846_MOESM2_ESM.pdf]

Editorial Note: The figure on page 15 in this Peer Review File has been removed due to containing third-party material where no permission to publish could be obtained.

Reviewers' comments:

Reviewer #1; experert in cancer stem cell, organoids

In this paper from Cao et al the authors aimed to investigate whether the well known intestinal stem cell marker LGR5 also marks cancer stem cells or tumour-initiating cells in liver cancer, and to explore the potential for therapeutic targeting of these cells.

The manuscript conceptual advance and novelty resides in the second part, where the authors describe the role of Lgr5+ cells in liver cancer resistance to conventional treatments. Yet, the first part of this manuscript suffers from some conceptual flaws that would require re-analysis of the data and maybe revision of the conclusions drawn, see comments below. In addition, reg the most interesting observation, the appearance of a Lgr5+ resistant population on the tumours, most of the work is done in the organoids, and the model used (DEN model), is a mutagenesis model that is not clear what type of human tumour recapitulates.

My comments below:
1) In the first part of the manuscript the authors test if Lgr5 would be expressed in the DEN mouse liver cancer model the authors take advantage of the Lgr5-DTR-GFP mouse, which is a faithful reporter of Lgr5 expression. It is interesting to see that indeed, the stem cell marker Lgr5 gets induced in liver tumours in this model. Also in the tumour compared to surrounding. Several questions arise from this first part of the data:
Also, one thing that is not clear is how many animals got tumours and how many of these were Lgr5+? In Suppl Fig 2 the authors mention the fraction of tumours that harbour a certain % of Lgr5+ cells but this does not give us an idea of the penentrance, in other words, how many of the mice developed tumours and how many of these contained Lgr5+ cells and which fraction of it? In addition, I find the statistics a bit unbalanced, as the authors use very different numbers of sample size. In Fig 1 legend, the authors mention the n (I assume number of animals, this is not specified) as n=87 (DEN tumour) vs n=4 (undamaged) vs n=7 (damaged). Is these 87 animals with tumours, or tumours in a certain number of animals? I understand that they cannot compare tumours vs animals that do not have tumours, but then in that case the data should be presented maybe as samples analysed, in that case, then they can balance the numbers to get similar group sizes. Statistics on such a big difference in group sizes is not recommended as it causes false positives very easily. .

The same comment as above accounts for the comparison between tumours (n=54 vs n=33) and surrounding tissue in Fig 1d. In that case, indeed they should be able to have a balanced group size since both samples should be coming from the same tissue/animal. Comparing such a big difference in group sizes is statistically incorrect. Specially taking into account that the authors use mean comparison with Mann-Whitney test for that, which would come with the assumption that both groups are at least similar size. Since the disparity of the samples here is not that much the authors could compensate for that using a Welch test, instead.

In addition, what time point are these mice analysed? In the DEN model (Suppl Fig 2) they observe Lgr5+ cells already at 7 days. But authors mention that it takes a range of months, 4-14 months to get tumours in the DEN model. So, in Fig 1c, where they compare normal vs damage vs tumour, what time point are these collected? Is it the same for the 3 groups? In addition, it would be useful to know the number of tumours generated per timepoint per mice. Maybe a suppl table could be included for that.
To confirm the Flow and IF analysis data, the authors generate organoids from these DEN-induced tumours and implant them in NSG mice after which they retrieve organoids again and test the expression of Lgr5. The experiments indeed show that the tumours can initiate organoid cultures. This data confirms the experiments in Fig 1. and the claim that the tumours contain Lgr5+ cells.

2) Next the authors compare the tumorogenic capacity of Lgr5+ vs Lgr5- cells. For that they

perform 2 types of experiments, on one hand, sort the cells directly from the tumour and initiate organoid cultures and in a second step they use the grown organoids to generate tumours in the mouse. In this section the authors conclude that Lgr5+ tumour cells initiate more colonies than the non Lgr5+ ones. Yet, when looking at the sorting strategy, one realizes that the authors are sorting only from GFP + vs GFP-. This poses a clear caveat to this experiment, because the authors are comparing Lgr5+ epithelial cells vs Lgr5- cells in the tumour, which will contain not only epithelial but also mesenchymal and/or endothelial cell. This is an unfair comparison between the two populations and while it does not invalidate that Lgr5+ cells inititate tumours it does not demonstrate that the Lgr5+ cells are more tumorigenic than the Lgr5-. So this claim and conclusion is not supported by the data.

3) In the second part of the manuscript the authors compare the expression of Lgr5+ before and after treatment with anticancer drugs 5FU and Sorafenib. And combined ablation of Lgr5+ cells with different anticancer drugs. The data suggests that these treatments enhance the number of Lgr5+ cells and hence ablation of the Lgr5+ compartment could represent a good therapeutic strategy for liver cancer. This is potentially an interesting observation as it could have clinical implications for the management of liver cancer patients. Yet this part would require further work in order to get to these more big conclusions. Some points here:

- are these drug-induced Lgr5+ cells the same as the ones from the non-treated tumour in terms of tumorogenic and clonogenic potential? Can they sustain organoids long term and initate de novo organoids? Can these organoids generate the novo tumours?

-the authors mention that ablation of the Lgr5+ tumour cells kills the tumour organoids but not the control non tumour ones. They refer to their previous in Gastroenterology publication for that . Yet this data is not shown. Also, how they reconcile both somehow contradictory pieces of data?

-In addition, since this work is only perfomed in organoids or transplanted organoids that are treated after transplant, one cannot evaluate whether this resistance would also account for in vivo resistance. In other words, if they treat DEN-induced tumours with these agents, do they get resistance and is this started from Lgr5+ cells?

Minor points:

1) Supl Fig 1 gives the numbers of mice but there is no legend. What is S and T?
2) in suppl Fig 4h, it would be more compelling if the authors show that the cultures start from single cells, instead of showing 1 organoids at later stage

Reviewer #2; expert in liver cancer mouse models

The here proposed manuscript by Wanlu et al. investigated the role of LGR5 (leucine-rich repeat-containing G protein-coupled receptor 5) in murine liver tumor initiation, progression and chemoresistance. They identified LGR5 as a potential marker for cancer stem cells/tumor initiating cells (CSC/TICs) and investigated in vitro and in vivo the tumorigenicity of LGR5+ cells/allograft tumors. They further analyzed the potential of targeting LGR5+ cell populations in murine primary liver tumors as a novel therapeutic strategy for liver cancer.

Key findings of the study:
• Primary murine liver tumors were enriched in LGR5+ cells compared to normal or injured liver.
• Cultured organoids of primary murine liver tumors conserved tumorigenic potential and contained LGR5+ cells.
• LGR5+ cells in comparison to LGR5- cells were superior in organoid and tumor initiation.

• LGR5+ cells were enriched during anti-cancer treatment by 5-FU or sorafenib.
• LGR5 lineage ablation inhibited organoid and tumor growth similar to 5-FU and sorafenib treatment.
• Combination of LGR5-depletion and 5-FU treatment resulted in a more significant tumor reduction as monotherapy strategies.

Overall, the authors investigate a relevant and timely topic, namely the role of LGR5 for cancer stemness. The study clearly established LGR5 as a marker for putative murine liver CSCs. The is manuscript well written and technically sound. Major concerns relate to a missing translation into authentic human tumors. Furthermore, more mechanistic investigations into regulatory effects of LGR5 positive putative CSC seem important.

Major comments:
• Major concerns relate to the missing translation into human disease. It would be crucial to investigate LGR5-expression in authentic human samples from different etiologies. The reviewer acknowledges the lack of suitable human antibodies. However, transcriptome expression could be assessed. Furthermore, prognostic implications should be determined (e.g. by using publically available data).In line with this, mechanistic investigations on LGR5 positive and negative putative CSCs should be performed to gain insights into the regulatory network of the cells (e.g. by RNA seq). Transcriptome profiles should also be evaluated in the context of the human disease. Of note, in addition to CTNNB1 mutations DEN is known to induce BRAF mutations, an alteration that is not frequently observed in human HCC. It would be interesting to investigate if the mutational status impairs the LGR5 expression of the tumors (publically available data could be explored)?
• The authors imply that the presence of CK19/HNF4a and LGR5 positive cells could result in both HCC as well as CCA development (page 7). This suggested bipotentiality is not underlined by the presented data. Please show representative IHC pictures demonstrating cholangiocellular differentiation in the model or provide further experimental evidence (e.g. by transplantation). In line with this, authors demonstrate that there are two different organoid-types in culture – a HCC and a CCC-like organoid type, reflected by co-expression of HNF4a and CK19. However, in further analyses different types are not distinguished. It would be important to assess the ratio between HCC and CCA organoids? Do the different organoids show distinct populations of LGR5 positive cells (frequency should be provided)? Is there a selection of phenotypes that is favored by the culturing or is e.g. CK19 expression induced by the artificial culture conditions (are other lineage markers expressed as well)? Do different types have the same or different tumor initiating capacity when transplanted into mice? And is there any difference in response to treatment comparing tumors derived from HCC-like organoids compared to CCC-like organoids? In addition, it is quite surprising that de-differentiated tumors express high levels of HNF4a. Please comment (what is the differentiation status of the tumors?).
• Depletion of LGR5 inhibited tumor growth similar to sorafenib and 5-FU treatment (Fig. 5i). It would be interesting to perform combination therapy of both compounds with LGR5-depletion to confirm potential synergistic effects (Fig 6). How was the number of transplanted cells controlled in the treatment experiments (Fig 4)?
• Induction of LGR5 positive cells from LGR5 negative cells implicates plasticity of LGR5 putative CSCs. This might significantly affect the therapeutic potential (page 15). This should be mentioned and discussed. Given the role of CSC for tumor development, it would be interesting to dissect whether LGR5 depletion by diphtheria toxin would impair tumor development or just progression. Thus, it would be interesting to test this in the DEN model. However, the reviewer acknowledges that this might be beyond the scope of the here presented manuscript.

Minor comment:
• CCL4 treatment is not described. Please also provide a suitable reference for the DEN protocol applied.
• Figure 2e demonstrate only 2 allograft tumors highly positive for LGR5 cells after organoid transplantation. Further, comparison to primary tumors is not significant (p=0.3577). How many organoids retained LGR5+ cells? Is there a significant association for successful allograft

transplantation? More details should to be provided.

- Please note that FDA approved lenvatinib for first line treatment of HCC (page 14). Furthermore, regorafenib, cabozantinib, pembrolizumab as well as nivolumab are approved for 2nd line. Thus, sorafenib is not the only approved FDA drug, but still the standard of care. This should be corrected.
- Magnification should be provided for all IHC/IF images

<u>Response to Reviewer #1:</u>

<u>Major comments:</u>

<u>Question 1:</u> *<u>One thing that is not clear is how many animals got tumours and how many of these were Lgr5+? In Suppl Fig 2 the authors mention the fraction of tumours that harbour a certain % of Lgr5+ cells but this does not give us an idea of the penentrance, in other words, how many of the mice developed tumours and how many of these contained Lgr5+ cells and which fraction of it? In addition, I find the statistics a bit unbalanced, as the authors use very different numbers of sample size. In Fig 1 legend, the authors mention the n (I assume number of animals, this is not specified) as n=87 (DEN tumour) vs n=4 (undamaged) vs n=7 (damaged). Is these 87 animals with tumours, or tumours in a certain number of animals? I understand that they cannot compare tumours vs animals that do not have tumours, but then in that case the data should be presented maybe as samples analysed, in that case, then they can balance the numbers to get similar group sizes. Statistics on such a big difference in group sizes is not recommended as it causes false positives very easily. The same comment as above accounts for the comparison between tumours (n=54 vs n=33) and surrounding tissue in Fig 1d. In that case, indeed they should be able to have a balanced group size since both samples should be coming from the same tissue/animal. Comparing such a big difference in group sizes is statistically incorrect. Specially taking into account that the authors use mean comparison with Mann-Whitney test for that, which would come with the assumption that both groups are at least similar size. Since the disparity of the samples here is not that much the authors could compensate for that using a Welch test, instead.</u>*

Answer: We thank the reviewer for his/her interest and constructive comments to our study. We agree that the detailed explanation about the initiated tumor number and corresponding expression of LGR5 is important. Thus, all those information now has been added and presented in the **Supplementary File. 1** and the summary is also added in the manuscript (**Page 19: Line 385-387**).

For the group size and statistical method, we agree with reviewer that there exists a big difference in group sizes. Thus, we have enlarged the healthy and injury group,

although the numbers remain smaller than the tumor group (because this is the most important group). In the tumor group, we have now only included the tumor but not the tumor surrounding tissues (**Fig. 1c**). As suggested, we also changed the statistical method from Mann-Whitney test to Welch test for **Fig. 1c** and **1d**.

Question 2: *In addition, what time point are these mice analysed? In the DEN model (Suppl Fig 2) they observe Lgr5+ cells already at 7 days. But authors mention that it takes a range of months, 4-14 months to get tumours in the DEN model. So, in Fig 1c, where they compare normal vs damage vs tumour, what time point are these collected? Is it the same for the 3 groups? In addition, it would be useful to know the number of tumours generated per timepoint per mice. Maybe a suppl table could be included for that. To confirm the Flow and IF analysis data, the authors generate organoids from these DEN-induced tumours and implant them in NSG mice after which they retrieve organoids again and test the expression of Lgr5. The experiments indeed show that the tumours can initiate organoid cultures. This data confirms the experiments in Fig 1. and the claim that the tumours contain Lgr5+ cells.*

Answer: For the DEN induced mice, the detailed tumor collection time (counted since the first administration of DEN) is listed in the **Supplementary File. 1** (Post Den Time); For the normal livers, the livers were collected at a random age of mice (between Week 8-76); For injured liver, the livers were collected post injury month 1-4. In addition, the number of tumors collected per time point per mice and the corresponding LGR5 expression is also analyzed and presented in **Supplementary Fig. 2g-h**.

Question 3: *Next the authors compare the tumorogenic capacity of Lgr5+ vs Lgr5- cells. For that they perform 2 types of experiments, on one hand, sort the cells directly from the tumour and initiate organoid cultures and in a second step they use the grown organoids to generate tumours in the mouse. In this section the authors conclude that Lgr5+ tumour cells initiate more colonies than the non Lgr5+ ones. Yet,*

when looking at the sorting strategy, one realizes that the authors are sorting only from GFP + vs GFP-. This poses a clear caveat to this experiment, because the authors are comparing Lgr5+ epithelial cells vs Lgr5- cells in the tumour, which will contain not only epithelial but also mesenchymal and/or endothelial cell. This is an unfair comparison between the two populations and while it does not invalidate that Lgr5+ cells inititate tumours it does not demonstrate that the Lgr5+ cells are more tumorigenic than the Lgr5-. So this claim and conclusion is not supported by the data.

Answer: We agree with the reviewer that the GFP^- population may contain other cell populations. Thus, we followed the suggestion and used the PDGFRα and CD31 markers to indicate the mesenchymal and endothelial cell populations, respectively (**Response Letter Figure. 1a**). Immune cells were already excluded in our original FACS analysis. By analyzing eight tumor samples, we found the average percentage for mesenchymal and endothelial cells together is around 5% (**Response Letter Figure. 1b**). We use this percentage to adjust the GFP^- cell number for our previous organoid initiation results (**Response Letter Table. 1**). We observed that the $LGR5^+$ population retains a significantly stronger organoid initiation ability compared to the $LGR5^-$ population (**Response Letter Figure. 1c-d**). Furthermore, we compared the tumor initiation ability for $LGR5^+$ and $LGR5^-$ which were isolated with the new FACS strategy. $LGR5^+$ still show stronger tumor formation ability compared to $LGR5^-$ cells (**Response Letter Figure. 1e**). In addition, we also compared the $LGR5^-$ cells which were separately isolated by the original FACS strategy (excluding immune cells) and the new FACS strategy (excluding immune, mesenchymal and endothelial cells). Those two $LGR5^-$ populations showed similar tumor formation ability (**Response Letter Figure. 1f**). Thus, these results again confirmed that $LGR5^+$ cells are superior in organoid and tumor formation.

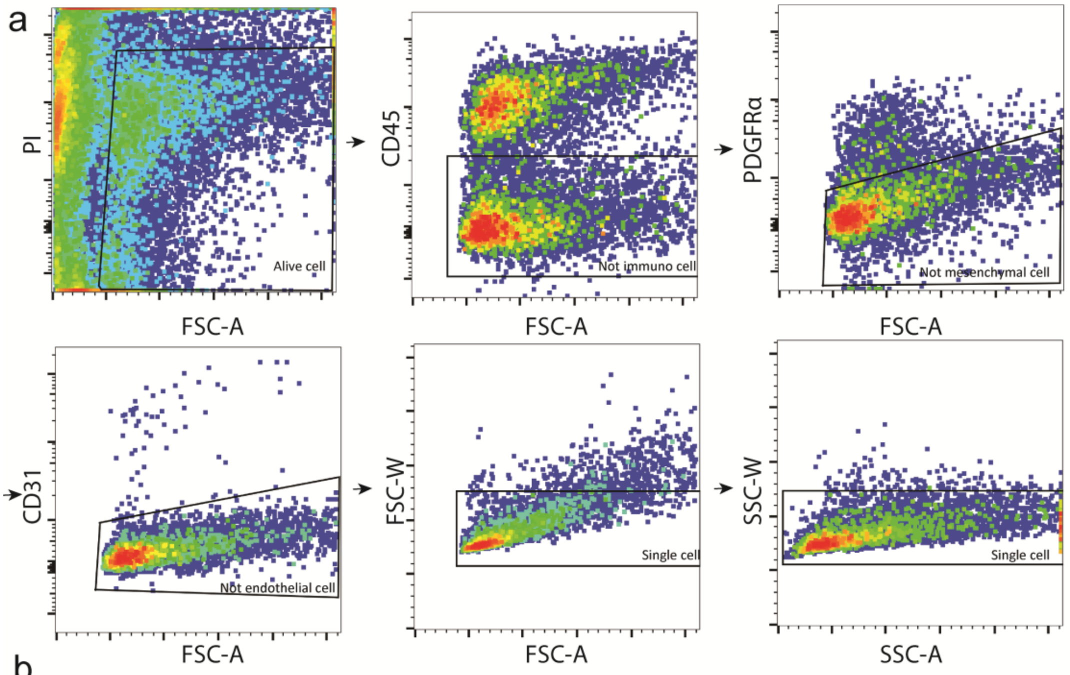

b

Sample Code	The total cell number	The fibroblast cell number	The vescular cell number	Percentage of the fibroblast cells	Percentage of the vascular cells	The total percentage for fibroblast and vascular cells (%)
1	50000	900	66	1,800	0,87	2,670
2	50000	1102	69	2,204	1,02	3,226
3	50000	1444	95	2,888	1,09	3,973
4	67200	3524	166	5,244	1,51	6,757
5	30525	1063	83	3,482	2,97	6,454
6	100000	2067	132	2,067	1,87	3,935
7	100000	1545	151	1,545	1,17	2,715
8	100000	3023	734	3,023	6,16	9,188
Average						4,865

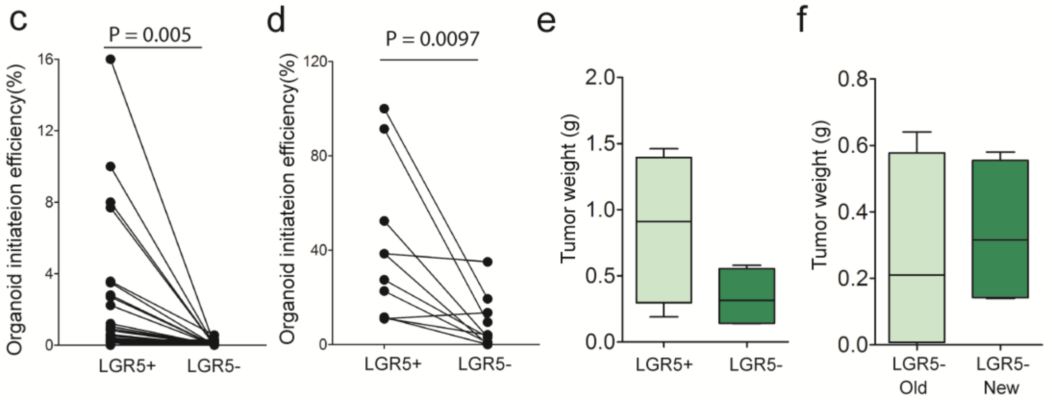

Response Letter Figure 1 | The new FACS sorting strategy. a, The new FACS sorting strategy. **b**, The number and percentage of mesenchymal and endothelial cells for individual tumors. **c**, The organoid initiation efficiency of $LGR5^+$ cells and adjusted $LGR5^-$ cell population which isolate from primary tumors. **d**, The organoid initiation efficiency of $LGR5^+$ cells and adjusted $LGR5^-$ cells which isolate from allograft tumors. **e**, The tumor initiation ability of $LGR5^+$ cells (0.88 ± 0.29g, n = 4) and $LGR5^-$ cells (0.33 ± 0.11g, n = 4) which isolated according to the new FACS sorting strategy. **f**, The tumor formation ability for $LGR5^-$ cells (isolated according to the original FACS sorting strategy) (0.27 ± 0.15g, n = 4) and $LGR5^-$ cells (isolated according to new FACS sorting strategy) (0.33 ± 0.11g, n = 4).

Primary Tissue

Relative Organoid strain	Isolated $Lgr5^+$ cell number	Initiated organoid number	Organoid initiated efficiency (%)	Isolated $Lgr5^-$ cell number	Adjusted lgr5- number	Initiated organoid number	Adjusted Organoid initiated efficiency (%)	Organoid initiated efficiency (%)
PT3	10	1	10	17	16,17	0	0,00	0,00
PT12	787	2	0.25	39066	37151,77	20	0,05	0,05
PT20	379	1	0.26	26090	24811,59	75	0,30	0,29
PT21	611	1	0.16	13775	13100,03	39	0,30	0,28
PT27	17	0	0	19104	18167,90	30	0,17	0,16
PT28	17	0	0	8643	8219,49	3	0,04	0,03
PT29	62	0	0	10809	10279,36	22	0,21	0,20
PT30	124	0	0	6680	6352,68	5	0,08	0,07
PT33	141	0	0	23853	22684,20	10	0,04	0,04
PT38	1253	10	8.00	131695	125241,95	0	0,00	0,00
PT45	740	3	0.41	1348	1281,95	0	0,00	0,00
PT47	2304	5	0.22	5356	5093,56	0	0,00	0,00
PT48	845	1	0.12	859	816,91	0	0,00	0,00
PT49	482	1	0.21	418	397,52	1	0,25	0,24
PT50	385	2	0.52	444	422,24	0	0,00	0,00
PT51	140	5	3.57	189	179,74	1	0,56	0,53
PT54	6062	50	0.82	5767	5484,42	0	0,00	0,00
PT56	2282	364	16.0	3841	3652,79	2	0,05	0,05
PT57	288	3 tiny	1.04	960	912,96	0	0,00	0,00
PT59	821	23	2.8	758	720,86	0	0,00	0,00
PT62	2881	10	3.5	4514	4292,81	0	0,00	0,00
PT63	3510	27	7.7	1262	1200,16	0	0,00	0,00
PT67	4404	3	0.00068	1026	975,73	0	0,00	0,00
PT68	9386	25	2.7	5129	4877,68	0	0,00	0,00
PT70	1694	10	0.59	728	692,33	0	0,00	0,00
PT71	1526	14	0.92	1792	1704,19	0	0,00	0,00
PT72	4482	6	0.13	4482	4262,38	0	0,00	0,00
PT73	1101	4	0.36	1097	1043,25	0	0,00	0,00
PT83	627	14	2.23	616	585,82	0	0,00	0,00
PT85	652	8	1.2	784	745,58	0	0,00	0,00

Allograft Tissue

Relative Organoid strain	Isolated $Lgr5^+$ cell number	Initiated organoid number	Organoid initiated efficiency (%)	Isolated $Lgr5^-$ cell number	Adjusted lgr5- number	Initiated organoid number	Adjusted Organoid initiated efficiency (%)	Organoid initiated efficiency (%)
AL17	70	64	91.4	513	487,86	46	9,43	8,97
AL13	1026	233	22.7	663	630,51	5	0,79	0,75
SAL1	7	7	100	38	36,14	7	19,37	18,42
AL43	39	15	38.5	54	51,35	18	35,05	33,33
AL46	13	5	38.5	1	0,95	0	0,00	0,00
AL13.1	1646	180	10.9	1373	1305,72	176	13,48	12,82
AL17.2	21	11	52.4	366	348,07	11	3,16	3,01
AL8.1	62	17	27.4	100	95,10	3	3,15	3,00
AL8.2	107	12	11.2	181	172,13	7	4,07	3,87
AL8.3	138	16	11.6	178	169,28	0	0,00	0,00

Response letter Table. 1 | The adjusted GFP^- cell number and organoid initiation ability for our precious organoid initiation results (For the groups which initiated organoids previously).

Question 4: *In the second part of the manuscript the authors compare the expression of Lgr5+ before and after treatment with anticancer drugs 5FU and Sorafenib. And combined ablation of Lgr5+ cells with different anticancer drugs. The*

data suggests that these treatments enhance the number of Lgr5+ cells and hence ablation of the Lgr5+ compartment could represent a good therapeutic strategy for liver cancer. This is potentially an interesting observation as it could have clinical implications for the management of liver cancer patients. Yet this part would require further work in order to get to these more big conclusions. Some points here: - are these drug-induced Lgr5+ cells the same as the ones from the non-treated tumour in terms of tumorogenic and clonogenic potential? Can they sustain organoids long term and initate de novo organoids? Can these organoids generate the novo tumours?

Answer: To investigate the tumorigenic and clonogenic potential for drug treated LGR5 expressing cells, we carried-out the corresponding experiments. The new results are presented in **Supplementary Fig. 8a-d**. We observed that 5-FU treated $LGR5^+$ cells remain capable of organoid and tumor initiation, compared to untreated $LGR5^+$ cells. Those 5-FU treated $LGR5^+$ cells initiated organoids can be weekly passaged for more than 2 months (up to now). Interestingly, we also observed that 5-FU treated $LGR5^-$ cells showed enhanced tumor initiation ability. Thus, we further performed RNA-sequencing to compare the difference between 5-FU treated/untreated $LGR5^+$ and $LGR5^-$ cells. The corresponding results are added in **Supplementary Fig. 9a-c and Supplementary File.3**. The related description is also added in manuscript (**Page 11-12, Line 221-227**).

Question 5: *the authors mention that ablation of the Lgr5+ tumour cells kills the tumour organoids but not the control non tumour ones. They refer to their previous in Gastroenterology publication for that. Yet this data is not shown. Also, how they reconcile both somehow contradictory pieces of data?*

Answer: We agree with the reviewer that we should explain this more clearly for the control organoids. The control organoid in this manuscript is the tumor organoid strain derived from DEN induced wild type mice with tumor initiation ability as presented in **Supplementary Fig. 12**. DT treatment did not influence their growth

(**Fig. 6c: left panel**) and tumor initiation (**Supplementary Fig. 12)** of these wild type tumor organoids. This is different from our previous Gastroenterology paper (this only contains normal but not tumor organoids). We referred to this previous study to explain why these concentrations of DT were used (**Page 12, Line 235-237, 241 and Page 19, Line 380-381)**.

Question 6: *In addition, since this work is only perfomed in organoids or transplanted organoids that are treated after transplant, one cannot evaluate whether this resistance would also account for in vivo resistance. In other words, if they treat DEN-induced tumours with these agents, do they get resistance and is this started from Lgr5+ cells?*

Answer: Indeed, we used organoids in culture and allograft (subcutaneous) tumor model in mice, but not the DEN-induced primary liver tumor model in mice. The first main reason is that DEN-induced liver tumor formation in mice varies extensively in terms of time of tumor initiation, and tumor size and number, and we are not able to track these processes without sacrificing the mice. Therefore, it is very difficult to assess the treatment. Secondly, in this *Lgr5-DTR-GFP* mouse model, DT treatment causes off-target liver toxicity[1]. Therefore, this will likely interfere with the effect of LGR5 ablation, if on primary liver tumor.

Minor points:

Question 1: *Supl Fig 1 gives the numbers of mice but there is no legend. What is S and T?*

Answer: As suggested, the detailed information has been added (**Supplementary File 1, Page 1**).

Question 2: *in suppl Fig 4h, it would be more compelling if the authors show that the cultures start from single cells, instead of showing 1 organoids at later stage.*

Answer: We collected both single $LGR5^{+}$ and $LGR5^{-}$ cells and then traced the growth of them separately, up to two months. The corresponding figures are presented in **Supplementary Fig. 7h**.

Response To Reviewer #2

Major comments:

Question 1: *Major concerns relate to the missing translation into human disease. It would be crucial to investigate LGR5-expression in authentic human samples from different etiologies. The reviewer acknowledges the lack of suitable human antibodies. However, transcriptome expression could be assessed. Furthermore, prognostic implications should be determined (e.g. by using publically available data).In line with this, mechanistic investigations on LGR5 positive and negative putative CSCs should be performed to gain insights into the regulatory network of the cells (e.g. by RNA seq). Transcriptome profiles should also be evaluated in the context of the human disease. Of note, in addition to CTNNB1 mutations DEN is known to induce BRAF mutations, an alteration that is not frequently observed in human HCC. It would be interesting to investigate if the mutational status impairs the LGR5 expression of the tumors (publically available data could be explored)?*

Answers: We thank the reviewer for very helpful suggestions/comments. We agree that translation into human disease is vital. We now extended the LGR5 expression data and prognostic implications by investigating our own HCC cohort (from Erasmus Medical Center, **Fig. 2a-c**) and online databases (TCGA and others, **Supplementary Fig. 3a-b**). In the DEN-induced mouse model, mutations in *Ctnnb1*, *Braf* and *Egfr* genes have been reported[2,3]. We have now investigated the expression of *LGR5* stratified by these mutations in HCC patients from publically available datasets (TCGA and ICGC database). We observed that, *LGR5* expression is highly expressed in *CTNNB1* mutated, but not *BRAF* and *EGFR* mutated tumors (**Supplementary Fig. 4**). In addition, we also performed RNA-seq to compare the difference between untreated/5-FU treated $LGR5^+$ and $LGR5^-$ cells. The corresponding results were added in **Supplementary Fig. 6 and 9, Fig. 5h-j and Supplementary File. 3**.

Question 2: *The authors imply that the presence of CK19/HNF4a and LGR5 positive cells could result in both HCC as well as CCA development (page 7). This suggested bipotentiality is not underlined by the presented data. Please show representative IHC pictures demonstrating cholangiocellular differentiation in the model or provide further experimental evidence (e.g. by transplantation).*

Answers: CCA like organoid indeed initiate allograft tumors with cholangiocellular differentiation histology. All the HE, Gomori, CK19, AFP staining is now presented in **Supplementary File. 2**. Representative picture is shown as **Response Letter Figure 3**. This cholangiocellular differentiation histology is mainly observed in transplantation formed tumors, but not much in the primary tumor.

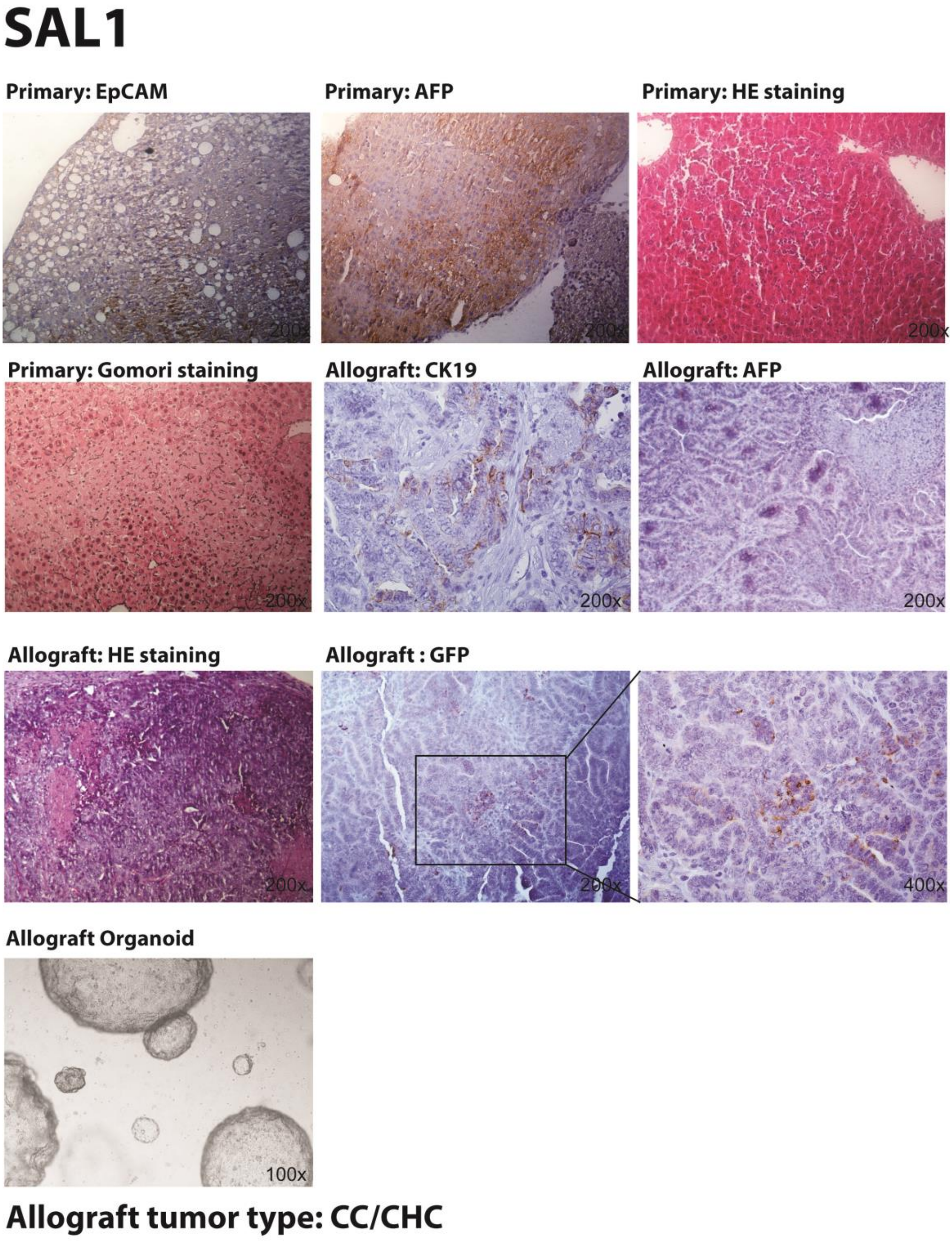

Response Letter Figure 3 | The allograft tumor histology, organoid morphology, corresponding primary tissue for tumor organoid.

Question 3: *In line with this, authors demonstrate that there are two different organoid-types in culture – a HCC and a CCC-like organoid type, reflected by co-expression of HNF4a and CK19. However, in further analyses different types are not distinguished. It would be important to assess the ratio between HCC and CCA organoids? Do the different organoids show distinct populations of LGR5 positive cells (frequency should be provided)? Is there a selection of phenotypes that is favored by the culturing or is e.g. CK19 expression induced by the artificial culture conditions (are other lineage markers expressed as well)? Do different types have the same or different tumor initiating capacity when transplanted into mice? And is there any difference in response to treatment comparing tumors derived from HCC-like organoids compared to CCC-like organoids? In addition, it is quite surprising that de-differentiated tumors express high levels of HNF4a. Please comment (what is the differentiation status of the tumors?).*

Answers: Among all the 14 allograft strains, we have 2 HCC-like strains, 7 CC/CHC-like strains and 5 strains that are very difficult to be classified. All the corresponding staining is presented in **Supplementary File. 2**.

We isolated $LGR5^+$ and $LGR5^-$ from the HCC-like and CC/CHC-like strains and have attempted to compare the difference between gene expression, especially for stem cell marker/tumor stem cell marker/differentiation marker. The results are shown in **Response Letter Figure 4**. HCC-like and CC/CHC-like strains indeed appear to have a different expression pattern in some stem cell/tumor stem cell markers.

In vitro, CC/CHC organoids are easier to be cultured, which is consistent with other studies[4]. We have observed that some primary organoids presenting a HCC-like morphology tend to stop proliferation within 1-3 passages, or the normal/CC-like organoids will over-grow to take over as the main phenotype. *In vivo*, the CC/CHC organoids are also easier to initiate tumor, compared to HCC-like organoids.

As demonstrated in our previous study[5], the sensitivity to different drugs varied dramatically among different liver tumor organoid strains (Figure 6 in the cited previous publication). At this point, we are still not sure what are the mechanisms behind the differential responsive to the treatment. However, we would like to stress that in this study we do not intend to classify these organoids as HCC or CC, but

rather collectively describe them as liver tumor organoids. Thus, we only describe as "HCC- or CC-like", in case it is necessary for better understanding. Firstly, because these DEN-induced mouse liver tumors may not fully recapitulate the spectrum of human liver cancer. Secondly, using liver tumor organoids is still very new and more insights shall be gained before we can clearly classify their types.

HNF4a is a marker that is expressed in normal hepatocytes and in tumor cells with hepatocellular differentiation. In dedifferentiated and in (some) poorly differentiated HCCs this marker is absent. As suggested, we now have consulted a professional liver (tumor) pathologist at our institute. He commented that the primary tumors, from which those 2 HCC-like tumor organoid strains derive, have a moderate differentiated HCC histology. Thus, we think this may potentially explain the expression of HNF4a.

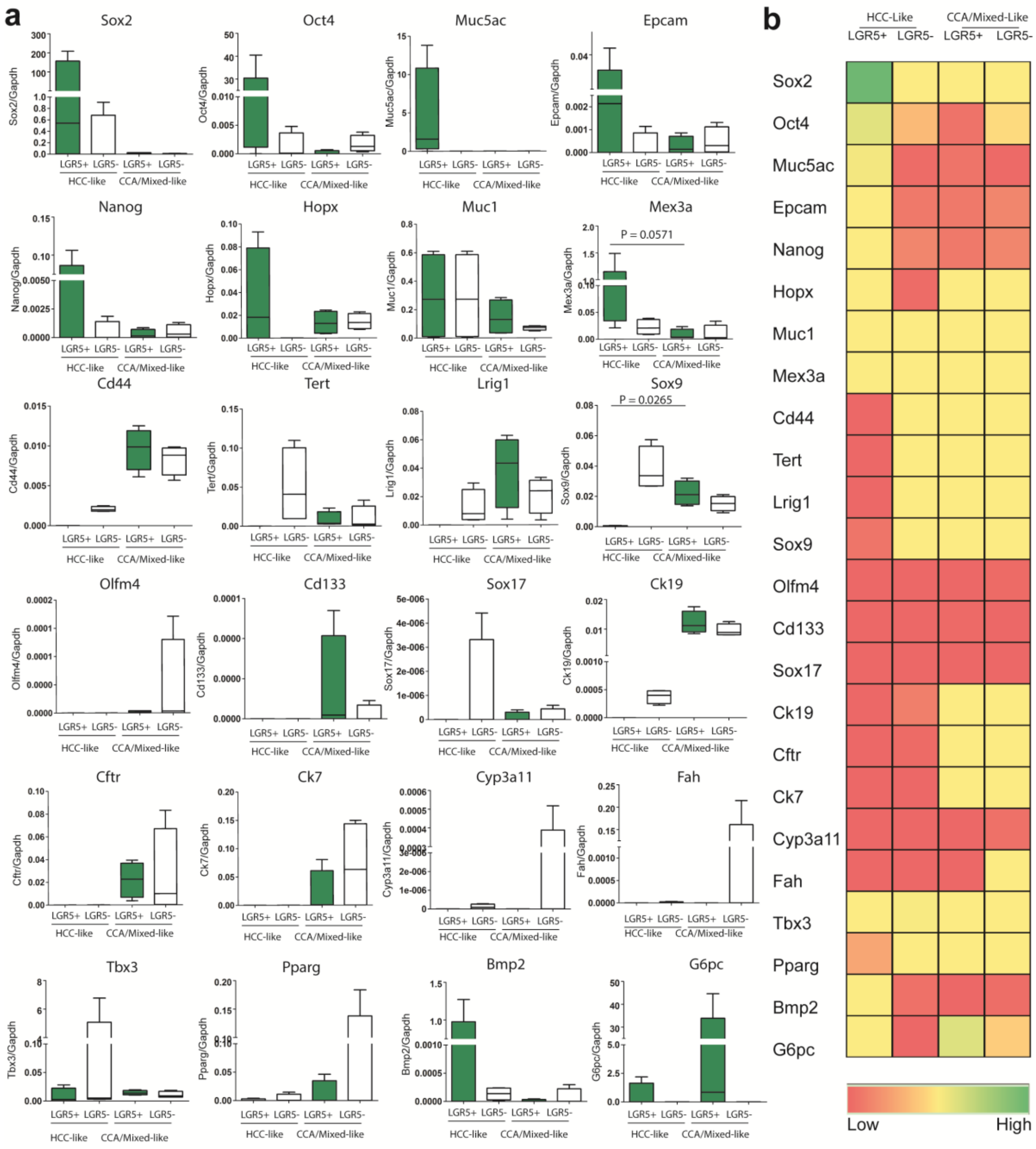

Response Letter Figure 4 |. HCC like organoid or CC/CHC like organoid showed differed expression pattern in stem cell markers and maturation markers. a, The expression of stem cell/tumor stem cell markers/maturation markers in HCC like or CC/CHC like organoid derived $LGR5^+/LGR5^-$ was analyzed using qRT-PCR and related to a reference gene. **b,** The heatmap showed the expression of stem cell/tumor stem cell markers/maturation markers in HCC like or CC/CHC like organoid derived $LGR5^+/LGR5^-$.

Question 4: *Depletion of LGR5 inhibited tumor growth similar to sorafenib and 5-FU treatment (Fig. 5i). It would be interesting to perform combination therapy of both compounds with LGR5-depletion to confirm potential synergistic effects (Fig 6).*

Answers: As suggested, we now have also performed the combination of DT with sorafenib (**Fig. 7**). However, the combination of LGR5 lineage ablation with sorafenib did not exert an enhanced anti-tumor effect, which is very different from the combination with 5-FU (**Fig. 8**). The possible reason could be that the enrichment of LGR5 cells is more robust by 5-FU compared to sorafenib treatment *in vivo* (**Fig. 5f**). This was further discussed in the discussion (**Page 18, Line 360-362**).

Question 5: *How was the number of transplanted cells controlled in the treatment experiments (Fig 4)?*

Answers: For this experiment (now **Fig. 5**), we treated the mice after forming tumors, and then analyzed the percentages of LGR5 cells in the tumor. Therefore, we used the protocol shown in **Response Letter Figure 5**, for controlling the number of transplanted cells. This approach is practically even better than digesting organoids into single cells to count cell numbers, for this specific experiment.

[REDACTED]

Response Letter Figure 5 |.Transplantation of tumor organoids.

Question 6: *Induction of LGR5 positive cells from LGR5 negative cells implicates plasticity of LGR5 putative CSCs. This might significantly affect the therapeutic potential (page 15). This should be mentioned and discussed. Given the role of CSC for tumor development, it would be interesting to dissect whether LGR5 depletion by diphtheria toxin would impair tumor development or just progression. Thus, it would be interesting to test this in the DEN model. However, the reviewer acknowledges that this might be beyond the scope of the here presented manuscript.*

Answers: As suggested, the plasticity of LGR5 cells now has been discussed (**Page 17-18, Line 344-351**). Indeed, we have mainly used the allograft mouse model, and investigated the effects on tumor initiation and growth. Regarding disease progression, we have technical challenges of depleting LGR5 cells by DT in the DEN-induced primary liver tumor model, because the DT treatment will induce off-target liver toxicity[1]. Therefore, this will likely interfere with the effect of LGR5 ablation, if on primary liver tumor.

Minor comment:

Question 1: *CCL4 treatment is not described. Please also provide a suitable reference for the DEN protocol applied.*

Answers: As suggested, the description about CCL4 treatment is added in the Materials and Methods (**Page 19, Line 389-392**). The reference for DEN protocol is also added in the Materials and Methods (**Page 19, Line 380 and 383**).

Question 2: *Figure 2e demonstrate only 2 allograft tumors highly positive for LGR5 cells after organoid transplantation. Further, comparison to primary tumors is not significant (p=0.3577). How many organoids retained LGR5+ cells? Is there a significant association for successful allograft transplantation? More details should to be provided.*

Answers: All the allograft organoids still retain the expression LGR5, and this can be seen in the **Response Letter Figure 6**. In addition, there seems to be no clear link between the successful allograft transplantation and the expression of $LGR5^+$ cells. The expression of $LGR5^+$ cells in primary tumor and allograft tumor is presented in **Supplementary File. 1**.

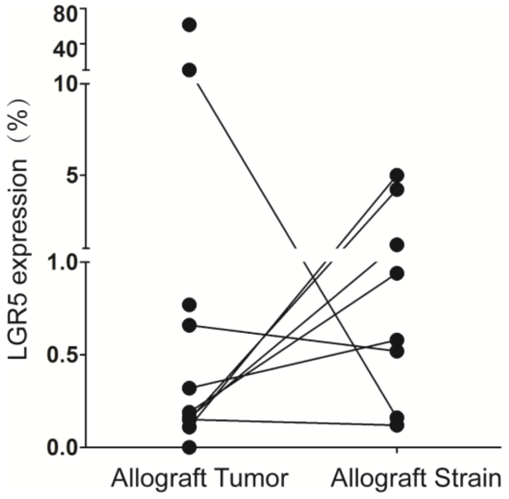

Response Letter Figure 6 |. The LGR5 expression in allograft tumors and corresponding allograft organoids strains. Allograft tumor Vs. organoid: 6.765 ± 5.558, n = 11 (3 out of 11 strains stop proliferation due to infection or unknown reasons) Vs. 1.594 ± 0.6754, n = 8.

Question 3: *Please note that FDA approved lenvatinib for first line treatment of HCC (page 14). Furthermore, regorafenib, cabozantinib, pembrolizumab as well as nivolumab are approved for 2nd line. Thus, sorafenib is not the only approved FDA drug, but still the standard of care. This should be corrected.*

Answers: As suggested, we correct the relative description from "the only FDA-approved drug for treating advanced HCC" to "the FDA-approved drug for treating advanced HCC" (**Page 11, Line 208**).

Question 4: *Magnification should be provided for all IHC/IF images*

Answers: As suggested, we added the magnification to all the IHC/IF images.

Reference:

1 Tian, H. *et al.* A reserve stem cell population in small intestine renders Lgr5-positive cells dispensable. *Nature* **478**, 255-259, doi:nature10408 [pii]10.1038/nature10408 (2011).

2 Connor, F. *et al.* Mutational landscape of a chemically-induced mouse model of liver cancer. *J Hepatol* **69**, 840-850, doi:S0168-8278(18)32162-7 [pii]10.1016/j.jhep.2018.06.009 (2018).

3 Dow, M. *et al.* Integrative genomic analysis of mouse and human hepatocellular carcinoma. *Proc Natl Acad Sci U S A* **115**, E9879-E9888, doi:1811029115 [pii]10.1073/pnas.1811029115 (2018).

4 Broutier, L. *et al.* Human primary liver cancer-derived organoid cultures for disease modeling and drug screening. *Nat Med* **23**, 1424-1435, doi:nm.4438 [pii]10.1038/nm.4438 (2017).

5 Cao, W. *et al.* Modeling liver cancer and therapy responsiveness using organoids derived from primary mouse liver tumors. *Carcinogenesis* **40**, 145-154, doi:5115755 [pii]10.1093/carcin/bgy129 (2019).

Reviewers' comments:

Reviewer #1 (Remarks to the Author):

Overall, the manuscript is improved from the previous version. While some of my concerns have been clarified and addressed, this reviewer has still concerns on the previous questions as well as in some of the new additions. Additionally, some claims are overstated and need to be revised. See below:

1) Reg my question_1, to how many animals got tumours and how many of these were Lgr5+? The authors now provide a Suppl File 1 with the data, but this is difficult to interpret. From tis data, it now seems that the tumours with more Lgr5+ expression do not give allografts. There is huge variability and no correlation between making allograft and Lgr5 expression. How is this explained in the context of the whole paper?
In addition, what is the Lgr5 expression? How is this number obtained? Is it RNA, number of cells? Along these lines, in the new Suppl Fig2g is not clear what has been counted.

2) The authors state that "A population of LGR5 cells was retained in these organoid cultures (Supplementary Fig. 5d)" yet they cannot proof whether this is the novo expression of Lgr5 due to high wnt in the medium or whether this is the cells present in the tumour. While now they proof that the Lgr5+ tumour cells make clones, they cannot rule out that the in vitro Lgr5 positiviity comes from the original cells or from de novo expression.
This same issue is recurrent later also after the sorafenib and 5fu treatment "Treatment of tumor organoids with sorafenib significantly
increased the percentage of LGR5 positive cells in the population (Fig. 5b-d). This effect became even more profound when the organoids were treated with the
chemotherapeutic agent, 5-fluoro-uracil (5-FU) (Fig. 5a-d)." Is this de novo expression or selection of Lgr5+ cells already in the tumour?
Similarly, the statement "the induction of LGR5+ from LGR5- liver cancer cells implicates cell plasticity of LGR5 CSCs," again suffers from the same flaw since formation of Lgr5+ from Lgr5- could just be because high wnt medium conditions and not true cellular plasticity, specially taking into account that they do not form tumours with the same ability, which suggests that this – to + conversion depends more on the environment.

This should be acknowledged and statements tone-down.

3) As in my previous comments, still the assumption that Lgr5- is the negative fraction of the FACS is wrong. Now the authors have done some fibroblast (Pdgfra) and endothelial (CD31) selection, on top of the immune (Cd45) selection, but still, since they do not sort for epithelial tumour cells, but only GFP+ vs GFP- they cannot exclude the presence of other cells that are negative for those markers. They should have a tumour marker and from there separate the Lgr5+ vs Lgr5- fractions. In addition, a 5% of non-epithelial cells sounds rather low, taking into account than in homeostasis the non-epithelial counterpart of the tissue accounts for an ~20%. The only thing that can be concluded is that Lgr5+ have clonogenic and tumorogenic potential, the comparison to Lgr5-, as performed, is flawed. Maybe the only way to address this would be by doing the same experiments with Lgr5+ and Lgr5- cells from cultures, where all is epithelial?

4) to answer my query whether the Lgr5+ cells after 5FU are the same or changed the authors perform RNA seq, but the data presented is merely decorative and does not give a hint as to which could be the mechanism for rewiring the transcriptome, as the authors claim. While this could represent a big task out of the scope of this paper, presenting or better discussing these findings and discussing some mechanism involved would add some value to the data presented.

On another note, and as above, is a matter of selection of cells that are Lgr5+ or they start expressing Lgr5 due to the treatment?

5) Reg the new human data, it is not very novel and totally expected:
Upregulation of Lgr5 expression in Ctnnb1 mutated compared to non-Ctnnb1 mutated is expected, as Lgr5 is a wnt target gene, and, additionally, had already been previously reported (Boyault et al Hepatology 2006). Similarly, that the survival is only affected in alcohol-related HCC is also expected from a canonical Wnt activated tumour (see Zuckman-Rossi gastroenterology 2015)

6) In a similar note, the following is an overstatement: "A straightforward interpretation of these results is that combining conventional therapy and LGR5+ targeted therapy would prove exceedingly useful for managing liver cancer in the clinic" since only a subset of the patients (b-cat mut) have elevated levels of Lgr5, only thouse would benefit from it, so IT would be better if that is re-phrased.

Reviewer #2 (Remarks to the Author):

This is the revised version of the manuscript by Cao et al.
The authors experimentally addressed several of the raised concerns. The revision strengthened the manuscript. However, several concerns have only been partially or not addressed at all. Importantly, question still relate to the validation of the finding in authentic human tumors. Besides a concerning lack of prognostic association, it seems that LGR5 plays a particular and dominant (exclusive?) role in alcohol related HCC (Figure 2 and Figure S3; clinico-pathological data of the in house cohort should be provided). REMARK guidelines should be further applied to confirm LGR5 as an independent prognostic factor. This should be discussed and explored in more detail since this is of major translational value. In line with this, gene expression profile of LGR5 positive cells still require confirmation and integration with the human disease. The association to CTNNB1 positive tumors is of further relevance. Can this be mechanistically explained (e.g. RNA seq data)?
The authors confirm that the different lineage tumors harbor different tumor-initiating capacity as well as marker profile. However, both types are not conclusively distinguished in the manuscript and, thus, might be biased. This should be clarified. Further, the provided immunostainings (supplementary file 2) are very difficult to assess, since no serial sections are shown and magnifications (not indicated in the figure) seems inconsistent. This should be improved.
It is still questionable if the transplantation approach indeed is superior to single cells. However, it would be particularly important to clarify if size of the included organoids has been controlled as well since this might have considerable impact on the viability and tumorigenicity of the cells.

Response to Reviewer #1:

<u>Question 1: Reg my question_1, to how many animals got tumours and how many of these were Lgr5+?</u>

<u>The authors now provide a Suppl File 1 with the data, but this is difficult to interpret.</u> ***<u>From tis data, it now seems that the tumours with more Lgr5+ expression do not give allografts. There is huge variability and no correlation between making allograft and Lgr5 expression. How is this explained in the context of the whole paper? In addition, what is the Lgr5 expression? How is this number obtained? Is it RNA, number of cells? Along these lines, in the new Suppl Fig2g is not clear what has been counted.</u>***

Answer: We apologize for the confusion of "Lgr5 expression". It is the percentage of Lgr5-expressing cells base on the positivity of GFP expression. We now have clarified this in Supplementary File. 1 as "LGR5-expressing cells (%)". We also clarified the description in Supplementary Fig. 2g and other relevant figures and figure legends.

Indeed, the percentage of Lgr5-expressing cells in primary tumors seems not clearly correlated to the ability of initiating allograft. Because primary tumors were first cultured into tumor organoids, and then these organoids were engrafted into mice for initiating allograft. Thus, this is an indirect assay (see illustration figure in Supplementary File. 1: Page 1) that may not fully reflect the initial status of the primary tumor.

<u>Question 2: The authors state that "A population of LGR5 cells was retained in these organoid cultures (Supplementary Fig. 5d)" yet they cannot proof whether this is the novo expression of Lgr5 due to high wnt in the medium or whether this is the cells present in the tumour. While now they proof that the Lgr5+ tumour cells make clones, they cannot rule out that the in vitro Lgr5 positiviity comes from the original cells or from de novo expression.</u>

<u>This same issue is recurrent later also after the sorafenib and 5fu treatment "Treatment of tumor organoids with sorafenib significantly increased the</u>

percentage of LGR5 positive cells in the population (Fig. 5b-d). This effect became even more profound when the organoids were treated with the chemotherapeutic agent, 5-fluoro-uracil (5-FU) (Fig. 5a-d)." Is this de novo expression or selection of Lgr5+ cells already in the tumour?
Similarly, the statement "the induction of LGR5+ from LGR5- liver cancer cells implicates cell plasticity of LGR5 CSCs," again suffers from the same flaw since formation of Lgr5+ from Lgr5- could just be because high wnt medium conditions and not true cellular plasticity, specially taking into account that they do not form tumours with the same ability, which suggests that this – to + conversion depends more on the environment. This should be acknowledged and statements tone-down.

Answer: We agree that the origins of these LGR5-expressing cells could be diverse. As suggested, we have revised as "A population of LGR5-expressing cells were again observed in these organoid culture" (**Page 8**, **Line 155-156**)**.**
Similarly, upon drug treatment, enrichment of $LGR5^+$ cells could be attributed to *de novo* generation and selection/expansion of the existing population (Fig. 5g). We agree that there are different possible mechanisms, including cell plasticity and the high levels of wnt, as the pointed out, regulating the status of LGR5 cells. As suggested, we have revised the statement in the Discussion (**Page 19**, **Line 382-384**), e.g. "This may implicates cell plasticity of LGR5 CSCs, but there could also be other mechanisms regulating the origin and expansion of LGR5 cells".

Question 3: As in my previous comments, still the assumption that Lgr5- is the negative fraction of the FACS is wrong. Now the authors have done some fibroblast (Pdgfra) and endothelial (CD31) selection, on top of the immune (Cd45) selection, but still, since they do not sort for epithelial tumour cells, but only GFP+ vs GFP- they cannot exclude the presence of other cells that are negative for those markers. They should have a tumour marker and from there separate the Lgr5+ vs Lgr5- fractions. In addition, a 5% of non-epithelial cells sounds rather low, taking into account than in homeostasis the non-epithelial counterpart of the tissue accounts for an ~20%. The only thing that can be concluded is that Lgr5+ have clonogenic and

tumorogenic potential, the comparison to Lgr5-, as performed, is flawed. Maybe the only way to address this would be by doing the same experiments with Lgr5+ and Lgr5- cells from cultures, where all is epithelial?

Answers: For the percentage of non-epithelial cells, the average percentage is not 5%, but 13%. The 5% only include the fibroblast and vascular cells, which was present in the previous version (R1) to specifically address the comment. Because the analysis in the original version already excluded immune cells. Therefore, the percentage for non-epithelial cells, including the fibroblast, vascular and immune cells, should be 13% in average (**Response Letter Figure 1a)**.

To further clarify this concern of the reviewer, as suggested, we performed organoid initiation assay and tumor formation assay with $Lgr5^+$ and $Lgr5^-$ cells isolated from tumor organoid culture, which do not contain the aforementioned non-epithelial cells. The corresponding results further support our conclusions and data are shown in the **Response Letter Figure 1b-f**.

a

Sample Code	The total number	The immuno cell number	The fibroblast cell number	The vescular cell number	Percentage of the immuno cells	Percentage of the fibroblast cells	Percentage of The vascular cells	The total percentage for fibroblast and vascular cells (%)	The total percentage for immune, fibroblast and vascular cells (%)
1	50000	4330	900	66	8,66	1,8	0,87	2,67	11,33002
2	50000	5345	1102	69	10,69	2,204	1,0222	3,2262	13,91622
3	50000	4869	1444	95	9,738	2,888	1,0851	3,9731	13,71109
4	67200	6873	3524	166	10,228	5,244	1,5131	6,7571	16,98481
5	30525	2263	1063	83	7,4136	3,4824	2,9717	6,4541	13,8677
6	100000	3062	2067	132	3,062	2,067	1,8684	3,9354	6,997365
7	100000	8132	1545	151	8,132	1,545	1,1704	2,7154	10,84736
8	100000	7949	3023	734	7,949	3,023	6,165	9,188	17,13696
Average								4,8649	13,099

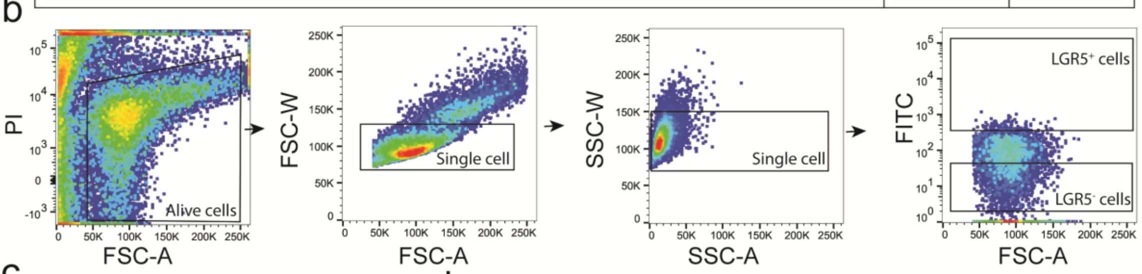

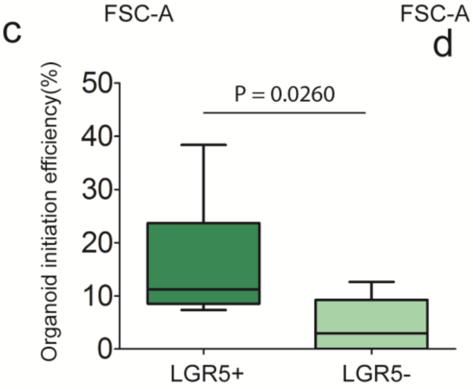

d

Sample Code	Organoid initiation efficiency (%)	
	LGR5+	LGR5-
1	18.77	12.61
2	7.33	3.23
3	38.37	2.71
4	8.91	0.00
5	9.30	0.00
6	13.18	8.14

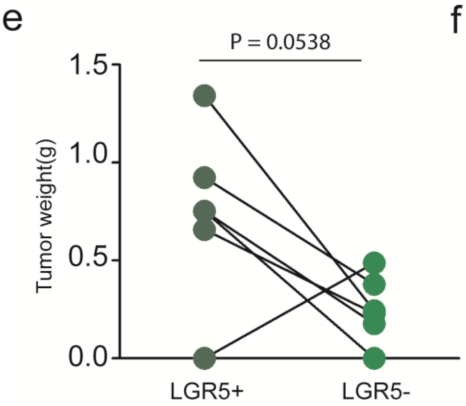

f

Sample Code	Tumor weight (g)	
	LGR5+	LGR5-
1	1.342	0.244
2	0.752	0.000
3	0.752	0.177
4	0.000	0.488
5	0.658	0.230
6	0.923	0.379

Response Letter Figure 1. a, The number and percentage of mesenchymal, endothelial and immune cells for individual tumors. **b**, The FACS sorting strategy for isolating LGR5^{+} and LGR5^{-} from organoid culture. **c**, The organoid initiation ability of LGR5^{+} cells (15.98 ± 4.783, n = 6) and LGR5^{-} cells (4.448 ± 2.036, n = 6), which were isolated from cultured tumor organoids. d, The exact initiation efficiency for LGR5^{+} cells and LGR5^{-} cells which were isolated from organoids. **e**, The weight of tumors initiated by LGR5^{+} cells (0.738 ± 0.178, n = 6) and LGR5^{-} cells (0.253 ± 0.0687, n =

6), which were isolated from organoids. f, The exact weight of tumors initiated by LGR5$^+$ cells and LGR5$^-$ cells, which were isolated from organoids.

Question 4: to answer my query whether the Lgr5+ cells after 5FU are the same or changed the authors perform RNA seq, but the data presented is merely decorative and does not give a hint as to which could be the mechanism for rewiring the transcriptome, as the authors claim. While this could represent a big task out of the scope of this paper, presenting or better discussing these findings and discussing some mechanism involved would add some value to the data presented. On another note, and as above, is a matter of selection of cells that are Lgr5+ or they start expressing Lgr5 due to the treatment?

Answer: As suggested, we have further elaborated and discussed the RNA seq data in this version (**Page 12-13, Line 241-253**). Regarding the origin of LGR5 cells upon treatment, these cells could be attributed to *de novo* generation and selection/expansion of the existing population (also see answer to Q2).

Question 5: Reg the new human data, it is not very novel and totally expected: Upregulation of Lgr5 expression in Ctnnb1 mutated compared to non-Ctnnb1 mutated is expected, as Lgr5 is a wnt target gene, and, additionally, had already been previously reported (Boyault et al Hepatology 2006). Similarly, that the survival is only affected in alcohol-related HCC is also expected from a canonical Wnt dactivated tumour (see Zuckman-Rossi gastroenterology 2015).

Answer: Indeed, our human data is not completely new, but as further complementary to previous studies (e.g. Boyault et al Hepatology 2006; Zuckman-Rossi gastroenterology 2015), and as support for the clinical relevance our experimental findings. This now has been further emphasized in the manuscript, and cited the previous publications.

As clinical studies were not able to demonstrate the function of LGR5 cells in liver cancer, the main focus and novelty of this study is that we have convincingly

demonstrated in mouse models that LGR5 marks tumor-initiating cells and can be therapeutically targeted in liver cancer.

<u>Question 6: In a similar note, the following is an overstatement: "A straightforward interpretation of these results is that combining conventional therapy and LGR5+ targeted therapy would prove exceedingly useful for managing liver cancer in the clinic" since only a subset of the patients (b-cat mut) have elevated levels of Lgr5, only thouse would benefit from it, so IT would be better if that is re-phrased.</u>

Answer: As suggested, we have revised as "Thus, combining conventional therapy and $LGR5^{+}$-targeted therapy deserve to be further explored for the treatment of liver cancer in the clinic. Conceivably, this approach could be more effective for subset of patients with high levels of LGR5 expression, such as CTNNB1 mutated or alcohol-related HCC patients." (**Page 16, Line 317-321**)

Response to Reviewer #2:

<u>Question 1: Importantly, question still relate to the validation of the finding in authentic human tumors. Besides a concerning lack of prognostic association, it seems that LGR5 plays a particular and dominant (exclusive?) role in alcohol related HCC (Figure 2 and Figure S3; clinico-pathological data of the in house cohort should be provided). REMARK guidelines should be further applied to confirm LGR5 as an independent prognostic factor. This should be discussed and explored in more detail since this is of major translational value.</u>

Answer: Indeed, REMARK is a highly recognized guideline, but specific for tumor marker prognostic studies[1]. Our clinical cohort (Fig. 2) as well as the cohorts (Supplementary Fig. 3) retrieved from publically available are not specifically designed for this type of studies, and therefore, are likely not applicable for the REMARK guideline. In our study, we do not conclude whether LGR5 is an independent prognostic biomarker in liver cancer. Of course, as pointed out, this aspect could be interesting for future investigations in large and well-designed clinical studies. In this study, the clinical data showed the differential expression of LGR5 in tumor and adjacent tissues, and the potential association with clinical outcomes. These results are intended to support the clinical relevance of our experimental findings. However, these clinical investigation does not reveal the function of LGR5 cells. And, most importantly, this study has demonstrated in mouse models that LGR5 marks tumor-initiating cells and can be therapeutically targeted in liver cancer. We now have further clarified these points in the Discussion (**Page 16-17, Line 328-332**).

<u>Question 2: In line with this, gene expression profile of LGR5 positive cells still require confirmation and integration with the human disease. The association to CTNNB1 positive tumors is of further relevance. Can this be mechanistically explained (e.g. RNA seq data)?</u>

Answer: As suggested, we further analyzed the gene expression profile of LGR5$^+$ cells and the corresponding discussion is added in the manuscript **(Page 9**, **Line 158-179**).

In addition, it is not very surprising that CTNNB1-mutant tumors appear to have higher levels of LGR5 expression mechanistically. LGR5 has been identified as a β-catenin target gene in the intestine[2], and is also upregulated in response to Wnt/β-catenin signaling both in the normal liver and cancers thereof[3,4]. To make this more clear to the reader we have added the following text to the paper: “This is in line with LGR5 being a β-catenin target gene both in the intestine and liver[2-4]” (**Page 7, Line 130-131**).

<u>Question 3: The authors confirm that the different lineage tumors harbor different tumor-initiating capacity as well as marker profile. However, both types are not conclusively distinguished in the manuscript and, thus, might be biased. This should be clarified.</u>

Answer: In the setting of human liver cancer, a previous study has classified the organoid types based on the origin of the primary tumor: hepatocellular carcinoma (HCC), cholangiocarcinoma (CC) and combined HCC/CC (CHC) tumors[5]. However, as we have emphasized in the manuscript, the primary tumors from our mouse model do not fully recapitulate the spectrum of human liver tumor types. Therefore, we classified our organoids as “HCC-like” or “CC-like” types. We now have clarified the approaches of how to propose these “-like” phenotypes in the Methods (**Page 23, Line 462-463; Page 24, Line 481-483**).

<u>Question 4: Further, the provided immunostainings (supplementary file 2) are very difficult to assess, since no serial sections are shown and magnifications (not indicated in the figure) seems inconsistent. This should be improved.</u>

Answer: As suggested, serial sections were performed for all the staining and new figures are presented in the **Supplementary File 2**.

<u>Question 5: It is still questionable if the transplantation approach indeed is superior to single cells. However, it would be particularly important to clarify if size of the included organoids has been controlled as well since this might have considerable impact on the viability and tumorigenicity of the cells.</u>

Answer: Indeed, we are not able to conclude which approach is superior. However, the use of transplanting fragmented organoids is more about technical convenience.
As suggested, we now have calculated the size of organoid fragmentation from three independent experiments. The average size is 50.38 ± 1.716 µm, n = 239 (range from 5 ~ 150 µm) (**Response Letter Figure 2**). These information now has been included in the Methods (**Page 24, Line 476-477**).

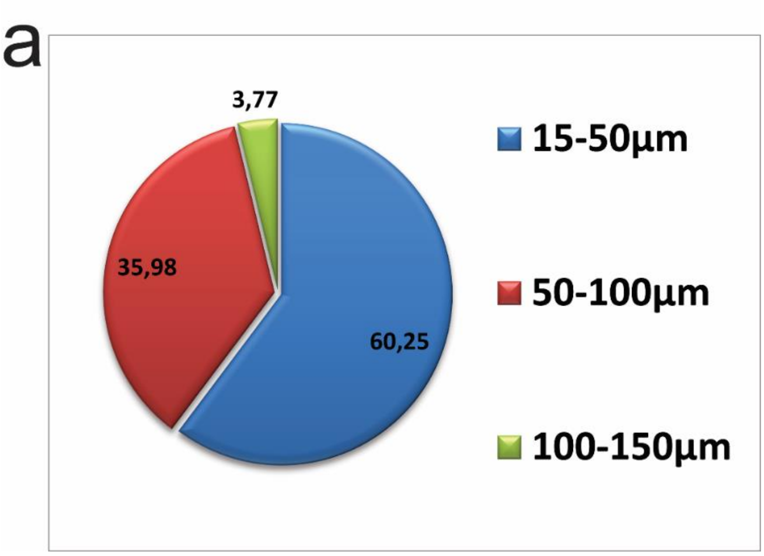

Response Letter Figure 2 The distribution of the size of the organoid fragmentation.

Reference:

1 Sauerbrei, W., Taube, S. E., McShane, L. M., Cavenagh, M. M. & Altman, D. G. Reporting Recommendations for Tumor Marker Prognostic Studies (REMARK): An Abridged Explanation and Elaboration. *J Natl Cancer Inst* **110**, 803-811, doi:5032903 [pii]10.1093/jnci/djy088 (2018).

2 Barker, N. *et al.* Identification of stem cells in small intestine and colon by marker gene Lgr5. *Nature* **449**, 1003-1007, doi:nature06196 [pii]10.1038/nature06196 (2007).

3 Yamamoto, Y. *et al.* Overexpression of orphan G-protein-coupled receptor, Gpr49, in human hepatocellular carcinomas with beta-catenin mutations. *Hepatology* **37**, 528-533, doi:10.1053/jhep.2003.50029S0270913902141437 [pii] (2003).

4 Huch, M. *et al.* In vitro expansion of single Lgr5+ liver stem cells induced by Wnt-driven regeneration. *Nature* **494**, 247-250, doi:nature11826 [pii]10.1038/nature11826 (2013).

5 Broutier, L. *et al.* Human primary liver cancer-derived organoid cultures for disease modeling and drug screening. *Nat Med*, doi:nm.4438 [pii]10.1038/nm.4438 (2017).

REVIEWERS' COMMENTS:

Reviewer #1 (Remarks to the Author):

This version is indeed addressing my previous concerns. I have no further questions, yet I would suggest the following changes to the manuscript to facilitate the understanding of the concepts explained to the general public:

-please add some discussion that reflects that the percentage of Lgr5-expressing cells in primary tumors does not correlate entirely with tumour initiating potential. The explanation given by the authors, that the cells are cultured first, seems a very plausible hypothesis indeed. It would be important, though, that this point is made clear in the text so readers are not confused with the idea that only Lgr5+ liver tumour cells are the only ones that could initiate liver tumours.

Along the same lines, I would suggest the following addition to the new change ""This may implicates cell plasticity of LGR5 CSCs, but there could also be other mechanisms regulating the origin and expansion of LGR5 cells, as for example, changes in the culture environment ".

Reviewer #2 (Remarks to the Author):

This is the revised version of the manuscript. While some aspects have been adressed and clarified, several of the raised concerns, particularly related to the translation into human disease as well as the prognostic impact and etiological association of the findings, remain only partially or not addressed at all.

Response to Reviewer #1:

<u>Question 1: Please add some discussion that reflects that the percentage of Lgr5-expressing cells in primary tumors does not correlate entirely with tumour initiating potential. The explanation given by the authors, that the cells are cultured first, seems a very plausible hypothesis indeed. It would be important, though, that this point is made clear in the text so readers are not confused with the idea that only Lgr5+ liver tumour cells are the only ones that could initiate liver tumours.</u>

<u>Along the same lines, I would suggest the following addition to the new change ""This may implicates cell plasticity of LGR5 CSCs, but there could also be other mechanisms regulating the origin and expansion of LGR5 cells, as for example, changes in the culture environment ".</u>

Answer: we thank the reviewer very much for his/her interests in our study. The comments are very useful for revising the manuscript. As suggested, we have added the discussion (**Page 18, Line 365-369**) and also revised the corresponding sentence (**Page 19, Line 385**).

Response to Reviewer #2:

<u>This is the revised version of the manuscript. While some aspects have been adressed and clarified, several of the raised concerns, particularly related to the translation into human disease as well as the prognostic impact and etiological association of the findings, remain only partially or not addressed at all.</u>

Answer: Firstly, we thank the reviewer very much for his/her interest in our study. As suggested, we have revised the statement about the clinical relevance part in the manuscript.
